# Exploratory study of the association in the United Kingdom between hypertension and inorganic arsenic (iAs) intake from rice and rice products

**DOI:** 10.1007/s10653-020-00573-8

**Published:** 2020-04-28

**Authors:** Lingqian Xu, David A. Polya

**Affiliations:** Department of Earth and Environmental Sciences and Williamson Research Centre for Molecular Environmental Science, University of Manchester, Manchester, M13 9PL UK

**Keywords:** Inorganic arsenic, Rice, Hypertension, Cross-sectional analysis, Exposure science

## Abstract

Hypertension risks arising from chronic exposure to inorganic arsenic (iAs) are well documented. Consumption of rice is a major iAs exposure route for over 3 billion people; however, there is a lack of epidemiological evidence demonstrating an association of hypertension risks with iAs intake from rice, especially in areas where there is little exposure from drinking water but a growing demand for rice intake. To address this, we conducted an individual-level cross-sectional analysis to quantify the extent to which daily iAs intake from rice and rice products (E-iAs_ing,rice_) modifies the association between hypertension risks and previously well-established risk factors. The analysis was based on secondary dietary, socio-demographic and health status data of 598 participants recorded in the UK National Diet and Nutrition Survey 2014–2016. E-iAs_ing,rice_ and five blood pressure endpoints were derived with potential associations explored through generalized linear models. According to the results, a negative but not significant relationship was found between hypertension risks and E-iAs_ing,rice_ after adjusting for major risk factors, notably age, gender, diabetes and obesity, with relatively higher risks being observed for male, middle-aged, overweight, alcohol consumer or Asian or Asian British, Black or Black British and mixed ethnic groups. Though inconclusive and mainly limited by potential incomplete adjustment for major confounders and intrinsic disadvantages of a cross-sectional design, this study was the first quantifying the individual level dose–response relationship between E-iAs_ing,rice_ and hypertension risks and is consistent with previous studies on the limited associations of hypertension with low-level arsenic exposure from drinking water. Larger scale cohort studies are indicated to quantify the association but in any event it is likely to be weak.

**Electronic supplementary material:**

The online version of this article (10.1007/s10653-020-00573-8) contains supplementary material, which is available to authorized users.

## Introduction

Arsenic (As), which originates from either geological or anthropogenic sources (Polya and Lawson 2016), is reported to be ubiquitously present in the environment (Bundschuh et al. 2012; Huda et al. 2014). Arsenic has been widely recognized as a human carcinogen for over 50 years (Currie 1947; Frost 1969; Hueper 1967; Polya and Middleton 2017), and it is especially the case for inorganic arsenic (iAs), which is predominant or important in soil, water, air and some foodstuffs (Bae et al. 2017; Currier et al. 2014; Diane et al. 2013; Molin et al. 2015; Yáñez et al. 2015). According to the US Department of Health And Human Services et al. (2007), people may be exposed via various pathways, notably inhalation, dermal contact, ingestion and through parenteral routes. Ingestion of iAs through drinking water is a particularly important exposure route, especially for people living in certain geographic regions, such as parts of the Indian sub-continent, south-east and east Asia (Chakraborti et al. 2018; McCarty et al. 2011; Polya and Middleton 2017; Xia and Liu 2004).

Based upon a substantial literature of research on medicinal, epidemiological, experimental toxicology and in vitro mechanistic aspects of iAs, iAs exposure from drinking water can be closely causally connected with the risk of cardiovascular disease (CVD) (Tsuji et al. 2014). For example, individuals who were in direct contact with high level well water iAs in Bangladesh were reported to suffer increased risk of mortality from both ischemic heart disease and cerebrovascular disease (Chen et al. 2011). In addition, a huge amount of empirical evidence supports the impacts of drinking water iAs on the risk of hypertension, specifically both higher diastolic blood pressure and systolic blood pressure (Hall et al. 2017; Hossain et al. 2017; Kunrath et al. 2013). Similar positive associations with exposure to iAs through drinking water were also observed for the risk of CVD markers (Wu et al. 2012) and stroke (Rahman et al. 2014).

Particularly, in areas where there is little or no iAs exposure from drinking water, iAs exposure is, more importantly, from everyday foods (European Food Safety Authority 2009; Meharg and Zhao 2012; Mondal and Polya 2008; Schoof et al. 1999). Because of the joint impact of its physiology along with flooded paddy field geochemistry, rice, in many cases, contains substantially more As than other major staples (Meharg et al. 2008), and is therefore particularly regarded as an important source of iAs exposure (Food and Agriculture Organization of the United Nations 2008). This is especially the case for areas, notably Bangladesh, India, South-East Asia, southern China and parts of South America (Meharg and Zhao 2012), where the majority of residents exposed with high As from drinking water and mainly consume rice—as opposed to other staples—in everyday meals. In regions, ranging from USA (Gossai et al. 2017), Spain (Signes-Pastor et al. 2017) to the UK (Meharg et al. 2007) and Australia (Islam et al. 2017) where there is little exposure from drinking water, rice is not regarded as the daily staple for the majority of the population but nevertheless, its role in iAs exposure cannot be ignored particularly for sub-populations taking rice at a relatively high rate (Awata et al. 2017; Cleland et al. 2009; Mantha et al. 2017). It has been reported that rice consumption is increasing in the UK due to the changes of ethnic distribution and food diversification (Schenker 2012). Although the intake of rice has become the main iAs exposure pathway for more than three billion individuals around the world, to the best of our knowledge, little epidemiological evidence exists to demonstrate CVD risks arising from iAs exposure from rice and rice products (Torres-Escribano et al. 2008).

Hypertension, a common form of CVD, is not only the leading cause of morbidity and mortality in the world with a global prevalence of approximately 40% (Hall et al. 2017; World Health Organization 2011), but also, more seriously, recognized as a major risk factor for some other CVD types (Kannel 1996; Sowers et al. 2001; Wang et al. 2011), including stroke (Hu and Balakrishnan 2005) and ischemic heart disease (Collins and MacMahon 1994; Stamler et al. 1993). Considering a number of factors, such as age, obesity, gender, smoking status, alcohol consumption and sodium intake (Biino et al. 2013; He et al. 2018; NHLBI Obesity Education Initiative Expert Panel on the Identification Evaluation and Treatment of Obesity in Adults (US) 1998), that are widely known to be important indicators of hypertension, the effect of iAs intake from rice and rice products is likely to be less important. However, given the large number of people exposed to iAs, and the high prevalence of morbidity and mortality due to hypertension worldwide, even a small association might result in hundreds of thousands of additional hypertension cases (Ferguson et al. 2018; Gao et al. 2018; Li et al. 2015; World Health Organization 2011). It is, therefore, of importance to quantify the contribution of rice and rice products on iAs intake and assess its relation with hypertension risk.

In this study, we conducted an individual level cross-sectional analysis to quantify the extent to which daily iAs intake from rice and rice products (E-iAs_ing,rice_) modifies the association between hypertension risks and previously well-established risk factors. In addition to general hypertension (abnormally high arterial blood pressure), four other blood pressure parameters, viz. mean values of diastolic blood pressure (DBP), systolic blood pressure (SBP), arterial pressure [AP, defined as 1/3 × (SBP + 2 × DBP)] and pulse pressure (meanPulse, valid pulse readings), which are associated with an increased risk of vascular disease have also been included as indicators of hypertension risks (Chen et al. 2007; Lelong et al. 2019; Rahman 2002; US Department of Health And Human Services et al. 2007).

The objectives of the study were to (1) quantify the importance of E-iAs_ing,rice_ and other confounders to the variability of hypertension risks; (2) model the relationships between E-iAs_ing,rice_ and hypertension risks; (3) test the effects modification of several well-established risk factors on the association between E-iAs_ing,rice_ and hypertension risks, identifying vulnerable subgroups. We further discussed our exploratory findings, and in the light of these, made recommendations for future work.

## Methods

We explored the extent to which E-iAs_ing,rice_ modifies the association between hypertension risks and previously well-established risk factors using a repeated cross-sectional design within the National Diet and Nutrition Survey Rolling Programme from April 2014 through August 2015 for Year 7 and April 2015 through August 2016 for Year 8 (NDNS RP 7–8) (MRC Elsie Widdowson Laboratory and NatCen Social Research 2019).

Details of the parent survey NDNS RP 7–8 may be found elsewhere (MRC Elsie Widdowson Laboratory and NatCen Social Research 2019). In summary, NDNS RP 7–8 is a national population-based survey of food consumption and nutritional status of people aged 1.5 years and older living in private households in the UK. Information is gathered on demographic, socio-economic, behaviour, dietary and health status through door-to-door recruitment, in-person interviews, along with comprehensive data collection, physical measurements and a food diary. The present study used secondary data from the most recent data cycles (from April 2014 through August 2015 for Year 7 and April 2015 through August 2016 for Year 8) because these two surveys provide the latest source of high-quality nationally representative data on the types and quantities of different rice and rice products consumed by individuals and their health status.

Because our present study only used publicly available and anonymized data from NDNS RP 7–8, we required no further ethical approval for our study, noting that the authors of the NDNS RP 7–8 study had themselves obtained ethical approval for their study from the Cambridge South NRES Committee (Ref. No. 13/EE/0016).

### Study population

We firstly extracted secondary data related to all the participants in NDNS RP 7–8 (*N* = 2723). NDNS RP 7–8 was carried out in all four countries of the UK and was designed to be representative of the UK population, selecting participants using a cross-sectional, multistage and random sampling design. Also, their fieldwork was conducted throughout the year (from April 2014 through August 2015 for Year 7 and April 2015 through August 2016 for Year 8) in order to take into account the potential seasonal variations in food consumption. Details about the recruitment of participants can be found in MRC Elsie Widdowson Laboratory and NatCen Social Research (2019). During the study period, a representative sample of 2723 participants aged 1.5 and older was recruited.

As the dietary pattern, some socio-demographic characteristics, general health condition and blood pressure status of children, pregnant and breastfeeding women may change and may be different from the general population (Attorp et al. 2014; MRC Elsie Widdowson Laboratory and NatCen Social Research 2019; Yoder et al. 2009), and this study excluded participants based on the following criteria: (1) women who were pregnant and breastfeeding (*N* = 0); (2) people younger than 16 (*N* = 1074); and (3) participants with missing data (*N* = 1051), in relation to SBP (681), DBP (681), AP (681), general hypertension (681), meanPulse (524), qual7 (qualifications gained) (49), ethgrp5 (ethnic group) (8), eqv3 (equalized household income) (243), cigsta3 (cigarette smoking status) (8), dnoft (frequency of alcohol consumption in past 12 months) (9), bmival (valid BMI) (149), whgval (waist-hip ratio groups) (543), Diabetes.combined (whether respondent is diabetic) (768) and SalHowC (how often salt added during cooking) (24). After such exclusions, the final population size used in the present study was 598.

### Data collection

NDNS RP 7–8 dataset was collected from 2-stage interviewer visits to each household covering face-to-face interviews, self-completion questionnaires, a food diary and physical measurements. Details about the related interviews, questionnaires, dietary record and physical measurements can be found elsewhere (MRC Elsie Widdowson Laboratory and NatCen Social Research 2019). A brief description of variables included in the present study is illustrated in Table 1, with a detailed description being summarized and provided in Table S1 (For some characteristics, individuals were regrouped due to the small number of subjects in some categories).

**Table 1:** Description of variables included in the present study

Variable number	Variable name/code	Description	Variable type
1	Age	Age of respondent 16 + year (16–34; 35–49; 50–64; 65 + years)	Categorical
2	AP	Mean arterial pressure (mmHg)	Continuous
3	bmival	Valid BMI group (underweight; normal; overweight; obese)	Categorical
4	cigsta3	Cigarette smoking status (Current cigarette smoker; ex-regular cigarette smoker; never regular cigarette smoker)	Categorical
5	DBP add 10	Omron valid mean diastolic blood pressure (DBP) incremented by 10 mmHg is added if anti-hypertension medication is taken (mmHg)	Continuous
6	Diabetes.combined	Whether respondent is diabetic (Yes; no)	Categorical
7	dnoft	Frequency of alcohol consumption in past 12 months (5 or 7 days a week; 3 or 4 days a week; once or twice a week; once or twice a month; once every couple of months; once or twice a year; not at all in the last 12 months/non-drinker)	Categorical
8	E-iAs_ing,grain_	Daily inorganic arsenic (iAs) intake from grain and grain-based products (µg/person/day)	Continuous
9	E-iAs_ing,rice_	Daily iAs intake from rice and rice products (µg/person/day)	Continuous
10	E-iAs_ing,water_	Daily iAs intake from drinking water (µg/person/day)	Continuous
11	EnergyDkJ	Intake of total energy per day (KJ) for diet only (grouped into quartiles based on the distributions of energy intake level in the study population)	Categorical
12	eqv3	Equivalized household income (£) [Lowest tertile (≤ 17,500); middle tertile (> 17,500 ≤ 32,216); highest tertile (> 32,500)]	Categorical
13	ethgrp5	Ethnic group, 5 groups (White; mixed ethnic group; Black or Black British; Asian or Asian British; any other group)	Categorical
14	FatgD	Intake of fat per day (g) for diet only (grouped into quartiles based on the distributions of fat intake level in the study population)	Categorical
15	FolateugplussuppsD	Intake of folate (µg) per day for both diets and supplements (grouped into quartiles based on the distributions of folate intake level in the study population)	Categorical
16	General hypertension	Whether participants were diagnosed as general hypertension (Yes; no)	Categorical
17	GlucosegD	Intake of glucose per day (g) for diet only (grouped into quartiles based on the distributions of glucose intake level in the study population)	Categorical
18	HessCon	Whether have any physical/mental health condition/illnesses for 12 months or more (Yes; no)	Categorical
19	meanPulse	Mean value of the three valid pulse pressure readings (mmHg)	Continuous
20	MN	Daily intake of several micro-nutrients (Participants with 0–3 nutrients ≥ the mean intake of the accordingly nutrients; participants with 4–7 nutrients ≥ the mean intake of the accordingly nutrients; Participants with 8–11 nutrients ≥ the mean intake of the accordingly nutrients; participants with 12–15 nutrients ≥ the mean intake of the accordingly nutrients; participants with 16–18 nutrients ≥ the mean intake of the accordingly nutrients)	Categorical
21	NumChild	Number of Children aged between 0 and 15 (Have no child; have 1–2 children; have 3–4 children; have 5–6 children)	Categorical
22	ProteingD	Intake of protein per day (g) for diet only (grouped into quartiles based on the distributions of protein intake level in the study population)	Categorical
23	qual7	Qualifications gained (Degree or equivalent; Higher education, below degree level and GCE, A level or equivalent; GCSE grades A–G or equivalent/commercial qualifications/apprenticeship; Foreign or other qualifications and no qualifications and still in FT education)	Categorical
24	Quarter	Fieldwork quarter (Season 1: Apr–Jun; season 2: Jul–Sep; season 3: Oct–Dec; season 4: Jan–Mar)	Categorical
25	Region	Country people live (England: Central/Midlands; England: North England: South (including London); Northern Ireland and Scotland)	Categorical
26	SalHowC	How often salt added during cooking (Never; sometimes and usually; always)	Categorical
27	SBP add 10	Omron valid mean systolic blood pressure (SBP) incremented by 10 mmHg is added if anti-hypertension medication is taken (mmHg)	Continuous
28	Sex	Gender (Male, female)	Categorical
29	SodiummgD	Intake of sodium per day (mg) for diet only (grouped into quartiles based on the distributions of sodium intake level in the study population)	Categorical
30	surveyyr	NDNS RP 7–8 survey year [Year 7 (2014–2015); Year 8 (2015–2016)]	
31	whgval	Waist-hip ratio groups (Normal weight; overweight; obesity)	Categorical
32	WrkStat	Economic status (In full or part-time employment; Going to school or college full-time (including on vacation) and not working at present)	Categorical

Variables including age, bmival, dnoft, EnergyDkJ, FatgD, FolateugplussuppsD, GlucosegD, MN, NumChild, ProteingD, qual7, region, SalHowC, SodiummgD, whgval and WrkStat were regrouped based on the NDNS RP 7–8 (MRC Elsie Widdowson Laboratory and NatCen Social Research 2019)

Variables ranging from AP, DBP add 10, E-iAs_ing,rice_, E-iAs_ing,water_, E-iAs_ing,grain_ to meanPulse and SBP add 10 were calculated based on the NDNS RP 7–8 dataset (MRC Elsie Widdowson Laboratory and NatCen Social Research 2019)

Detailed calculation and grouping information for these variables are listed in Table S1

### Daily iAs intake from rice and rice products (µg/person/day), E-iAs_ing,rice_

Daily iAs intake from rice and rice products (µg/person/day), E-iAs_ing,rice_, was estimated using the NDNS RP 7–8 reported consumption level by Eqs. (1) and (2) followed (Awata et al. 2017):

1 $${\text{E-iAs}}_{\text{ing,rice}} = \mathop \sum \limits_{i} {\text{RC}}_{i} \times {\text{C}}_{{{\text{rice,}}i}} \times ( 1- {\text{LOSS}}_{\text{cooking}} )$$

2 $${\text{RC}}_{i} = \frac{{\mathop \sum \nolimits_{1}^{n} {\text{DRC}}_{i} }}{n}$$

where RC_*i*_ is the average daily consumption (kg/day) of rice and rice product, *i*, during the food diary (ready-to-eat), *C*_rice,*i*_ is the iAs concentration (µg/kg) of the rice and rice product, *i* (ready-to-eat or raw), LOSS_cooking_ is the estimated proportion of iAs lost from rice and rice products upon cooking, *n* length of the food diary [3 days (*n *= 3) or 4 days (*n *= 4)], DRC_*i*_ consumption rate (kg/day) of rice and rice product, *i*, in each day during the food diary.

Food commodity consumption was calculated for all food diary periods, and the average consumption value was used for average daily commodity consumption level of each survey participant. Values for *C*_rice,*i*_ were estimated from those reported by European Food Safety Authority (2014). LOSS_cooking_ was estimated as 5% across all not ready-to-eat foods based upon the study of Mwale et al. (2018) and as 0% for ready-to-eat foods.

### Blood pressure endpoints

Several blood pressure endpoints, including SBP, DBP, meanPulse, SBP add 10, DBP add 10, AP and general hypertension, all of which are associated with an increased risk of vascular ill-health (US Department of Health And Human Services et al. 2007) were included as our target outcomes.

Mean values of three valid SBP, DBP and pulse pressure readings were used to represent SBP, DBP and meanPulse level, respectively. Given that people taking anti-hypertension medications have controlled and likely artificially low blood pressure (Banda et al. 2010; Zamora-Kapoor et al. 2018), we addressed this by adding a constant 10 mmHg to the SBP and DBP for participants with such medications as SBP add 10 and DBP add 10 (cf. Mordukhovich et al. 2012). AP is defined as the average pressure in a patient’s arteries during one cardiac cycle, calculated as (SBP add 10 + 2 × DBP add 10)/3 (Chen et al. 2007; Lelong et al. 2019). General hypertension is defined as a SBP ≥ 140 mmHg, or a DBP ≥ 90 mmHg and/or under regular treatment with anti-hypertension medications (Among the total 2723 NDNS RP 7–8 participants, 245 of them were identified as taking anti-hypertension medications at the time of the interview) (Rahman 2002).

Considering the final population size used in the present study (598), except for DBP add 10 and meanPulse, the statistical power of the findings for all the other blood pressure endpoints was higher than 0.8 (data not shown).

### Definition of confounders

Variables which are well recognized as important predictors of either hypertension risks or As intake should be considered when testing the association between E-iAs_ing,rice_ and hypertension risks (Banda et al. 2010; Biino et al. 2013; Chen et al. 2007; Jarrah et al. 2018; Kim and Lee 2019; Lelong et al. 2019; Mohtasham-Amiri et al. 2018; Re 2009).

In general, confounding information in the present study (Sex (gender), age (age of respondent 16 + years old), ethgrp5, region (country people live), HessCon (whether have any physical/mental health condition/illnesses for 12 months or more), NumChild (number of Children aged between 0 and 15), qual7, Diabetes.combined, WrkStat (economic status), Quarter (fieldwork quarter), surveyyr (NDNS RP 7–8 survey year), eqv3, cigsta3, dnoft, bmival, whgval, SalHowC, MN (daily intake of several micro-nutrients including potassium, calcium, magnesium, iron, copper, zinc, retinol, vitamin A, vitamin D, Vitamin e, thiamin, riboflavin, niacin equivalent, vitamin B6, vitamin B12, vitamin, iodine, selenium), EnergyDkJ (intake of total energy per day (KJ) for diet only), ProteingD (intake of protein per day (g) for diet only), FatgD (intake of fat per day (g) for diet only), GlucosegD (intake of glucose per day (g) for diet only), SodiummgD (intake of sodium per day (mg) for diet only) and FolateugplussuppsD (intake of folate (µg) per day for both diets and supplements), E-iAs_ing,water_ (daily iAs intake from drinking water) as well as E-iAs_ing,grain_ (daily iAs intake from grain and grain-based products) was collected by NDNS RP 7–8 trained staffs and nurses during the two stages interviews coupled with a series of questionnaires, physical measurements and a food diary.

Some demographic, behavioural and socio-economic risk factors, such as Sex, age, ethgrp5, region, HessCon, NumChild, qual7, Diabetes.combined, WrkStat, Quarter, surveyyr and eqv3, were obtained during detailed background interview. cigsta3 and dnoft were included through smoking and drinking self-completion questionnaires.

Trained staff measured participants’ height and weight and then calculated BMI during the first stage interview based on the protocols from MRC Elsie Widdowson Laboratory and NatCen Social Research (2019). Standard international cut-off points were used for bmival, grouping participants into underweight (< 18.5 kg/m^2^), healthy weight (18.5–24.9 kg/m^2^), and overweight (> 25.0 kg/m^2^) categories (NHLBI Obesity Education Initiative Expert Panel on the Identification Evaluation and Treatment of Obesity in Adults (US) 1998).

It has been proposed that the distribution of body fat is an important indicator of increased risk of CVD (He et al. 2018). In the NDNS, nurses measured the waist and hip circumstance and such data have been used for our calculation of whgval (both subcutaneous and intra-abdominal) in the present study. The whgval has been classified as normal weight, overweight and obesity, using the cut-off points as < 0.8 for women and < 0.9 for men, 0.8–0.84 for women and 0.9–0.99 for men and > 0.85 for women and > 1 for men according to DGSP regulation (Dt. Gesellschaft für Sportmedizin und Prävention e.V. (DGSP) 2007).

In addition, confounding information such as SalHowC, MN and EnergyDkJ, ProteingD, FatgD, GlucosegD, SodiummgD as well as FolateugplussuppsD was all derived from the food diary. Among these, EnergyDkJ, ProteingD, FatgD, GlucosegD, SodiummgD and FolateugplussuppsD were divided into four quartiles based on their distribution in the population. For MN, a score of 0 or 1 was assigned to participants with less than (<) or greater than or equal to (≥) the mean daily intake level of each nutrient, respectively, with a composite measure then created by summing the individual score to indicate the intake level of these micro-nutrients (cf. El-Masri et al. 2018).

Moreover, drinking water and grain and grain-based products have been regarded as two important exposure pathways for iAs in the UK and some other European countries (European Food Safety Authority 2014), and this study, therefore, included E-iAs_ing,water_ (µg/person/day) and E-iAs_ing,grain_ (µg/person/day) as confounders with their calculation following Eqs. (3), (4) for E-iAs_ing,water_ and Eqs. (5), (6) for E-iAs_ing,grain_:

3 $${\text{E-iAs}}_{\text{ing,water}} = C_{\text{water}} \times {\text{WC}}$$

4 $${\text{WC}} = \frac{{\mathop \sum \nolimits_{ 1}^{n} {\text{DWC}}}}{n}$$

5 $${\text{E-iAs}}_{\text{ing,grain}} = \mathop \sum \limits_{i} C_{{{\text{grain,}}i}} \times {\text{GC}}_{i}$$

6 $${\text{GC}}_{i} = \frac{{\mathop \sum \nolimits_{1}^{n} {\text{DGC}}_{i} }}{n}$$

where *C*_water_: iAs concentration (µg/L) in drinking water, WC: average daily intake (L/day) of drinking water during the food diary, *n*: length of the food diary [3 days (*n *= 3) or 4 days (*n *= 4), DWC: consumption (L/day) of drinking water in each day during the food diar, *C*_grain,*i*_: iAs concentration (µg/kg) of the grain and grain-based product, *i*, GC_*i*_: average daily consumption (kg/day) of grain and grain-based product, *i*, during the food diary, DGC_*i*_: consumption rate (kg/day) of grain and grain-based product, *i*, in each day during the food diary, Similar to E-iAs_ing,rice_, consumption rates for drinking water and grain and grain-based products were calculated for all food diary periods, and the average daily consumption values were used for average consumption level of each survey participant. Values for C_grain,*i*_ and C_water_ were estimated from those reported by European Food Safety Authority (2014).

### Statistical analysis

In this study, we explored, for the UK population from April 2014 through August 2015 and April 2015 through August 2016, the extent to which E-iAs_ing,rice_ modifies the association between hypertension risks (several blood pressure endpoints, including DBP add 10, SBP add 10, AP, meanPulse and general hypertension) and previously well-established risk factors categorically and continuously, utilizing a series of generalized linear models. In addition, the influence of an appropriate range of socio-economic, demographic and lifestyle as confounders is explored through minimizing objective model comparison criteria, notably Akaike’s Information Criterion (AIC) (Bozdogan 1987).

Statistical analysis was conducted through R statistical software [version 3.4.3 (R Foundation for Statistical Computing)].

Before the main analysis, we compared participants’ [over 16 (*N* = 1649)] iAs intake level and some demographic and lifestyle characteristics for those included and those excluded from the present study (see previous exclusion criteria), with significance of differences being computed by Fisher’s exact test, Mann–Whitney U Test and Student’s *t* test as appropriate. Participants included and participants excluded from this study were found to have similar levels of E-iAs_ing,rice_, NumChild, gender and ethnicity distribution, health condition, bmival and whgval. However, participants were more likely to be middle-aged, in employment, have higher household income or live in England compared to other UK countries (Table S2).

For the main analysis, E-iAs_ing,rice_ was either categorized in quartiles based on its weighted distributions in the study population or was used as a continuous measure.

Descriptive analysis was firstly conducted comparing participants by E-iAs_ing,rice_ quartiles and by the status of general hypertension in terms of socio-demographic and lifestyle characteristics and some established or suspected risk factors of hypertension. The results were reported as means [standard deviations (SD)] for continuous variables (E-iAs_ing,rice_, E-iAs_ing,water_ and E-iAs_ing,grain_) or as frequencies (percentages  %) for categorical ones (surveyyr, Quarter, Sex, age, ethgrp5, qual7, cigsta3 and eqv3, dnoft, HessCon, NumChild, Diabetes.combined, WrkStat, MN, EnergyDkJ, ProteingD, FatgD, GlucosegD, SodiummgD, FolateugplussuppsD, bmival, region, SalHowC and whgval). The significance of differences across intake quartiles or general hypertension status was determined from *χ*^2^ tests and Wilcoxon rank-sum tests with Tukey post hoc tests, and the *p* values for trend were computed via analysis of variance (ANOVA) test with type II error.

Then, we quantified the individual and interactive contributions of E-iAs_ing,rice_ and all the potential confounders to the variability of hypertension risks (DBP add 10, SBP add 10, AP, meanPulse and general hypertension, respectively) through generalized linear model (GLM) (contributions (%) = 100 × (null deviance–residual deviance)/null deviance) (Bjorndal et al. 2013). Among this, the importance of two-way interactive effects was calculated based on the relative excess risk for interaction (RERI) according to Chen et al. (Chen et al. 2011) [see Eq. (7)], with a value over zero indicating the presence of synergy effects:

7 $${\text{RERI}} = {\text{e}}^{{\left( {\beta 1 + \beta 2 + \beta 3} \right)}} - {\text{e}}^{\beta 1} - {\text{e}}^{\beta 2} + 1.$$

where *β*1: the continuous coefficient of E-iAs_ing,rice_, *β*2: the coefficient of each potential confounder, *β*3: the interactive term coefficient.

The resultant *p* values were computed using ANOVA test with type II error with the values for interaction obtained by adding a cross-product term between continuous iAs intake and different confounders to the main model.

We finally analysed the extent to which E-iAs_ing,rice_ modifies the association between hypertension risks (DBP add 10, SBP add 10, AP, meanPulse and general hypertension) and previously well-established risk factors by GLMs. Separate independent models were used for the changes of DBP add 10, SBP add 10, AP, meanPulse and the odds ratio of general hypertension to assess their associations with E-iAs_ing,rice_, respectively.

To firstly get a general understanding of whether E-iAs_ing,rice_ could significantly modify the association between hypertension risks and previously well-established risk factors, we obtained a best-fitted model of hypertension risks without E-iAs_ing,rice_ but including some important previously known risks factors and then tested whether or not adding this intake factor as a confounder made that model better.

To further evaluate the associations between E-iAs_ing,rice_ and hypertension risks at various intake levels, both categorical and continuous intake variables were used in the crude and multivariable adjusted models. For categorical analysis, the odds ratios for general hypertension (binary variable) and the corresponding 95% confidence intervals (CIs) were calculated by using logistic regression to compare participants in quartiles of E-iAs_ing,rice_. Similarly, SBP add 10, DBP add 10, AP and meanPulse (continuous variables) and the corresponding 95% CIs for different intake quartiles were estimated and compared between each of the higher 3 quartiles with the bottom one by multiple linear regression models. For continuous analysis, E-iAs_ing,rice_ was used as a continuous measure to evaluate the changes of each blood pressure endpoint for an increase of 1 µg/person/day E-iAs_ing,rice_, respectively. In addition, we examined the assumption of nonlinear relationships by including higher order polynomial terms for E-iAs_ing,rice_ in the best-fitted linear models. To allow a more flexible dose–response association, we also estimated DBP add 10, SBP add 10, AP, meanPulse and the odds ratio of general hypertension by dividing E-iAs_ing,rice_ into 15 groups based on its distributions in the study population. The differences of the hypertension risks across four quartiles or 15 groups of iAs intake were obtained from Wald tests for E-iAs_ing,rice_ coefficients, and the *p* values for linear and nonlinear trends were computed by ANOVA test with type II error where E-iAs_ing,rice_ is a continuous measure.

The main covariate of interest was determined a priori based on biological and behavioural plausibility (such as Sex, age, ethgrp5, cigsta3 and dnoft, HessCon, Diabetes.combined, MN, SalHowC, EnergyDkJ, ProteingD, FatgD, GlucosegD, SodiummgD, FolateugplussuppsD, bmival and whgval), and some socio-economic information (surveyyr, Quarter, region, qual7, eqv3, NumChild, WrkStat) as well as other important exposure pathways (E-iAs_ing,water_ or E-iAs_ing,grain_) if they had a *p* value less than 0.2 in the univariate models. For better modelling their relationships, we also checked the existence of multi-collinearity problems. Given the sample size, the number of variables included (Burnham and Anderson 2002) as well as the best and most parsimonious fit, models were derived from the full set of data and reduced by model selection using AIC with the stepwise selection allowed in both directions.

Vulnerability to As toxicity differs widely from person to person, and hypertension risks may be higher in certain susceptible subgroups. However, most of such variability in susceptibility to date remains unexplained (Steinmaus et al. 2015). To identify the most vulnerable subgroups and provide better suggestions for lowering the adverse effects of iAs intake, we performed subgroup analysis to evaluate effect modifications in adjusted models for subgroups defined by Sex, age, ethgrp5, bmival, cigsta3, dnoft, Diabetes.combined and region. Forest plots have been applied to show the changes of hypertension risks for an increase of 1 µg/person/day E-iAs_ing,rice_ by participants’ characteristics. In the forest plot, boxes represent the SBP add 10, DBP add 10, AP, meanPulse and the odds ratio of general hypertension, with horizontal lines indicating their 95% CIs. *p* values for the interaction were obtained by adding a cross-product term between E-iAs_ing,rice_ and the corresponding characteristic in the multivariable model, computed by the ANOVA test with type II error to account for the complex design.

For the assessment of the consistency of the findings, a sensitivity analysis has also been conducted. Specifically, we excluded people taking anti-hypertension medications, identifying whether the estimated associations in the main analysis would be substantially different after such exclusion.

## Results

### Characteristics of E-iAs_ing,rice_, hypertension risks and all the potential confounders

In this study, the mean values of SBP add 10 and DBP add 10 (127 (SD = 19) and 75 (SD = 12) mmHg) were lower than the minimum values defining general hypertension (140 and 90 mmHg, respectively) with about 30% participants being classified as having general hypertension. In addition, the mean level of AP and meanPulse was 92 (SD = 14) and 69 (SD = 11) mmHg, respectively.

For the E-iAs_ing,rice_, ranging from 0 to 41.8 µg/person/day, participants included in this study consumed a mean level of 2.81 µg iAs each day with its SD being 4.73 µg/person/day.

There were significant associations between E-iAs_ing,rice_, hypertension risks and some of the potential confounders (Tables 2, 3). Participants who have general hypertension tended to be older, overweight, less healthy, have less children or have become diabetics. Also, a larger proportion of participants who have no jobs or were current or ex-regular cigarette smokers could be found in the hypertension group. In addition, there were significant associations between E-iAs_ing,rice_ and Sex, age, ethgrp5, nutrient intake, qual7 and E-iAs_ing,water_ with people taking higher level iAs from rice and rice products being generally younger, not White or having a higher education level.

**Table 2:** Characteristics of population satisfying inclusion criteria by hypertension status in the NDNS RP 7–8. Data from NDNS RP 7–8 (MRC Elsie Widdowson Laboratory and NatCen Social Research 2019) with exclusions as detailed in the text (*N* = 598)

Variable	General hypertension		
	Yes (*N* = 178)	No (*N* = 420)	*p* value*
E-iAs_ing,rice_ (µg/person/day)	2.17 (3.65)	3.08 (5.11)	0.015
E-iAs_ing,water_ (µg/person/day)	0.74 (0.70)	1.02 (1.10)	<0.001
E-iAs_ing,grain_ (µg/person/day)	2.69 (1.32)	2.89 (1.47)	0.100
*Sex*			
Male	80 (44.94)	171 (40.71)	0.365
Female	98 (55.06)	249 (59.29)	
*surveyyr*			
2014/15	90 (50.56)	203 (48.33)	0.655
2015/16	88 (49.44)	217 (51.67)	
*HessCon*			
With any physical/mental health condition/illnesses for 12 months or more	98 (55.06)	119 (28.33)	< 0.001
Without any physical/mental health condition/illnesses for 12 months or more	80 (44.94)	301 (71.67)	
*Diabetes.combined*			
Without diabetes	149 (83.71)	402 (95.71)	< 0.001
Diabetic	29 (16.29)	18 (4.29)	
*NumChild*			
Have no child	152 (85.39)	259 (61.67)	< 0.001
Have 1–2 child	23 (12.92)	143 (34.05)	
Have 3–4 child	3 (1.69)	18 (4.29)	
*Age*			
16–34 years	4 (2.25)	151 (35.95)	< 0.001
35–49 years	32 (17.98)	130 (30.95)	
50–64 years	71 (39.89)	91 (21.67)	
65 + year	71 (39.89)	48 (11.43)	
*Quarter*			
Season 1	29 (16.29)	98 (23.33)	0.217
Season 2	45 (25.28)	106 (25.24)	
Season 3	52 (29.21)	101 (24.05)	
Season 4	52 (29.21)	115 (27.38)	
*WrkStat*			
In full- or part-time employment	75 (42.13)	259 (61.67)	< 0.001
Full-time student or not working	103 (57.87)	161 (38.33)	
*ethgrp5*			
White	167 (93.82)	383 (91.19)	0.641
Mixed	1 (0.56)	7 (1.67)	
Black or Black British	2 (1.12)	12 (2.86)	
Asian or Asian British	6 (3.37)	14 (3.33)	
Any other groups	2 (1.12)	4 (0.95)	
*eqv3* (£)			
Lowest tertile (≤ 17,500)	53 (29.78)	94 (22.38)	0.162
Middle tertile (> 17,500 and ≤ 32,216)	53 (29.78)	135 (32.14)	
Highest tertile (> 32,500)	72 (40.45)	191 (45.48)	
*cigsta3*			
Current cigarette smoker	19 (10.67)	66 (15.71)	< 0.001
Ex-regular cigarette smoker	62 (34.83)	80 (19.05)	
Never regular cigarette smoker	97 (54.49)	274 (65.24)	
*dnoft*			
5–7 days per week	27 (15.17)	32 (7.62)	0.123
3–4 days per week	24 (13.48)	48 (11.43)	
1–2 days per week	44 (24.72)	125 (29.76)	
1–2 per month	27 (15.17)	71 (16.90)	
Once every couples of months	14 (7.87)	47 (11.19)	
1–2 per year	18 (10.11)	45 (10.71)	
Not at all in the last 12 months/non-drinker	24 (13.48)	52 (12.38)	
*bmival* (kg/m^2^)			
Underweight: BMI < 18.5	0 (0.00)	14 (3.33)	< 0.001
Normal (healthy weight): BMI between 18.5 and 25	37 (20.79)	172 (40.95)	
Overweight: BMI between 25 and 30	74 (41.57)	153 (36.43)	
Obese: BMI over 30	67 (37.64)	81 (19.29)	
*whgval*			
For male: less than 0.9; For female: less than 0.80	28 (15.73)	207 (49.29)	< 0.001
For male: more than and including 0.90, up to and including 1.00; for female: more than and including 0.80, up to and including 0.85	54 (30.34)	113 (26.90)	
For male: more than 1.00; for female: more than 0.85	96 (53.93)	100 (23.81)	
*MN*			
Participants with 0–3 nutrients greater than or equal to the mean intake level	34 (19.10)	100 (23.81)	0.736
Participants with 4–7 nutrients greater than or equal to the mean intake level	36 (20.22)	81 (19.29)	
Participants with 8–11 nutrients greater than or equal to the mean intake level	42 (23.60)	101 (24.05)	
Participants with 12–15 nutrients greater than or equal to the mean intake level	40 (22.47)	84 (20.00)	
Participants with 16–18 nutrients greater than or equal to the mean intake level	26 (14.61)	54 (12.86)	
*EnergyDkJ* (KJ)			
Q1: 189.46–1294.86	27 (15.17)	52 (12.38)	0.673
Q2: 1295.07–1627.87	33 (18.54)	86 (20.48)	
Q3: 1628.14–2037.01	52 (29.21)	113 (26.90)	
Q4: 2037.83–4771.28	66 (37.08)	169 (40.24)	
*ProteingD* (g)			
Q1: 1.17–11.90	21 (11.80)	43 (10.24)	0.338
Q2: 11.91–15.42	31 (17.42)	91 (21.67)	
Q3: 15.43–19.31	56 (31.46)	107 (25.48)	
Q4: 19.33–69.00	70 (39.33)	179 (42.62)	
*FatgD* (g)			
Q1: 0.12–10.64	34 (19.10)	59 (14.05)	0.466
Q2: 10.65–14.21	31 (17.42)	83 (19.76)	
Q3: 14.23–18.71	46 (25.84)	110 (26.19)	
Q4: 18.74–59.89	67 (37.64)	168 (40.00)	
*GlucosegD* (g)			
Q1: 0.06–2.19	41 (23.03)	75 (17.86)	0.543
Q2: 2.20–3.34	34 (19.10)	88 (20.95)	
Q3: 3.35–4.88	45 (25.28)	115 (27.38)	
Q4: 4.89–30.28	58 (32.58)	142 (33.81)	
*SodiummgD* (mg)			
Q1: 22.97–327.89	29 (16.29)	62 (14.76)	0.617
Q2: 327.91–430.23	33 (18.54)	86 (20.48)	
Q3: 430.26–561.96	56 (31.46)	114 (27.14)	
Q4: 562.28–2306.84	60 (33.71)	158 (37.62)	
*FolateugplussuppsD* (µg)			
Q1: 5.69–35.36	20 (11.24)	54 (12.86)	0.614
Q2: 36.38–47.85	30 (16.85)	87 (20.71)	
Q3: 47.87–65.81	52 (29.21)	109 (25.95)	
Q4: 65.86–1426.32	76 (42.70)	170 (40.48)	
*qual7*			
Degree or equivalent	47 (26.4)	148 (35.24)	0.084
Higher education, below degree level; GCE, a level or equivalent	35 (19.66)	85 (20.24)	
GCSE grades A–G or equivalent/commercial qualifications/apprenticeship	33 (18.54)	77 (18.33)	
Foreign or other qualifications; no qualifications; still in FT education	63 (35.39)	110 (26.19)	
*Region*			
England Central/Midlands	27 (15.17)	55 (13.1)	0.395
England North	40 (22.47)	99 (23.57)	
England South (including London)	58 (32.58)	162 (38.57)	
Northern Ireland and Scotland	12 (6.74)	32 (7.62)	
Wales	41 (23.03)	72 (17.14)	
*SalHowC*			
Never	85 (47.75)	209 (49.76)	0.867
Sometimes and usually	43 (24.16)	101 (24.05)	
Always	50 (28.09)	110 (26.19)	

The results were reported as means (standard deviations (SD)) for continuous variables (E-iAs_ing,rice_, E-iAs_ing,water_ and E-iAs_ing,grain_) or as frequencies (percentages (%)) for categorical ones (surveyyr, Quarter, Sex, age, ethgrp5, qual7, cigsta3 and eqv3, dnoft, HessCon, NumChild, Diabetes.combined, WrkStat, MN, EnergyDkJ, ProteingD, FatgD, GlucosegD, SodiummgD, FolateugplussuppsD, bmival, region, SalHowC and whgval)

Differences were computed by hypertension status via χ^2^ tests and Wilcoxon rank-sum tests

General hypertension: whether participants were diagnosed as general hypertension; E-iAs_ing,rice_: daily inorganic arsenic (iAs) intake from rice and rice products; E-iAs_ing,water_: daily iAs intake from drinking water; E-iAs_ing,grain_: daily iAs intake from grain and grain-based products; surveyyr: NDNS RP 7–8 survey year; Sex: gender; EnergyDkJ: intake of total energy per day (KJ) for diet only; ProteingD: intake of protein per day (g) for diet only; FatgD: intake of fat per day (g) for diet only; GlucosegD: intake of glucose per day (g) for diet only; SodiummgD: intake of sodium per day (mg) for diet only; FolateugplussuppsD: intake of folate (µg) per day for both diets and supplements; MN: daily intake of several micro-nutrients (Potassium (mg) including supplements, calcium (mg) including supplements, magnesium (mg) including supplements, iron (mg) including supplements, copper (mg) including supplements, zinc (mg) including supplements, retinol (mg) including supplements, vitamin A (retinol equivalents) (µg) including supplements, vitamin D (µg) including supplements, vitamin E (mg) including supplements, thiamin (mg) including supplements, riboflavin (mg) including supplements, niacin equivalent (mg) including supplements, vitamin B6 (mg) including supplements, vitamin B12 (µg) including supplements, vitamin C (mg) including supplements, iodine (µg) including supplements, selenium (µg) including supplements); region: country people live; NumChild: number of children aged between 0 and 15; age: age of respondent 16 + year old; SalHowC: how often salt added during cooking; Quarter: fieldwork quarter; qual7: qualifications gained; WrkStat: economic status (working condition); ethgrp5: ethnic group; eqv3: equivalized household income; HessCon: whether have any physical/mental health condition/illnesses for 12 months or more; Diabetes.combined: whether respondent is diabetic; cigsta3: cigarette smoking status; dnoft: frequency of alcohol consumption in past 12 months (including non-drinkers); bmival: BMI (kg/m^2^); whgval: waist-hip ratio groups

*Indicating whether there is statistically significant relationship between characteristics of population and status of general hypertension

**Table 3:** Characteristics of population satisfying inclusion criteria by the distribution of E-iAs_ing,rice_ in the NDNS RP 7–8. Data from NDNS RP 7–8 (MRC Elsie Widdowson Laboratory and NatCen Social Research 2019) with exclusions as detailed in the text (*N* = 598)

Characteristic	Quartile of E-iAs_ing,rice_, µg/person/day							*p* value for trend
	Quartile 1 (0.00–0.00) *N* = 150	Quartile 2 (0.00–0.565) *N* = 149	*p* value*	Quartile 3 (0.638–3.79) *N* = 149	*p* value*	Quartile 4 (3.79–41.8) *N* = 150	*p* value^*^	
E-iAs_ing,grain_ (µg/person/day)	2.98 (1.42)	2.90 (1.48)	0.966	2.69 (1.38)	0.304	2.77 (1.43)	0.572	0.235
E-iAs_ing,water_ (µg/person/day)	0.72 (0.76)	0.72 (0.70)	0.871	1.11 (1.22)	0.004	1.21 (1.15)	< 0.001	< 0.001
*Sex*								
Female	86 (57.33)	93 (62.42)	0.436	93 (62.42)	0.436	75 (50.00)	0.247	0.006
Male	64 (42.67)	56 (37.58)		56 (37.58)		75 (50.00)		
*surveyyr*								
2014/15	132 (88.00)	10 (6.71)	< 0.001	75 (50.34)	< 0.001	76 (50.67)	< 0.001	0.969
2015/16	18 (12.00)	139 (93.29)		74 (49.66)		74 (49.33)		
*HessCon*								
Without any physical/mental health condition/illnesses for 12 months or more	84 (56.00)	100 (67.11)	0.063	102 (68.46)	0.036	95 (63.33)	0.290	0.461
With any physical/mental health condition/illnesses for 12 months or more	66 (44.00)	49 (32.89)		47 (31.54)		55 (36.67)		
*Diabetes combined*								
Without diabetes	139 (92.67)	139 (93.29)	0.733	135 (90.60)	0.663	138 (92.00)	0.819	0.576
Diabetic	11 (7.33)	10 (6.71)		14 (9.40)		12 (8.00)		
*NumChild*								
Have no child	113 (75.33)	100 (67.11)	0.044	97 (65.10)	0.154	101 (67.33)	0.291	0.900
Have 1–2 child	31 (20.67)	47 (31.54)		45 (30.20)		43 (28.67)		
Have 3–4 child	6 (4.00)	2 (1.34)		7 (4.70)		6 (4.00)		
*Age*								
16–34	32 (21.33)	35 (23.49)	0.354	45 (30.20)	0.032	43 (28.67)	0.003	0.005
35–49	30 (20.00)	41 (27.52)		41 (27.52)		50 (33.33)		
50–64	48 (32.00)	39 (26.17)		39 (26.17)		36 (24.00)		
65+	40 (26.67)	34 (22.82)		24 (16.11)		21 (14.00)		
*Quarter*								
Season 1	30 (20.00)	32 (21.48)	0.695	28 (18.79)	0.543	37 (24.67)	0.788	0.992
Season 2	39 (26.00)	43 (28.86)		30 (20.13)		39 (26.00)		
Season 3	40 (26.67)	36 (24.16)		41 (27.52)		36 (24.00)		
Season 4	41 (27.33)	38 (25.50)		50 (33.56)		38 (25.33)		
*WrkStat*								
In full- or part-time employment	77 (51.33)	79 (53.02)	0.860	87 (58.39)	0.267	91 (60.67)	0.131	0.053
Full-time student or not working	73 (48.67)	70 (46.98)		62 (41.61)		59 (39.33)		
*ethgrp5*								
Any other group	1 (0.67)	2 (1.34)	0.548	0 (0.00)	0.232	3 (2.00)	< 0.001	< 0.001
Asian or Asian British	3 (2.00)	3 (2.01)		0 (0.00)		14 (9.33)		
Black or Black British	1 (0.67)	2 (1.34)		1 (0.67)		10 (6.67)		
Mixed ethnic group	0 (0.00)	3 (2.01)		1 (0.67)		4 (2.67)		
White	145 (96.67)	139 (93.29)		147 (98.66)		119 (79.33)		
*eqv3* (£)								
Lowest tertile (≤ 17,500)	40 (26.67)	38 (25.50)	0.873	32 (21.48)	0.023	37 (24.67)	0.641	0.787
Middle tertile (> 17,500 and ≤ 32,216)	53 (35.33)	50 (33.56)		37 (24.83)		48 (32.00)		
Highest tertile (> 32,500)	57 (38.00)	61 (40.94)		80 (53.69)		65 (43.33)		
*cigsta3*								
Current cigarette smoker	23 (15.33)	20 (13.42)	0.892	23 (15.44)	0.475	19 (12.67)	0.297	0.182
Ex-regular cigarette smoker	40 (26.67)	40 (26.85)		31 (20.81)		31 (20.67)		
Never regular cigarette smoker	87 (58.00)	89 (59.73)		95 (63.76)		100 (66.67)		
*dnoft*								
Not at all in the last 12 months/non-drinker	24 (16.00)	14 (9.40)	0.211	21 (14.09)	0.599	17 (11.33)	0.225	0.749
1–2 per year	19 (12.67)	20 (13.42)		11 (7.38)		13 (8.67)		
1–2 per month	13 (8.67)	21 (14.09)		12 (8.05)		15 (10.00)		
Once every couples of months	20 (13.33)	26 (17.45)		27 (18.12)		25 (16.67)		
1–2 days per week	43 (28.67)	33 (22.15)		42 (28.19)		51 (34.00)		
3–4 days per week	13 (8.67)	20 (13.42)		19 (12.75)		20 (13.33)		
5–7 days per week	18 (12.00)	15 (10.07)		17 (11.41)		9 (6.00)		
*bmival* (kg/m^2^)								
Underweight: under 18.5	6 (4.00)	4 (2.68)	0.117	2 (1.34)	0.214	2 (1.33)	0.179	0.872
Normal (healthy weight): 18.5 and below 25	42 (28.00)	59 (39.60)		52 (34.90)		56 (37.33)		
Overweight: 25 and below 30	60 (40.00)	54 (36.24)		63 (42.28)		50 (33.33)		
Obese: over 30	42 (28.00)	32 (21.48)		32 (21.48)		42 (28.00)		
*whgval*								
For male: less than 0.9; For female: less than 0.80	50 (33.33)	61 (40.94)	0.354	59 (39.60)	0.123	65 (43.33)	0.204	0.321
For male: more than and including 0.90, up to and including 1.00; For female: more than and including 0.80, up to and including 0.85	42 (28.00)	40 (26.85)		49 (32.89)		36 (24.00)		
For male: more than 1.00; for female: more than 0.85	58 (38.67)	48 (32.21)		41 (27.52)		49 (32.67)		
*MN*								
Participants with 0–3 nutrients greater than or equal to the mean intake level	38 (25.33)	34 (22.82)	0.678	36 (24.16)	0.881	26 (17.33)	0.329	0.007
Participants with 4–7 nutrient 4 greater than or equal to the mean intake level	25 (16.67)	32 (21.48)		30 (20.13)		30 (20.00)		
Participants with 8–11 nutrients greater than or equal to the mean intake level	36 (24.00)	41 (27.52)		33 (22.15)		33 (22.00)		
Participants with 12–15 nutrients greater than or equal to the mean intake level	29 (19.33)	23 (15.44)		32 (21.48)		40 (26.67)		
Participants with 16–18 nutrients greater than or equal to the mean intake level	22 (14.67)	19 (12.75)		18 (12.08)		21 (14.00)		
*EnergyDkJ* (KJ)								
Q1: 189.46–1294.86	28 (18.67)	18 (12.08)	0.221	21 (14.09)	0.432	12 (8.00)	0.012	0.013
Q2: 1295.07–1627.87	33 (22.00)	32 (21.48)		30 (20.13)		24 (16.00)		
Q3: 1628.14–2037.01	33 (22.00)	46 (30.87)		44 (29.53)		42 (28.00)		
Q4: 2037.83–4771.28	56 (37.33)	53 (35.57)		54 (36.24)		72 (48.00)		
*ProteingD* (g)								
Q1: 1.17–11.90	17 (11.33)	20 (13.42)	0.439	17 (11.41)	0.933	10 (6.67)	0.044	0.045
Q2: 11.91–15.42	38 (25.33)	27 (18.12)		34 (22.82)		23 (15.33)		
Q3: 15.43–19.31	37 (24.67)	44 (29.53)		41 (27.52)		41 (27.33)		
Q4: 19.33–69.00	58 (38.67)	58 (38.93)		57 (38.26)		76 (50.67)		
*FatgD* (g)								
Q1: 0.12–10.64	22 (14.67)	19 (12.75)	0.034	29 (19.46)	0.591	23 (15.33)	0.127	0.592
Q2: 10.65–14.21	36 (24.00)	21 (14.09)		36 (24.16)		21 (14.00)		
Q3: 14.23–18.71	31 (20.67)	50 (33.56)		33 (22.15)		42 (28.00)		
Q4: 18.74–59.89	61 (40.67)	59 (39.60)		51 (34.23)		64 (42.67)		
*GlucosegD* (g)								
Q1: 0.06–2.19	34 (22.67)	34 (22.82)	0.986	26 (17.45)	0.452	22 (14.67)	0.302	0.269
Q2: 2.20–3.34	29 (19.33)	29 (19.46)		31 (20.81)		33 (22.00)		
Q3: 3.35–4.88	35 (23.33)	37 (24.83)		45 (30.20)		43 (28.67)		
Q4: 4.89–30.28	52 (34.67)	49 (32.89)		47 (31.54)		52 (34.67)		
*SodiummgD* (mg)								
Q1: 22.97–327.89	24 (16.00)	22 (14.77)	0.503	23 (15.44)	0.737	22 (14.67)	0.216	0.317
Q2: 327.91–430.23	36 (24.00)	27 (18.12)		29 (19.46)		27 (18.00)		
Q3: 430.26–561.96	44 (29.33)	44 (29.53)		44 (29.53)		38 (25.33)		
Q4: 562.28–2306.84	46 (30.67)	56 (37.58)		53 (35.57)		63 (42.00)		
*FolateugplussuppsD *(µg)								
Q1: 5.69–35.36	17 (11.33)	20 (13.42)	0.770	22 (14.77)	0.444	15 (10.00)	0.049	0.313
Q2: 36.38–47.85	32 (21.33)	36 (24.16)		23 (15.44)		26 (17.33)		
Q3: 47.87–65.81	33 (22.00)	34 (22.82)		39 (26.17)		55 (36.67)		
Q4: 65.86–1426.32	68 (45.33)	59 (39.60)		65 (43.62)		54 (36.00)		
*qual7*								
Foreign or other qualifications; No qualifications; Still in FT education	48 (32.00)	47 (31.54)	0.701	42 (28.19)	0.901	36 (24.00)	0.266	0.025
GCSE grades A–G or equivalent/commercial qualifications/apprenticeship	27 (18.00)	33 (22.15)		28 (18.79)		22 (14.67)		
Higher education, below degree level; GCE, A level or equivalent	28 (18.67)	30 (20.13)		28 (18.79)		34 (22.67)		
Degree or equivalent	47 (31.33)	39 (26.17)		51 (34.23)		58 (38.67)		
*Region*								
England Central/Midlands	25 (16.67)	21 (14.09)	0.618	16 (10.74)	0.099	20 (13.33)	0.442	0.155
England North	40 (26.67)	36 (24.16)		27 (18.12)		36 (24.00)		
England South (including London)	46 (30.67)	56 (37.58)		59 (39.60)		59 (39.33)		
Northern Ireland and Scotland	8 (5.33)	11 (7.38)		14 (9.40)		11 (7.33)		
Wales	31 (20.67)	25 (16.78)		33 (22.15)		24 (16.00)		
*SalHowC*								
Never	76 (50.67)	68 (45.64)	0.230	86 (57.72)	0.431	64 (42.67)	0.381	0.088
Sometimes and usually	38 (25.33)	32 (21.48)		30 (20.13)		44 (29.33)		
Always	36 (24.00)	49 (32.89)		33 (22.15)		42 (28.00)		

The results were reported as means (standard deviations (SD)) for continuous variables (E-iAs_ing,water_ and E-iAs_ing,grain_) or as frequencies (percentages (%)) for categorical ones (surveyyr, quarter, sex, age, ethgrp5, qual7, cigsta3 and eqv3, dnoft, HessCon, NumChild, Diabetes.combined, WrkStat, MN, EnergyDkJ, ProteingD, FatgD, GlucosegD, SodiummgD, FolateugplussuppsD, bmival, region, SalHowC and whgval)

Differences across quartiles were computed by exposure categories via χ^2^ tests or Wilcoxon rank-sum tests with Tukey post hoc test. *p* values for trend were obtained from analysis of variance (ANOVA) test with type II error where the characteristic factors were treated as continuous variables, indicating whether or not there is a statistically significant relationship between those characteristics of the population and iAs intake

E-iAs_ing,rice_: daily inorganic arsenic (iAs) intake from rice and rice products; E-iAs_ing,water_: daily iAs intake from drinking water; E-iAs_ing,grain_: daily iAs intake from grain and grain-based products; surveyyr: NDNS RP 7–8 Survey year; Sex: gender; EnergyDkJ: intake of total energy per day (KJ) for diet only; ProteingD: intake of protein per day (g) for diet only; FatgD: intake of fat per day (g) for diet only; GlucosegD: intake of glucose per day (g) for diet only; SodiummgD: intake of sodium per day (mg) for diet only; FolateugplussuppsD: intake of folate (µg) per day for both diets and supplements; MN: daily intake of several micro-nutrients (potassium (mg) including supplements, calcium (mg) including supplements, magnesium (mg) including supplements, iron (mg) including supplements, copper (mg) including supplements, zinc (mg) including supplements, retinol (mg) including supplements, vitamin A (retinol equivalents) (µg) including supplements, vitamin D (µg) including supplements, vitamin E (mg) including supplements, thiamin (mg) including supplements, riboflavin (mg) including supplements, niacin equivalent (mg) including supplements, vitamin B6 (mg) including supplements, vitamin B12 (µg) including supplements, vitamin C (mg) including supplements, iodine (µg) including supplements, selenium (µg) including supplements); region: country people live; NumChild: number of children aged between 0 and 15; age: age of respondent 16 + ; SalHowC: how often salt added during cooking; Quarter: fieldwork quarter; qual7: qualifications gained; WrkStat: Economic status (working condition); ethgrp5: ethnic group; eqv3: equivalized household income; HessCon: whether have any physical/mental health condition/illnesses for 12 months or more; Diabetes.combined: whether respondent is diabetic; cigsta3: cigarette smoking status; dnoft: frequency of alcohol consumption in past 12 months (including non-drinkers); bmival: BMI (kg/m^2^); whgval: waist-hip ratio groups

*Compared with Quartile 1 (referent group) (0.00–0.00 µg/person/day)

### Importance of different factors to the hypertension risks

Whatever the relationship between E-iAs_ing,rice_ and hypertension risks, it is likely to be of lesser importance than a number of factors that are widely known to be important indicators, such as age, obesity, gender, smoking status, alcohol consumption and sodium intake (Biino et al. 2013; He et al. 2018; NHLBI Obesity Education Initiative Expert Panel on the Identification Evaluation and Treatment of Obesity in Adults (US) 1998). Thus, the relationship between E-iAs_ing,rice_ and hypertension risks can only be reasonably determined after first quantifying the importance of these factors.

To quantify the contributions of factors shown to influence hypertension risks and attempt to account for their importance, GLM was performed in our analysis. According to Table 4, similar results could be observed for SBP add 10, DBP add 10, AP and general hypertension. Specifically, age was the most important contributor for the variability of those blood pressure endpoints, accounting for over 20% of the observed variations. Meanwhile, whgval and bmival, though not the most standing out, also significantly contributed to more than 10%. Such phenomenon could be supported by previous researches, which indicated that variables explained most of the hypertension risks were age and BMI (Biino et al. 2013; Lelong et al. 2019) with about 11–17% of the hypertension risks due to overweight (Geleijnse et al. 2005). However, cigsta3, E-iAs_ing,water_, HessCon, NumChild were all significantly associated with the hypertension risks, but only explained a small percentage of those risks. In addition, due to its low level, E-iAs_ing,rice_ alone did not play an important role in those blood pressure endpoints, contributing even less than 1%.

**Table 4:** Individual contributions of different factors to the variability of hypertension risks). Data from NDNS RP 7–8 (MRC Elsie Widdowson Laboratory and NatCen Social Research 2019) with population satisfying inclusion criteria as detailed in the text (*N* = 598

	DBP add 10		SBP add 10		AP		meanPulse		General hypertension	
	Contribution (%)	*p* value*	Contribution (%)	*p* value*	Contribution (%)	*p* value*	Contribution (%)	*p* value*	Contribution (%)	*p* value*
E-iAs_ing,rice_	0.70	0.040	0.93	0.018	0.92	0.019	0.03	0.661	0.71	0.023
E-iAs_ing,water_	0.95	0.017	1.63	0.002	1.41	0.004	0.48	0.090	1.60	0.001
E-iAs_ing,grain_	0.72	0.037	0.07	0.514	0.41	0.118	0.93	0.018	0.35	0.110
Sex	0.27	0.201	2.11	< 0.001	0.99	0.014	2.15	0.003	0.13	0.339
surveyyr	0.00	0.970	0.32	0.166	0.07	0.533	0.04	0.641	0.03	0.618
HessCon	2.58	< 0.001	4.73	< 0.001	3.96	< 0.001	0.00	0.995	5.19	< 0.001
Diabetes.combined	0.34	0.153	1.12	0.009	0.72	0.037	0.35	0.150	3.08	< 0.001
NumChild	1.36	0.017	7.83	< 0.001	4.06	< 0.001	0.00	0.372	4.92	< 0.001
Age	13.28	< 0.001	24.87	< 0.001	17.66	< 0.001	0.07	0.937	20.25	< 0.001
Quarter	0.42	0.470	1.33	0.046	0.64	0.283	0.48	0.414	0.62	0.213
WrkStat	0.44	0.106	3.39	< 0.001	0.21	0.262	0.44	0.103	2.65	< 0.001
ethgrp5	0.44	0.620	1.00	0.200	0.58	0.482	1.13	0.147	0.46	0.506
eqv3	0.08	0.789	0.21	0.528	0.01	0.972	0.42	0.285	0.50	0.162
cigsta3	1.88	0.003	2.42	0.001	2.40	0.002	1.00	0.050	2.33	< 0.001
dnoft	2.13	0.045	3.37	0.002	2.92	0.007	1.63	0.135	1.39	0.121
bmival	10.32	< 0.001	9.19	< 0.001	11.32	< 0.001	2.22	0.004	5.85	< 0.001
whgval	12.02	< 0.001	12.11	< 0.001	13.90	< 0.001	2.80	< 0.001	10.26	< 0.001
MN	1.13	0.147	1.98	0.018	1.54	0.055	1.55	0.053	0.27	0.738
EnergyDkJ	0.17	0.796	0.04	0.974	0.08	0.927	0.61	0.301	0.21	0.679
ProteingD	0.29	0.628	0.07	0.931	0.13	0.851	1.17	0.072	0.46	0.341
FatgD	0.36	0.543	0.03	0.979	0.20	0.754	1.45	0.033	0.35	0.471
GlucosegD	0.11	0.887	0.13	0.861	0.09	0.910	1.74	0.015	0.30	0.542
SodiummgD	0.06	0.954	0.34	0.565	0.15	0.822	0.36	0.544	0.24	0.623
FolateugplussuppsD	0.66	0.266	1.42	0.036	1.09	0.088	2.02	0.007	0.26	0.597
qual7	0.86	0.161	0.56	0.340	0.13	0.849	0.85	0.165	0.92	0.083
Region	1.41	0.075	1.27	0.107	1.26	0.108	1.02	0.189	0.56	0.400
SalHowC	0.15	0.646	0.03	0.920	0.03	0.921	0.10	0.732	0.04	0.873

The individual contributions of E-iAs_ing,rice_ and all the potential confounders to the variability of hypertension risks were quantified through a generalized linear model (GLM) (contributions (%) = 100*(null deviance-residual deviance)/null deviance) (Bjorndal et al. 2013)

DBP add 10: Omron valid mean diastolic blood pressure (DBP) incremented by 10 mmHg is added if anti-hypertension medication is taken (mmHg); SBP add 10: Omron valid mean systolic blood pressure (SBP) incremented by 10 mmHg is added if anti-hypertension medication is taken (mmHg); AP: mean arterial pressure (mmHg); meanPulse: mean pulse pressure (mmHg); general hypertension: whether participants were diagnosed as general hypertension; E-iAs_ing,rice_: daily inorganic arsenic (iAs) intake from rice and rice products; E-iAs_ing,grain_: daily iAs intake from grain and grain-based products; E-iAs_ing,water_: daily iAs intake from drinking water; surveyyr: NDNS RP 7–8 Survey year; Sex: gender; EnergyDkJ: intake of total energy per day (KJ) for diet only; ProteingD: intake of protein per day (g) for diet only; FatgD: intake of fat per day (g) for diet only; GlucosegD: intake of glucose per day (g) for diet only; SodiummgD: intake of sodium per day (mg) for diet only; FolateugplussuppsD: intake of folate (µg) per day for both diets and supplements; MN: daily intake of several micro-nutrients (potassium (mg) including supplements, calcium (mg) including supplements, magnesium (mg) including supplements, iron (mg) including supplements, copper (mg) including supplements, zinc (mg) including supplements, retinol (mg) including supplements, vitamin A (retinol equivalents) (µg) including supplements, vitamin D (µg) including supplements, vitamin E (mg) including supplements, thiamin (mg) including supplements, riboflavin (mg) including supplements, niacin equivalent (mg) including supplements, vitamin B6 (mg) including supplements, vitamin B12 (µg) including supplements, vitamin C (mg) including supplements, iodine (µg) including supplements, selenium (µg) including supplements); region: country people live; NumChild: number of children aged between 0 and 15; age: age of respondent 16 + ; SalHowC: how often salt added during cooking; Quarter: fieldwork quarter; qual7: qualifications gained; WrkStat: economic status (working condition); ethgrp5: ethnic group; eqv3: equivalized household income; HessCon: whether have any physical/mental health condition/illnesses for 12 months or more; Diabetes.combined: whether respondent is diabetic; cigsta3: cigarette smoking status; dnoft: frequency of alcohol consumption in past 12 months (including non-drinkers); bmival: BMI (kg/m^2^); whgval: waist-hip ratio groups

*The *p* value for the contribution of each single factor was obtained by analysis of variance (ANOVA) with type II error

Aiming at quantifying the importance of the interactive effects between E-iAs_ing,rice_ and all the potential confounders, we also calculated the relative excess risks and their contributions. Based on the results, there were significantly interactive effects only between SodiummgD and E-iAs_ing,rice_ on the variability of meanPulse (Table 5).

**Table 5:** Importance of the interactive effects between E-iAs_ing,rice_ and all the potential confounders on the variability of hypertension risks. Data from NDNS RP 7–8 (MRC Elsie Widdowson Laboratory and NatCen Social Research 2019) with population satisfying inclusion criteria as detailed in the text (*N* = 598)

	DBP add 10			SBP add 10			AP			meanPulse			general hypertension		
	RERI^a^	Contribution (%)	*p* value*	RERI^a^	Contribution (%)	*p* value*	RERI^a^	Contribution (%)	*p* value*	RERI^a^	Contribution (%)	*p* value*	RERI^a^	Contribution (%)	*p* value*
E-iAs_ing,water_	0.00	0.00	0.864	0.00	0.29	0.191	0.00	0.04	0.614	0.00	0.17	0.318	− 0.05	0.44	0.075
E-iAs_ing,grain_	0.00	0.41	0.118	0.00	0.27	0.202	0.00	0.40	0.123	0.00	0.09	0.458	0.03	0.30	0.141
Sex	0.00	0.07	0.513	0.00	3.45	0.530	0.00	0.08	0.490	0.00	2.30	0.346	0.00	0.01	0.833
surveyyr	0.00	0.26	0.210	0.00	0.04	0.611	0.00	0.17	0.318	0.00	0.25	0.221	0.06	0.22	0.208
HessCon	0.00	0.04	0.631	0.00	0.05	0.581	0.00	0.00	0.969	0.00	0.05	0.597	− 1.20	0.04	0.614
Diabetes.combined	0.01	0.10	0.439	0.00	0.08	0.502	0.01	0.10	0.433	0.00	0.05	0.576	0.02	0.03	0.671
NumChild	0.02	0.46	0.257	0.01	0.25	0.478	0.01	0.41	0.299	0.01	0.06	0.836	0.45	0.17	0.566
Age	− 0.08	1.04	0.102	0.01	0.46	0.438	− 0.04	0.87	0.161	0.01	0.62	0.300	− 5.87	0.88	0.166
Quarter	0.01	0.17	0.800	0.06	0.19	0.778	0.03	0.08	0.926	− 0.02	0.65	0.274	1.34	0.07	0.921
WrkStat	0.00	0.07	0.533	0.00	0.05	0.572	0.00	0.07	0.526	0.00	0.37	0.138	0.01	0.06	0.528
ethgrp5	− 0.08	0.24	0.843	− 0.07	0.55	0.519	− 0.08	0.33	0.744	0.24	0.15	0.924	− 1.20	1.32	0.049
eqv3	0.01	0.29	0.418	0.01	0.25	0.483	0.01	0.31	0.397	0.01	0.94	0.061	0.19	0.15	0.594
cigsta3	− 0.05	0.50	0.228	− 0.06	1.14	0.033	− 0.06	0.86	0.077	0.00	0.22	0.518	− 0.77	0.46	0.194
dnoft	0.05	1.07	0.390	0.08	0.94	0.476	0.06	1.05	0.404	0.05	1.38	0.226	1.41	1.17	0.213
bmival	− 0.03	0.51	0.388	− 0.05	0.46	0.436	− 0.04	0.54	0.358	0.03	0.64	0.286	− 0.83	0.23	0.675
whgval	− 0.04	0.10	0.738	− 0.03	0.61	0.163	− 0.04	14.72	0.431	− 0.01	0.01	0.985	− 0.99	0.02	0.950
MN	− 0.02	0.48	0.586	− 0.02	0.83	0.294	− 0.02	0.71	0.381	− 0.02	1.86	0.025	− 0.09	1.36	0.044
EnergyDkJ	0.00	0.81	0.185	0.00	0.62	0.301	0.00	0.79	0.195	− 0.01	1.57	0.025	0.14	0.51	0.298
ProteingD	0.00	0.75	0.218	− 0.01	0.70	0.247	0.00	0.72	0.234	− 0.03	0.74	0.219	0.01	0.63	0.210
FatgD	0.02	0.28	0.651	0.01	0.35	0.557	0.02	0.33	0.581	0.01	0.35	0.557	0.37	0.44	0.370
GlucosegD	0.00	0.20	0.758	0.01	0.35	0.561	0.01	0.27	0.659	0.02	0.26	0.667	0.12	0.10	0.873
SodiummgD	− 0.01	0.25	0.682	− 0.01	0.15	0.826	− 0.01	0.20	0.760	0.01	1.59	0.023	0.00	0.21	0.684
FolateugplussuppsD	− 0.01	0.05	0.958	− 0.02	0.05	0.959	− 0.02	0.04	0.969	0.00	0.65	0.279	− 0.07	0.57	0.248
qual7	0.00	0.22	0.732	0.01	0.51	0.387	0.00	0.30	0.625	0.00	0.20	0.762	0.18	0.45	0.358
Region	− 0.05	0.27	0.810	− 0.02	0.41	0.663	− 0.04	0.24	0.837	− 0.01	6.81	0.874	0.14	0.97	0.138
SalHowC	− 0.02	0.08	0.790	0.00	0.21	0.531	− 0.01	0.12	0.708	0.00	0.33	0.379	− 0.07	0.12	0.643

The interactive contributions between E-iAs_ing,rice_ and all the potential confounders to the variability of hypertension risks were quantified through a generalized linear model (GLM) (contributions (%) = 100*(null deviance-residual deviance)/null deviance) (Bjorndal et al. 2013)

DBP add 10: Omron valid mean diastolic blood pressure (DBP) incremented by 10 mmHg is added if anti-hypertension medication is taken (mmHg); SBP add 10: Omron valid mean systolic blood pressure (SBP) incremented by 10 mmHg is added if anti-hypertension medication is taken (mmHg); AP: mean arterial pressure (mmHg); meanPulse: mean pulse pressure (mmHg); general hypertension: whether participants were diagnosed as general hypertension; E-iAs_ing,rice_: daily inorganic arsenic (iAs) intake from rice and rice products; E-iAs_ing,water_: daily iAs intake from drinking water; E-iAs_ing,grain_: daily iAs intake from grain and grain-based products; surveyyr: NDNS RP 7–8 Survey year; Sex: gender; EnergyDkJ: intake of total energy per day (KJ) for diet only; ProteingD: intake of protein per day (g) for diet only; FatgD: intake of fat per day (g) for diet only; GlucosegD: intake of glucose per day (g) for diet only; SodiummgD: intake of sodium per day (mg) for diet only; FolateugplussuppsD: intake of folate (µg) per day for both diets and supplements; MN: daily intake of several micro-nutrients (potassium (mg) including supplements, calcium (mg) including supplements, magnesium (mg) including supplements, iron (mg) including supplements, copper (mg) including supplements, zinc (mg) including supplements, retinol (mg) including supplements, vitamin A (retinol equivalents) (µg) including supplements, vitamin D (µg) including supplements, vitamin E (mg) including supplements, thiamin (mg) including supplements, riboflavin (mg) including supplements, niacin equivalent (mg) including supplements, vitamin B6 (mg) including supplements, vitamin B12 (µg) including supplements, vitamin C (mg) including supplements, iodine (µg) including supplements, selenium (µg) including supplements); region: country people live; NumChild: number of children aged between 0 and 15; age: age of respondent 16 + ; SalHowC: how often salt added during cooking; quarter: fieldwork quarter; qual7: qualifications gained; WrkStat: economic status (working condition); ethgrp5: ethnic group; eqv3: equivalized household income; HessCon: whether have any physical/mental health condition/illnesses for 12 months or more; Diabetes.combined: whether respondent is diabetic; cigsta3: cigarette smoking status; dnoft: frequency of alcohol consumption in past 12 months (including non-drinkers); bmival: BMI (kg/m^2^); whgval: waist-hip ratio groups

*The *p* values for interaction were obtained by adding a cross-product term between continuous E-iAs_ing,rice_ and different confounders to the linear model via an analysis of variance (ANOVA) with type II error

^a^The importance of two-way interactive effects was calculated based on the relative excess risk for interaction (RERI) according to Chen et al. (2011b): ${\text{RERI}} = {\text{e}}^{{\left( {\beta 1 + \beta 2 + \beta 3} \right)}} - {\text{e}}^{\beta 1} - {\text{e}}^{\beta 2} + 1,$ where: *β*1 is the continuous coefficient of E-iAs_ing,rice_; *β*2 is the coefficient of the potential confounder; and *β*3 is the interactive term coefficient, with an estimation over zero indicating the presence of synergetic effects

### The relationships between E-iAs_ing,rice_ and hypertension risks

After comparing models with and without considering the effects of E-iAs_ing,rice_ (Table 6), we found that adding E-iAs_ing,rice_ did not contribute to significantly better models for all the blood pressure endpoints, indicating that the association between E-iAs_ing,rice_ and hypertension risks is weak.

**Table 6:** Comparison of models including some important risks factors with and without E-iAs_ing,rice_ as a confounder for all the blood pressure endpoints. Data from NDNS RP 7–8 (MRC Elsie Widdowson Laboratory and NatCen Social Research 2019) with population satisfying inclusion criteria as detailed in the text (*N* = 598)

Models	DBP add 10		SBP add 10		AP		meanPulse		General hypertension	
	AIC	Contribution (%)	AIC	Contribution (%)	AIC	Contribution (%)	AIC	Contribution (%)	AIC	Contribution (%)
Model with E-iAs_ing,rice_	4570.8	23.8	4999.0	34.2	4648.7	26.3	4493.6	10.8	565.5	25.1
Model without E-iAs_ing,rice_	4571.1	23.5	5000.0	33.9	4649.0	26.0	4491.6	10.8	564.1	25.0

DBP add 10: Omron valid mean diastolic blood pressure (DBP) incremented by 10 mmHg is added if anti-hypertension medication is taken (mmHg); SBP add 10: Omron valid mean systolic blood pressure (SBP) incremented by 10 mmHg is added if anti-hypertension medication is taken (mmHg); AP: mean arterial pressure (mmHg); meanPulse: mean pulse pressure (mmHg); general hypertension: whether participants were diagnosed as general hypertension; E-iAs_ing,rice_: daily inorganic arsenic (iAs) intake from rice and rice products; E-iAs_ing,grain_: daily iAs intake from grain and grain-based products; Sex: gender; FatgD: intake of fat per day (g) for diet only; FolateugplussuppsD: intake of folate (µg) per day for both diets and supplements; MN: daily intake of several micro-nutrients (Potassium (mg) including supplements, calcium (mg) including supplements, magnesium (mg) including supplements, iron (mg) including supplements, copper (mg) including supplements, zinc (mg) including supplements, retinol (mg) including supplements, vitamin A (retinol equivalents) (µg) including supplements, vitamin D (µg) including supplements, vitamin E (mg) including supplements, thiamin (mg) including supplements, riboflavin (mg) including supplements, niacin equivalent (mg) including supplements, vitamin B6 (mg) including supplements, vitamin B12 (µg) including supplements, vitamin C (mg) including supplements, iodine (µg) including supplements, selenium (µg) including supplements); region: country people live; NumChild: number of children aged between 0 and 15; age: age of respondent 16 + ; Quarter: fieldwork quarter; qual7: qualifications gained; HessCon: whether have any physical/mental health condition/illnesses for 12 months or more; Diabetes.combined: whether respondent is diabetic; cigsta3: cigarette smoking status; bmival: BMI (kg/m^2^); whgval: waist-hip ratio groups

For DBP add 10: model with DBP add 10 as dependent variable with E-iAs_ing,rice_ was constructed by ‘stepwise’ function in R language based on AIC values which was adjusted by E-iAs_ing,rice_, age, bmival, whgval, qual7, E-iAs_ing,grain_, HessCon and region; Model with DBP add 10 as dependent variable without E-iAs_ing,rice_ was constructed by ‘stepwise’ function in R language based on AIC values which was adjusted by age, bmival, whgval, qual7, E-iAs_ing,grain_, HessCon and region

For SBP add 10: model with SBP add 10 as dependent variable with E-iAs_ing,rice_ was constructed by ‘stepwise’ function in R language based on AIC values which was adjusted by E-iAs_ing,rice_, age, bmival, Sex, Quarter, HessCon, NumChild, MN, whgval; Model with SBP add 10 as dependent variable without E-iAs_ing,rice_ was constructed by ‘stepwise’ function in R language based on AIC values which was adjusted by age, bmival, Sex, Quarter, HessCon, NumChild, MN, whgval

For AP: model with AP as dependent variable with E-iAs_ing,rice_ was constructed by ‘stepwise’ function in R language based on AIC values which was adjusted by E-iAs_ing,rice_, age, bmival, whgval, Sex, HessCon, E-iAs_ing,grain_, MN; Model with AP as dependent variable without E-iAs_ing,rice_ was constructed by ‘stepwise’ function in R language based on AIC values which was adjusted by age, bmival, whgval, Sex, HessCon, E-iAs_ing,grain_, MN

For meanPulse: model with meanPulse as dependent variable with E-iAs_ing,rice_ was constructed by ‘stepwise’ function in R language based on AIC values which was adjusted by E-iAs_ing,rice_, whgval, Sex, FolateugplussuppsD, bmival, MN, cigsta3, FatgD; Model with meanPulse as dependent variable without E-iAs_ing,rice_ was constructed by ‘stepwise’ function in R language based on AIC values which was adjusted by whgval, Sex, FolateugplussuppsD, bmival, MN, cigsta3, FatgD

For the odds ratio of general hypertension: model with the odds ratio of general hypertension as dependent variable with E-iAs_ing,rice_ was constructed by ‘stepwise’ function in R language based on AIC values which was adjusted by E-iAs_ing,rice_, age, bmival, Diabetes.combined, HessCon; Model with the odds ratio of general hypertension as dependent variable without E-iAs_ing,rice_ was constructed by ‘stepwise’ function in R language based on AIC values which was adjusted by age, bmival, Diabetes.combined, HessCon

To further analyse the dose–response associations between E-iAs_ing,rice_ and hypertension risks, univariate and multivariate GLMs were used in the linear regression and logistic regression models. In general, E-iAs_ing,rice_ was negatively but not significantly related to all the blood pressure endpoints (DBP add 10, SBP add 10, AP, meanPulse and general hypertension) and the associations were stronger in the population with the highest intake level (Tables 7, 8, 9, 10, 11). To be specific, for the continuous analysis of the best-fitted linear models using AIC as the primary selection criterion (Model 4 in Tables 7, 8, 9, 10, 11), every increase of 1 µg/person/day E-iAs_ing,rice_ was associated with lower hypertension risks, ranging from a decrease of 0.2% DBP add 10 to 2% odds ratio of general hypertension. For the categorical results of the best-fitted linear model, the higher three quartiles of iAs intake were associated with decreased odds ratios of general hypertension [1.00 (referent), 0.76 (95% CI 0.41, 1.28), 0.75 (95% CI 0.42, 1.34), and 0.54 (95% CI 0.30, 0.98)] in the overall population with similar pattern being found for other blood pressure endpoints as well (Model 4 in Tables 7, 8, 9, 10, 11). Moreover, taking anti-hypertension medications which likely induce artificially lower blood pressure cannot confound our results as excluding participants with such medications yielded results similar to those described above (Table S3). In addition, we examined the assumption of the nonlinear relationships by including higher order polynomial terms of iAs intake variable in the models, but found no significant departure from linearity based on the significance for the higher order terms (nonlinear models in Tables 7, 8, 9, 10, 11).

**Table 7:** Modelling analysis of the categorical and continuous association of DBP add 10 with E-iAs_ing,rice_ (µg/person/day). Data from NDNS RP 7–8 (MRC Elsie Widdowson Laboratory and NatCen Social Research 2019) with population satisfying inclusion criteria as detailed in the text (*N* = 598)

Model	Quartile of E-iAs_ing,rice_ (µg/person/day)							DBP add 10 per 1 µg/person/day increase of E-iAs_ing,rice_	*p* value for trend	AIC	Contributions (%)
	Quartile 1 (0.00–0.00)	Quartile 2 (0.00–0.565)	*p* value*	Quartile 3 (0.638–3.79)	*p* value*	Quartile 4 (3.79–41.8)	*p* value*				
Model 1	1 (Referent)	0.97 (0.93, 1.00)	0.053	0.96 (0.93, 1.00)	0.032	0.95 (0.92, 0.99)	0.012	0.99_7_ (0.99_4_, 1.00)	0.041	4694.9	0.7
Model 2	1 (Referent)	0.96 (0.92, 1.00)	0.046	0.97 (0.93, 1.00)	0.054	0.95 (0.91, 0.99)	0.006	0.99_7_ (0.99_4_, 1.00)	0.081	4620.3	29.5
Model 3	1 (Referent)	0.98 (0.95, 1.01)	0.252	0.97 (0.94, 1.01)	0.123	0.96 (0.93, 1.00)	0.031	0.99_8_ (0.99_5_, 1.00)	0.112	4582.3	26.8
Model 4	1 (Referent)	0.98 (0.95, 1.02)	0.338	0.97 (0.94, 1.01)	0.100	0.97 (0.93, 1.00)	0.035	0.99_8_ (0.99_5_, 1.00)	0.132	4570.8	23.8
Nonlinear model	DBP add10 ~ E-iAs_ing,rice_ + E-iAs_ing,rice_^2^ + age + bmival + whgval + qual1 + E-iAs_ing,grain_ + HessCon + region								0.218	4571.2	24.0

The differences of DBP add 10 across four quartiles were obtained from Wald tests for E-iAs_ing,rice_ coefficients, and the *p* value for linear and nonlinear trends was obtained from an analysis of variance (ANOVA) analysis with type II error where E-iAs_ing,rice_ is a continuous measure of intake

DBP add 10: Omron valid mean diastolic blood pressure (DBP) incremented by 10 mmHg is added if anti-hypertension medication is taken (mmHg); E-iAs_ing,rice_: daily inorganic arsenic (iAs) intake from rice and rice products; E-iAs_ing,water_: daily iAs intake from drinking water; E-iAs_ing,grain_: daily iAs intake from grain and grain-based products; surveyyr: NDNS RP 7–8 Survey year; Sex: gender; EnergyDkJ: intake of total energy per day (KJ) for diet only; ProteingD: intake of protein per day (g) for diet only; FatgD: intake of fat per day (g) for diet only; GlucosegD: intake of glucose per day (g) for diet only; SodiummgD: intake of sodium per day (mg) for diet only; FolateugplussuppsD: intake of folate (µg) per day for both diets and supplements; MN: daily intake of several micro-nutrients (potassium (mg) including supplements, calcium (mg) including supplements, magnesium (mg) including supplements, iron (mg) including supplements, copper (mg) including supplements, zinc (mg) including supplements, retinol (mg) including supplements, vitamin A (retinol equivalents) (µg) including supplements, vitamin D (µg) including supplements, vitamin E (mg) including supplements, thiamin (mg) including supplements, riboflavin (mg) including supplements, niacin equivalent (mg) including supplements, vitamin B6 (mg) including supplements, vitamin B12 (µg) including supplements, vitamin C (mg) including supplements, iodine (µg) including supplements, selenium (µg) including supplements); region: country people live; NumChild: number of children aged between 0 and 15; age: age of respondent 16 + ; SalHowC: how often salt added during cooking; Quarter: fieldwork quarter; qual7: qualifications gained; WrkStat: economic status (working condition); ethgrp5: ethnic group; eqv3: Equivalized household income; HessCon: whether have any physical/mental health condition/illnesses for 12 months or more; Diabetes.combined: whether respondent is diabetic; cigsta3: cigarette smoking status; dnoft: frequency of alcohol consumption in past 12 months (including non-drinkers); bmival: BMI (kg/m^2^); whgval: waist-hip ratio groups

Model 1: crude with DBP add 10 only (univariate model)

Model 2: full model, adjusted by E-iAs_ing,water_, E-iAs_ing,grain_, age, bmival, cigsta3, region, Diabetes.combined, dnoft, eqv3, ethgrp5, SalHowC, HessCon, MN, NumChild, qual7, Quarter, Sex, surveyyr, whgval, WrkStat, EnergyDkJ, ProteingD, FatgD, GlucosegD, SodiummgD, FolateugplussuppsD

Model 3: adjusted by variables with *p* value lower than 0.2 in the univariate analysis: E-iAs_ing,water_, E-iAs_ing,grain_, age, bmival, cigsta3, region, Diabetes.combined, dnoft, HessCon, MN, NumChild, qual7, Sex, whgval, WrkStat

Model 4: constructed by ‘stepwise’ function in R language based on AIC values which were adjusted by age, bmival, whgval, qual7, E-iAs_ing,grain_, HessCon and region

*Compared with Quartile 1 (referent group) (0.00–0.00 µg/person/day)

**Table 8:** Modelling analysis of the categorical and continuous association of SBP add 10 with E-iAs_ing,rice_ (µg/person/day). Data from NDNS RP 7–8 (MRC Elsie Widdowson Laboratory and NatCen Social Research 2019) with population satisfying inclusion criteria as detailed in the text (*N* = 598)

Model	Quartile of E-iAs_ing,rice_ (µg/person/day)							SBP add 10 per 1 µg/person/day increase of E-iAs_ing,rice_	*p* value for trend	AIC	Contributions (%)
	Quartile 1 (0.00–0.00)	Quartile 2 (0.00–0.565)	*p* value*	Quartile 3 (0.638–3.79)	*p* value*	Quartile 4 (3.79–41.8)	*p* value*				
Model 1	1 (Referent)	0.95 (0.92, 0.98)	0.004	0.96 (0.93, 1.00)	0.008	0.94 (0.91, 0.97)	< 0.001	0.99_7_ (0.99_4_, 0.99_9_)	0.019	5209.6	0.9
Model 2	1 (Referent)	0.96 (0.93, 1.00)	0.032	0.98 (0.95, 1.01)	0.125	0.95 (0.92, 0.98)	0.003	0.99_8_ (0.99_5_, 1.00)	0.095	5055.3	38.4
Model 3	1 (Referent)	0.96 (0.92, 0.99)	0.013	0.98 (0.95, 1.01)	0.115	0.95 (0.92, 0.98)	0.002	0.99_8_ (0.99_5_, 1.00)	0.101	5025.3	36.8
Model 4	1 (Referent)	0.97 (0.94, 1.00)	0.029	0.98 (0.95, 1.01)	0.171	0.96 (0.93, 1.00)	0.006	0.99_8_ (0.99_6_, 1.00)	0.080	4999.2	34.6
Nonlinear model	SBP add10 ~ E-iAs_ing,rice_ + E-iAs_ing,rice_^2^ + age + bmival + Sex + Quarter + NumChild + HessCon + MN + whgval								0.426	5000.5	34.7

The differences of SBP add 10 across four quartiles were obtained from Wald tests for E-iAs_ing,rice_ coefficients, and the *p* value for linear and nonlinear trends was obtained from an analysis of variance (ANOVA) with type II error where E-iAs_ing,rice_ is a continuous measure of intake

SBP add 10: Omron valid mean systolic blood pressure (SBP) incremented by 10 mmHg is added if anti-hypertension medication is taken (mmHg); E-iAs_ing,rice_: daily inorganic arsenic (iAs) intake from rice and rice products; E-iAs_ing,water_: daily iAs intake from drinking water; E-iAs_ing,grain_: daily iAs intake from grain and grain-based products; surveyyr: NDNS RP 7–8 Survey year; Sex: gender; EnergyDkJ: intake of total energy per day (KJ) for diet only; ProteingD: intake of protein per day (g) for diet only; FatgD: intake of fat per day (g) for diet only; GlucosegD: intake of glucose per day (g) for diet only; SodiummgD: intake of sodium per day (mg) for diet only; FolateugplussuppsD: intake of folate (µg) per day for both diets and supplements; MN: daily intake of several micro-nutrients (Potassium (mg) including supplements, calcium (mg) including supplements, magnesium (mg) including supplements, iron (mg) including supplements, copper (mg) including supplements, zinc (mg) including supplements, retinol (mg) including supplements, vitamin A (retinol equivalents) (µg) including supplements, vitamin D (µg) including supplements, vitamin E (mg) including supplements, thiamin (mg) including supplements, riboflavin (mg) including supplements, niacin equivalent (mg) including supplements, vitamin B6 (mg) including supplements, vitamin B12 (µg) including supplements, vitamin C (mg) including supplements, iodine (µg) including supplements, selenium (µg) including supplements); region: country people live; NumChild: number of children aged between 0 and 15; age: age of respondent 16 + ; SalHowC: how often salt added during cooking; Quarter: fieldwork quarter; qual7: qualifications gained; WrkStat: economic status (working condition); ethgrp5: ethnic group; eqv3: Equivalized household income; HessCon: whether have any physical/mental health condition/illnesses for 12 months or more; Diabetes.combined: whether respondent is diabetic; cigsta3: cigarette smoking status; dnoft: frequency of alcohol consumption in past 12 months (including non-drinkers); bmival: BMI (kg/m^2^); whgval: waist-hip ratio groups

Model 1: crude with SBP add 10 only (univariate model)

Model 2: full model, adjusted by E-iAs_ing,water_, E-iAs_ing,grain_, age, bmival, cigsta3, region, Diabetes.combined, dnoft, eqv3, ethgrp5, SalHowC, HessCon, MN, NumChild, qual7, Quarter, Sex, surveyyr, whgval, WrkStat, EnergyDkJ, ProteingD, FatgD, GlucosegD, SodiummgD, FolateugplussuppsD

Model 3: adjusted by variables with *p* value lower than 0.2 in the univariate analysis: E-iAs_ing,water_, age, bmival, cigsta3, region, Diabetes.combined, dnoft, ethgrp5, HessCon, MN, NumChild, Quarter, Sex, surveyyr, whgval, WrkStat, FolateugplussuppsD

Model 4: constructed by ‘stepwise’ function in R language based on AIC values which were adjusted by age, bmival, Sex, Quarter, HessCon, NumChild, MN, whgval

*Compared with Quartile 1 (referent group) (0.00–0.00 µg/person/day)

**Table 9:** Modelling analysis of the categorical and continuous association of AP with E-iAs_ing,rice_ (µg/person/day). Data from NDNS RP 7–8 (MRC Elsie Widdowson Laboratory and NatCen Social Research 2019) with population satisfying inclusion criteria as detailed in the text (*N* = 598)

Model	Quartile of E-iAs_ing,rice_ (µg/person/day)							AP per 1 µg/person/day increase of E-iAs_ing,rice_	*p* value for trend	AIC	Contributions (%)
	Quartile 1 (0.00–0.00)	Quartile 2 (0.00–0.565)	*p* value*	Quartile 3 (0.638–3.79)	*p* value*	Quartile 4 (3.79–41.8)	*p* value*				
Model 1	1 (Referent)	0.96 (0.93, 0.99)	0.012	0.96 (0.93, 0.99)	0.011	0.95 (0.92, 0.98)	0.001	0.99_7_ (0.99_4_, 1.00)	0.019	4805.5	0.9
Model 2	1 (Referent)	0.96 (0.96, 1.00)	0.028	0.97 (0.94, 1.00)	0.059	0.95 (0.92, 0.98)	0.003	0.99_8_ (0.99_5_, 1.00)	0.067	4704.2	32.7
Model 3	1 (Referent)	0.98 (0.95, 1.00)	0.087	0.98 (0.95, 1.01)	0.114	0.96 (0.93, 0.99)	0.005	0.99_8_ (0.99_5_, 1.00)	0.069	4665.0	30.8
Model 4	1 (Referent)	0.97 (0.95, 1.00)	0.075	0.98 (0.95, 1.00)	0.081	0.96 (0.93, 0.99)	0.006	0.99_8_ (0.99_5_, 1.00)	0.060	4648.4	27.5
Nonlinear model	AP ~ E-iAs_ing,rice_ + E-iAs_ing,rice_^2^ + age + bmival + whgval + Sex + HessCon + E-iAs_ing,grain_ + MN								0.248	4649.1	27.7

The differences of AP across four quartiles were obtained from Wald tests for E-iAs_ing,rice_ coefficients, and the *p* value for linear and nonlinear trends was obtained from an analysis of variance (ANOVA) with type II error where E-iAs_ing,rice_ is a continuous measure of intake

AP: mean arterial pressure (mmHg); E-iAs_ing,rice_: daily inorganic arsenic (iAs) intake from rice and rice products; E-iAs_ing,water_: daily iAs intake from drinking water; E-iAs_ing,grain_: daily iAs intake from grain and grain-based products; surveyyr: NDNS RP 7–8 Survey year; Sex: gender; EnergyDkJ: intake of total energy per day (KJ) for diet only; ProteingD: intake of protein per day (g) for diet only; FatgD: intake of fat per day (g) for diet only; GlucosegD: intake of glucose per day (g) for diet only; SodiummgD: intake of sodium per day (mg) for diet only; FolateugplussuppsD: intake of folate (µg) per day for both diets and supplements; MN: daily intake of several micro-nutrients (Potassium (mg) including supplements, calcium (mg) including supplements, magnesium (mg) including supplements, iron (mg) including supplements, copper (mg) including supplements, zinc (mg) including supplements, retinol (mg) including supplements, vitamin A (retinol equivalents) (µg) including supplements, vitamin D (µg) including supplements, vitamin E (mg) including supplements, thiamin (mg) including supplements, riboflavin (mg) including supplements, niacin equivalent (mg) including supplements, vitamin B6 (mg) including supplements, vitamin B12 (µg) including supplements, vitamin C (mg) including supplements, iodine (µg) including supplements, selenium (µg) including supplements); region: country people live; NumChild: number of children aged between 0 and 15; age: age of respondent 16 + ; SalHowC: how often salt added during cooking; Quarter: fieldwork quarter; qual7: qualifications gained; WrkStat: economic status (working condition); ethgrp5: ethnic group; eqv3: equivalized household income; HessCon: whether have any physical/mental health condition/illnesses for 12 months or more; Diabetes.combined: whether respondent is diabetic; cigsta3: cigarette smoking status; dnoft: frequency of alcohol consumption in past 12 months (including non-drinkers); bmival: BMI (kg/m^2^); whgval: waist-hip ratio groups

Model 1: crude with AP only (univariate model)

Model 2: full model, adjusted by E-iAs_ing,water_, E-iAs_ing,grain_, age, bmival, cigsta3, region, Diabetes.combined, dnoft, eqv3, ethgrp5, SalHowC, HessCon, MN, NumChild, qual7, Quarter, Sex, surveyyr, whgval, WrkStat, EnergyDkJ, ProteingD, FatgD, GlucosegD, SodiummgD, FolateugplussuppsD

Model 3: adjusted by variables with *p* value lower than 0.2 in the univariate analysis: E-iAs_ing,water_, E-iAs_ing,grain_, age, bmival, cigsta3, region, Diabetes.combined, dnoft, HessCon, MN, NumChild, qual7, Sex, whgval, FolateugplussuppsD

Model 4: constructed by ‘stepwise’ function in R language based on AIC values which were adjusted by age, bmival, whgval, Sex, HessCon, E-iAs_ing,grain_, MN

*Compared with Quartile 1 (referent group) (0.00–0.00 µg/person/day)

**Table 10:** Modelling analysis of the categorical and continuous association of meanPulse with E-iAs_ing,rice_ (µg/person/day). Data from NDNS RP 7–8 (MRC Elsie Widdowson Laboratory and NatCen Social Research 2019) with population satisfying inclusion criteria as detailed in the text (*N* = 598)

Model	Quartile of E-iAs_ing,rice_ (µg/person/day)							meanPulse per 1 µg/person/day increase of E-iAs_ing,rice_	*p* value for trend	AIC	Contributions (%)
	Quartile 1 (0.00–0.00)	Quartile 2 (0.00–0.565)	*p* value*	Quartile 3 (0.638–3.79)	*p* value*	Quartile 4 (3.79–41.8)	*p* value*				
Model 1	1 (Referent)	1.00 (0.96, 1.03)	0.816	1.00 (0.96, 1.03)	0.837	0.98 (0.94, 1.01)	0.187	0.99_9_ (0.99_7_, 1.00)	0.661	4525.9	0.0_3_
Model 2	1 (Referent)	0.98 (0.94, 1.02)	0.382	0.98 (0.95, 1.02)	0.401	0.97 (0.93, 1.01)	0.128	1.00 (0.99_7_, 1.00)	0.970	4533.8	18.5
Model 3	1 (Referent)	1.00 (0.97, 1.03)	0.927	1.00 (0.97, 1.03)	0.995	0.99 (0.95, 1.02)	0.416	1.00 (0.99_7_, 1.00)	0.773	4517.1	15.3
Model 4	1 (Referent)	0.99 (0.96, 1.03)	0.677	1.00 (0.96, 1.03)	0.764	0.98 (0.94, 1.01)	0.174	1.00 (0.99_7_, 1.00)	0.913	4493.6	10.8
Nonlinear model	meanPulse ~ E-iAs_ing,rice_ + E-iAs_ing,rice_^2^ + whgval + Sex + FolateugplussuppsD + bmival + MN + cigsta3 + FatgD								0.207	4494.0	11.1

The differences of meanPulse across four quartiles were obtained from Wald tests for E-iAs_ing,rice_ coefficients, and the *p* value for linear and nonlinear trends was obtained from an analysis of variance (ANOVA) with type II error where E-iAs_ing,rice_ is a continuous measure of intake

meanPulse: mean pulse pressure (mmHg); E-iAs_ing,rice_: daily inorganic arsenic (iAs) intake from rice and rice products; E-iAs_ing,water_: daily iAs intake from drinking water; E-iAs_ing,grain_: daily iAs intake from grain and grain-based products; surveyyr: NDNS RP 7–8 Survey year; Sex: gender; EnergyDkJ: intake of total energy per day (KJ) for diet only; ProteingD: intake of protein per day (g) for diet only; FatgD: intake of fat per day (g) for diet only; GlucosegD: intake of glucose per day (g) for diet only; SodiummgD: intake of sodium per day (mg) for diet only; FolateugplussuppsD: intake of folate (µg) per day for both diets and supplements; MN: daily intake of several micro-nutrients (Potassium (mg) including supplements, calcium (mg) including supplements, magnesium (mg) including supplements, iron (mg) including supplements, copper (mg) including supplements, zinc (mg) including supplements, retinol (mg) including supplements, vitamin A (retinol equivalents) (µg) including supplements, vitamin D (µg) including supplements, vitamin E (mg) including supplements, thiamin (mg) including supplements, riboflavin (mg) including supplements, niacin equivalent (mg) including supplements, vitamin B6 (mg) including supplements, vitamin B12 (µg) including supplements, vitamin C (mg) including supplements, iodine (µg) including supplements, selenium (µg) including supplements); region: country people live; NumChild: number of children aged between 0 and 15; age: age of respondent 16 + ; SalHowC: how often salt added during cooking; Quarter: fieldwork quarter; qual7: qualifications gained; WrkStat: economic status (working condition); ethgrp5: ethnic group; eqv3: equivalized household income; HessCon: whether have any physical/mental health condition/illnesses for 12 months or more; Diabetes.combined: whether respondent is diabetic; cigsta3: cigarette Smoking Status; dnoft: frequency of alcohol consumption in past 12 months (including non-drinkers); bmival: BMI (kg/m^2^); whgval: waist-hip ratio groups

Model 1: crude with meanPulse only (univariate model)

Model 2: full model, adjusted by E-iAs_ing,water_, E-iAs_ing,grain_, age, bmival, cigsta3, region, Diabetes.combined, dnoft, eqv3, ethgrp5, SalHowC, HessCon, MN, NumChild, qual7, Quarter, Sex, surveyyr, whgval, WrkStat, EnergyDkJ, ProteingD, FatgD, GlucosegD, SodiummgD, FolateugplussuppsD

Model 3: adjusted by variables with *p* value lower than 0.2 in the univariate analysis E-iAs_ing,water_, E-iAs_ing,grain_, bmival, cigsta3, region, Diabetes.combined, dnoft, ethgrp5, MN, qual7, Sex, whgval, WrkStat, ProteingD, FatgD, GlucosegD, FolateugplussuppsD

Model 4: constructed by ‘stepwise’ function in R language based on AIC values which were adjusted by whgval, Sex, FolateugplussuppsD, bmival, MN, cigsta3, FatgD

*Compared with Quartile 1 (referent group) (0.00–0.00 µg/person/day)

**Table 11:** Modelling analysis of the categorical and continuous association of the odds ratio of general hypertension with E-iAs_ing,rice_ (µg/person/day). Data from NDNS RP 7–8 (MRC Elsie Widdowson Laboratory and NatCen Social Research 2019) with exclusion as detailed in the text (*N* = 598)

Model	Quartile of E-iAs_ing,rice_ (µg/person/day)							Odds ratio of general hypertension per 1 µg/person/day increase of E-iAs_ing,rice_	*p* value for trend	AIC	Contributions (%)
	Quartile 1 (0.00–0.00)	Quartile 2 (0.00–0.565)	*p* value*	Quartile 3 (0.638–3.79)	*p* value*	Quartile 4 (3.79–41.8)	*p* value*				
Model 1	1 (Referent)	0.61 (0.37, 0.98)	0.043	0.57 (0.35, 0.92)	0.023	0.44 (0.26, 0.72)	0.001	0.95 (0.91, 0.99)	0.023	727.0	0.7
Model 2	1 (Referent)	0.50 (0.22, 1.11)	0.088	0.55 (0.27, 1.09)	0.090	0.30 (0.14, 0.62)	0.002	0.94 (0.87, 1.00)	0.047	631.8	31.6
Model 3	1 (Referent)	0.69 (0.38, 1.26)	0.229	0.69 (0.38, 1.26)	0.227	0.50 (0.27, 0.93)	0.030	0.97 (0.92, 1.03)	0.364	586.7	27.7
Model 4	1 (Referent)	0.76 (0.41, 1.28)	0.270	0.75 (0.42, 1.34)	0.333	0.54 (0.30, 0.98)	0.043	0.98 (0.92, 1.03)	0.413	565.5	25.1
Nonlinear model	General hypertension ~ E-iAs_ing,rice_ + E-iAs_ing,rice_^2^ + Age + bmival + Diabetes.combined + HessCon								0.429	566.9	25.2

The differences of odds ratio of general hypertension across four quartiles were obtained from Wald tests for E-iAs_ing,rice_ coefficients, and the *p* value for linear and nonlinear trends was obtained from an analysis of variance (ANOVA) with type II error where E-iAs_ing,rice_ is a continuous measure of intake

general hypertension: whether participants were diagnosed as general hypertension; E-iAs_ing,rice_: daily inorganic arsenic (iAs) intake from rice and rice products; E-iAs_ing,water_: daily iAs intake from drinking water; E-iAs_ing,grain_: daily iAs intake from grain and grain-based products; surveyyr: NDNS RP 7–8 Survey year; Sex: gender; EnergyDkJ: intake of total energy per day (KJ) for diet only; ProteingD: intake of protein per day (g) for diet only; FatgD: intake of fat per day (g) for diet only; GlucosegD: intake of glucose per day (g) for diet only; SodiummgD: intake of sodium per day (mg) for diet only; FolateugplussuppsD: intake of folate (µg) per day for both diets and supplements; MN: daily intake of several micro-nutrients (Potassium (mg) including supplements, calcium (mg) including supplements, magnesium (mg) including supplements, iron (mg) including supplements, copper (mg) including supplements, zinc (mg) including supplements, retinol (mg) including supplements, vitamin A (retinol equivalents) (µg) including supplements, vitamin D (µg) including supplements, vitamin E (mg) including supplements, thiamin (mg) including supplements, riboflavin (mg) including supplements, niacin equivalent (mg) including supplements, vitamin B6 (mg) including supplements, vitamin B12 (µg) including supplements, vitamin C (mg) including supplements, iodine (µg) including supplements, selenium (µg) including supplements); region: country people live; NumChild: number of children aged between 0 and 15; age: age of respondent 16 + ; SalHowC: how often salt added during cooking; Quarter: fieldwork quarter; qual7: qualifications gained; WrkStat: economic status (working condition); ethgrp5: ethnic group; eqv3: equivalized household income; HessCon: whether have any physical/mental health condition/illnesses for 12 months or more; Diabetes.combined: whether respondent is diabetic; cigsta3: cigarette smoking status; dnoft: frequency of alcohol consumption in past 12 months (including non-drinkers); bmival: BMI (kg/m^2^); whgval: waist-hip ratio groups

Model 1: crude with the odds ratio of general hypertension only (univariate model)

Model 2: full model, adjusted by E-iAs_ing,water_, E-iAs_ing,grain_, age, bmival, cigsta3, region, Diabetes.combined, dnoft, eqv3, ethgrp5, SalHowC, HessCon, MN, NumChild, qual7, Quarter, Sex, surveyyr, whgval, WrkStat, EnergyDkJ, ProteingD, FatgD, GlucosegD, SodiummgD, FolateugplussuppsD

Model 3: adjusted by variables with *p* value lower than 0.2 in the univariate analysis E-iAs_ing,water_, E-iAs_ing,grain_, age, bmival, cigsta3, Diabetes.combined, dnoft, eqv3, HessCon, NumChild, qual7, whgval, WrkStat

Model 4: constructed by ‘stepwise’ function in R language based on AIC values which were adjusted by age, bmival, Diabetes.combined, HessCon

*Compared with Quartile 1 (referent group) (0.00–0.00 µg/person/day)

When E-iAs_ing,rice_ have been divided into 15 groups, more complex dose–response relationships between E-iAs_ing,rice_ and hypertension risks became evident (Table S4). Though not significant, higher hypertension risks could be found for some subgroups which could be supported by the role of As in inducing oxidative stress and altering the release of vasoactive mediators in blood vessel (Cifuentes et al. 2009). However, as no consistent dose–response patterns presented (Table S4), such higher risks may be due, at least in part, to just randomness or small sample sizes, indicating that the overall associations between E-iAs_ing,rice_ and hypertension were not strong at all.

Modification effects of several well-established risk factors for the relationships between E-iAs_ing,rice_ and hypertension risks.

The associations between E-iAs_ing,rice_ and hypertension risks estimated in the subgroup analysis were somewhat consistent across most of the subgroups by participants characteristics. However, higher DBP add 10 could be observed among participants with alcohol consumption once or twice a week. Similarly, there was higher risk among mixed ethnic group on the changes of SBP add 10 (Fig. S1–S4). In dose–response analysis, the adverse associations of iAs on the odds ratios of general hypertension were more apparent among participants who are male, aged between 35 and 49, overweight, or alcohol consumer when compared with their accordingly counterparts. To be noted, Asian or Asian British, Black or Black British and mixed ethnic group were found to be more vulnerable to the effects of iAs on the risks of general hypertension when compared with their White counterparts (Fig. S5).

## Discussion

This cross-sectional study conducted across four countries of the UK indicated negative but not significant associations between E-iAs_ing,rice_ and hypertension risks (DBP add 10, SBP add 10, AP, meanPulse and general hypertension), with relatively higher risks being found among subgroups who are male, aged between 35 and 49, overweight, alcohol consumers or belonging to Asian or Asian British, Black or Black British and mixed ethnic group when compared with their counterparts (Table 6, 7, 8, 9, 10, 11 and Table S4 and Fig. S1–S5). Though exploratory, our study was the first bridging the gap, at least partly, between individual level iAs intake from rice and rice products and hypertension risks, being important especially in areas where there is little exposure from drinking water but an increasing rice intake. Given the model uncertainties, including the fact that iAs in water or foods were not measured directly, low sample size in some stratified groups, the intrinsic shortages of cross-sectional studies and those limiting extrapolation to other potential confounders, the present study was still inconclusive and further model exploration as well as larger scale cohort studies are required (cf. Moon et al. 2013).

Combining the contributions of different factors to the variability of blood pressure endpoints estimated in the present study (Table 4) with the fact that a number of factors are widely known to be important indicators of hypertension risks, such as age, obesity, gender, smoking status, alcohol consumption and sodium intake (Biino et al. 2013; He et al. 2018; NHLBI Obesity Education Initiative Expert Panel on the Identification Evaluation and Treatment of Obesity in Adults (US) 1998), it should be acknowledged that E-iAs_ing,rice_ is likely to be of much lesser importance than other factors. Therefore, this study, not surprisingly, found only weak and not significant associations between E-iAs_ing,rice_ and hypertension risks (DBP add 10, SBP add 10, AP, meanPulse and general hypertension) (Table 6, 7, 8, 9, 10, 11 and Table S4).

The present study is somewhat consistent with previous research on the association of low-level As exposure from drinking water on hypertension risks which are largely inconclusive (Navas-Acien et al. 2006, 2019; Tsuji et al. 2014). For example, a cross-sectional study from Bangladesh, though revealing an adverse effect of low to moderate level As exposure (< 8 to 864 µg/L) on pulse pressure, only showed weak or no apparent associations for general, systolic or diastolic hypertension (Chen et al. 2007). Similarly, although it has been indicated that respondents exposed to well-water As concentrations greater than 10 µg/L have higher blood pressure when compared with those exposed to well-water As concentrations less than 2 µg/L (Zierold et al. 2004), that analysis was based on self-reported outcome assessment which makes such a conclusion less robust. In addition, at the low to moderate As drinking water levels typical of much of the US population in the National Health and Nutrition Examination Survey, total As, total As minus arsenobetaine in urine were not found to be associated with the prevalence of hypertension, SBP or DBP levels (Jones et al. 2011). Moreover, in an experiment in cells in vitro, a low dose of As was even reported to have a protective effect against CVD related to oxidative stress (Snow et al. 2005). Therefore, given the fact that the exact mechanisms by which As affect hypertension risks are still not clear (Chen et al. 2007), the negative and insignificant associations estimated in our study might be a real one especially for such low doses. Nevertheless, a weak or even significant adverse effects of As that is impossible to detect with the method used on this secondary dataset still cannot be ruled out, suggesting larger scale cohort studies are needed to investigate the effect of low-level iAs intake from rice and rice products on the hypertension risks.

There are already several well-established risk factors for hypertension, ranging from smoking (Chen et al. 2004; Kim and Lee 2019), gender (Watanabe et al. 2001), age (Camici et al. 2009) to diabetes (Epstein 1997), obesity (Derosa and Chiarolanza 2005; Re 2009) and alcohol consumption (Klatsky 2003). Accordingly, we explored the modification effects on these factors from iAs intake using subgroup analysis. However, our subgroup analysis must be interpreted cautiously. Susceptibility to As toxicity may differ by gender (Hsieh et al. 2011), although some previous studies of As and CVD found no marked differences (Afridi et al. 2011; Medrano et al. 2010). In our study, we found higher hypertension risks among males, and this result is consistent with Watanabe et al. (2001) which indicated that males in rural Bangladeshi communities were more susceptible to chronic As toxicity than females. We assume that such higher risks among males are largely due to their lower endogenous production of choline, and thus lower As methylation ability and hence greater susceptibility to higher As toxicity: this might also be related to the role of sex hormones in As methylation (Derosa and Chiarolanza 2005; Kim and Lee 2019). Also, in this study, (1) people aged between 35 and 49 and (2) people who have consumed alcoholic drinks over the previous 1 year were more susceptible to risks of general hypertension when compared with their otherwise equivalent (in terms of iAs intake) counterparts. This could be explained by increased exposure to factors known to inhibit methylation, viz. alcohol consumption, smoking and exposure to environmental pollutants (Chen et al. 2004; Hertz-Picciotto 2001). In addition, it is worth noting that differences could be found in the association between iAs intake and the risks of general hypertension for different ethnicities. The higher risks in Asian or Asian British, Black or Black British and mixed ethnic groups than for Whites might be related to their higher E-iAs_ing,rice_, low sample size in some stratified groups (only 8 and 14 participants belong to mixed ethnicity and Black and Black British, respectively), or some other unadjusted influential factors especially ethnicity-related dietary (Sekikawa et al. 2008; Tada et al. 2011) and genetic factors (Miller et al. 2004; Tanus-Santos et al. 2001), all of which may well be indistinguishable in their impacts from those of either iAs.

There are further limitations to this study including uncertainties in the estimation of iAs intake and blood pressure measurements: these errors may have weakened or even masked a possible underlying dose–response association. These errors include: (1) iAs concentration of different foodstuffs were not evaluated directly in the NDNS RP 7–8. In this study, instead, the European Food Safety Authority (2014) report was used to estimate mean iAs concentration in each foodstuff, leading to potential inaccuracies in our estimation of iAs intake, and thus in the modelled associations. (2) Dietary intakes were quantified by a food diary (MRC Elsie Widdowson Laboratory and NatCen Social Research 2019), and therefore measurements error are expected as the days selected for the dietary record might not represent their long-term dietary pattern and the weight of different foodstuffs estimated by participants themselves might not be precise; (3) Casual blood pressure readings cannot wholly represent the entire 24-h pattern, although there is no evidence of any systematic measurement errors in blood pressure measurements and the consistency between consecutive measurements was good (MRC Elsie Widdowson Laboratory and NatCen Social Research 2019). Also, the observed relationship between blood pressure and widely accepted major risk factors were in agreement with those of previously reported (Epstein 1997; Klatsky 2003; Mohtasham-Amiri et al. 2018; Neaton and Wentworth 1992; Owolabi et al. 2016), further suggesting the validity of the blood pressure measurements in this study.

Limitations could also exist in terms of the residual confounding issues. Some variables, ranging from age (Biino et al. 2013), diabetes (Epstein 1997), smoking (Chen et al. 2004; Kim and Lee 2019), obesity (Derosa and Chiarolanza 2005; Re 2009), household income (Mohtasham-Amiri et al. 2018; Owolabi et al. 2016) to education level (Cirera et al. 1998), glucose concentration (Banda et al. 2010), salt consumption (Lelong et al. 2019) and some nutrients intake (Betts and Foote 1985; Chen et al. 2007; Jarrah et al. 2018) are well recognized as important predictors of either hypertension risks or As intake, most of which have already been accounted for. However, factors such as individual level genetic information (Gong and O’Bryant 2012; Hsieh et al. 2017), metabolic syndrome (Zamora-Kapoor et al. 2018), history of pre-eclampsia (Zamora-Kapoor et al. 2018) or hypercholesterolemia (Cappuccio et al. 2003), physical activity (Banda et al. 2010) and some dietary-related information, including fatty acid intake (Zhao et al. 2011) which might be protective against hypertension risks perhaps ideally should have been taken into consideration in the present study as well. For French adults, more than 1% of new cases of hypertension were attributable to low physical activity (Lelong et al. 2019). Also, it has been reported that 5–13% population attributable risks of hypertension were due to physical inactivity with 3–16% due to a low intake of fatty acid in Finland, Italy, the Netherlands, UK and USA (Geleijnse et al. 2005). Similarly, some genetic factors, such as Angiotensinogen M235T genotype (Sethi et al. 2003) and CYP2J2*7 genotype (King et al. 2005), have been regarded to be partially responsible for hypertension risks, Unfortunately, due to lack of data sources or too many missing data, these variables were not considered in the present modelling analysis, meaning that the lower hypertension risks observed in the present study might not be mainly due to higher E-iAs_ing,rice_ but rather due to one or more of those unadjusted protective confounders, whether behavioural, dietary or genetic. Thus, Japanese population who have higher consumption level of rice and rice products than most of the UK population but also have adequate marine-derived n-3 fatty acids in their diets generally have lower hypertension risks (Sekikawa et al. 2008; Tada et al. 2011). Similarly, ethnic minorities in the UK with higher rice consumption level but found to be less prevalent in hypercholesterolemia (Cappuccio et al. 2003) may also suffer lower hypertension risks.

In addition, there may be dietary iAs intake from other sources. According to some surveys conducted in European countries and a probabilistic exposure modelling analysis in the USA, vegetables, fruit, and some dairy products may also be important contributors to dietary iAs exposure, with vegetables even accounting for more than 20% of iAs exposure for the general US population (European Food Safety Authority 2014; Henderson et al. 2003; Xue et al. 2010). However, NDNS RP 7–8 indicated that those sources might not contribute too much to the dietary iAs intake in the UK (MRC Elsie Widdowson Laboratory and NatCen Social Research 2019) and thus might not confound our estimated associations substantially. Similarly, as seafood usually contains organoarsenic compounds such as arsenobetaine and arsenosugars, which can be transformed to toxic arsenic metabolites after storage and cooking, As intake from seafood should also be considered (Taylor et al. 2017). However, there is no strong evidence of human toxicity reported currently from such sources (Chen et al. 2010; Ferrante et al. 2019).

Moreover, due to the intrinsic characteristics of a cross-sectional design, the possibility of recall bias when collecting information such as the frequency of smoking and alcohol consumption during the previous one year cannot be excluded. In addition, whilst there have been biologically plausible mechanistic processes previously proposed to support a causal link between iAs exposure and hypertension risks (Lee et al. 2005), cross-sectional studies themselves do merely indicate an association rather than causality.

Unlike many studies using ecologic measures of iAs intake (Bulka et al. 2016; Han et al. 2009; Mahram et al. 2013), this study assessed food consumption levels and thus iAs intake individually, with blood pressure measured via a standardized protocol (MRC Elsie Widdowson Laboratory and NatCen Social Research 2019). Also, as it is population-based, this study considered participants selection of whom depended neither on iAs exposure nor blood pressure status. Given the high-quality data collection methods and rigorous laboratory methods of NDNS RP 7–8 (MRC Elsie Widdowson Laboratory and NatCen Social Research 2019), the quality of the data obtained from NDNS RP 7–8 is considered to be good.

Taken together, this was the first study quantifying the individual-level dose–response associations between iAs intake from rice and rice products and CVD health, being of importance especially in a country such as the UK where there is little exposure from drinking water. This exploratory study suggests a negative but not significant association between exposure to iAs through eating rice or rice products and hypertension risks. However, due to the above-mentioned limitations in the present study, the possibility of any small positive or even significantly positive associations that are impossible to detect within the current study design and dataset still cannot be eliminated. This study does, however, highlight the need for further research in the area of the association between iAs exposure from rice and rice products and CVD health outcomes. In particular, larger scale cohort studies involving with more statistical power are indicated for better assessing such effects.

## Electronic supplementary material

Below is the link to the electronic supplementary material.

Supplementary material 1 (DOCX 2687 kb)

## References

[CR1] Afridi HI, Kazi TG, Kazi N, Kandhro GA, Baig JA, Jamali MK (2011). Association of environmental toxic elements in biological samples of myocardial infarction patients at different stages. Biological Trace Element Research.

[CR2] Attorp A, Scott JE, Yew AC, Rhodes RE, Barr SI, Naylor PJ (2014). Associations between socioeconomic, parental and home environment factors and fruit and vegetable consumption of children in grades five and six in British Columbia, Canada. BMC Public Health.

[CR3] Awata H, Linder S, Mitchell LE, Delclos GL (2017). Association of dietary intake and biomarker levels of arsenic, cadmium, lead, and mercury among asian populations in the United States: NHANES 2011–2012. Environmental Health Perspectives.

[CR4] Bae HS, Kang IG, Lee SG, Eom SY, Kim YD, Oh SY (2017). Arsenic exposure and seafood intake in Korean adults. Human and Experimental Toxicology.

[CR5] Banda JA, Clouston K, Sui X, Hooker SP, Lee CD, Blair SN (2010). Protective health factors and incident hypertension in men. American Journal of Hypertension.

[CR6] Betts NM, Foote D (1985). Nutrient intake and hypertension risk factors among blue collar workers. Nutrition Reports International.

[CR7] Biino G, Parati G, Concas MP, Adamo M, Angius A, Vaccargiu S (2013). Environmental and genetic contribution to hypertension prevalence: Data from an epidemiological survey on Sardinian genetic isolates. PLoS ONE.

[CR8] Bjorndal KA, Schroeder BA, Foley AM, Witherington BE, Bresette M, Clark D (2013). Temporal, spatial, and body size effects on growth rates of loggerhead sea turtles (*Caretta caretta*) in the Northwest Atlantic. Marine Biology.

[CR9] Bozdogan H (1987). Model selection and Akaike’s information criterion (AIC): The general theory and its analytical extensions. Journal of Psychometrika.

[CR10] Bulka CM, Jones RM, Turyk ME, Stayner LT, Argos M (2016). Arsenic in drinking water and prostate cancer in Illinois counties: An ecologic study. Environmental Research.

[CR11] Bundschuh J, Nath B, Bhattacharya P, Liu CW, Armienta MA, López MVM (2012). Arsenic in the human food chain: The Latin American perspective. Science of the Total Environment.

[CR12] Burnham KP, Anderson DR (2002). Model selection and multimodal inference: A practical-theoretic approach.

[CR13] Camici GG, Sudano I, Noll G, Tanner FC, Luscher TF (2009). Molecular pathways of aging and hypertension. Current Opinion in Nephrology and Hypertension.

[CR14] Cappuccio FP, Barbato A, Kerry SM (2003). Hypertension, diabetes and cardiovascular risk in ethnic minorities in the UK. The British Journal of Diabetes & Vascular Disease.

[CR15] Chakraborti D, Singh SK, Rahman MM, Dutta RN, Mukherjee SC, Pati S (2018). Groundwater arsenic contamination in the Ganga river basin: A future health danger. International Journal of Environmental Research and Public Health.

[CR16] Chen B, Chou W, Chen W, Liao C (2010). Assessing the cancer risk associated with arsenic-contaminated seafood. Journal of Hazardous Materials.

[CR17] Chen Y, Factor-Litvak P, Howe GR, Graziano JH, Brandt-Rauf P, Parvez F (2007). Arsenic exposure from drinking water, dietary intakes of B vitamins and folate, and risk of high blood pressure in Bangladesh: A population-based, cross-sectional study. American Journal of Epidemiology.

[CR18] Chen Y, Graziano JH, Parvez F, Liu ML, Slavkovich V, Kalra T (2011). Arsenic exposure from drinking water and mortality from cardiovascular disease in Bangladesh: Prospective cohort study. British Medical Journal.

[CR19] Chen CL, Hsu LI, Chiou HY, Hsueh YM, Chen SY, Wu MM (2004). Ingested arsenic, cigarette smoking, and lung cancer risk—A follow-up study in arseniasis-endemic areas in Taiwan. JAMA.

[CR20] Cifuentes F, Bravo J, Norambuena M, Stegen S, Ayavire A, Palacios J (2009). Chronic exposure to arsenic in tap water reduces acetylcholine-induced relaxation in the aorta and increases oxidative stress in female rats. International Journal of Toxicology.

[CR21] Cirera L, Tormo MJ, Chirlaque MD, Navarro C (1998). cardiovascular risk factors and educational attainment in Southern Spain: A study of a random sample of 3091 adults. European Journal of Epidemiology.

[CR22] Cleland B, Tsuchiya A, Kalman DA, Dills R, Burbacher TM, White JW (2009). Arsenic exposure within the Korean Community (United States) Based on dietary behavior and arsenic levels in hair, urine, air, and water. Environmental Health Perspectives.

[CR23] Collins R, MacMahon S (1994). Blood pressure, antihypertensive drug treatment and the risks of stroke and of coronary heart disease. British Medical Bulletin.

[CR24] Currie A (1947). The role of arsenic in carcinogenesis. British Medical Bulletin.

[CR25] Currier JM, Ishida MC, Gonzalez-Horta C, Sanchez-Ramirez B, Ballinas-Casarrubias L, Gutierrez-Torres DS (2014). Associations between arsenic species in exfoliated urothelial cells and prevalence of diabetes among residents of Chihuahua, Mexico. Environmental Health Perspectives.

[CR26] Derosa ML, Chiarolanza C (2005). Obesity, weight loss and hypertension. American Journal of Hypertension.

[CR27] Diane GD, Li Z, Perry AE, Spencer SK, Jay GA, Karagas MR (2013). A population-based case-control study of urinary arsenic species and squamous cell carcinoma in New Hampshire, USA. Environmental Health Perspectives.

[CR28] Dt. Gesellschaft für Sportmedizin und Prävention e.V. (DGSP). (2007). *S 1*-*Leitlinie Vorsorgeuntersuchung im Sport*. German.

[CR29] El-Masri HA, Hong T, Henning C, Mendez WJ, Hudgens EE, Thomas DJ (2018). Evaluation of a physiologically based pharmacokinetic (PBPK) model for inorganic arsenic exposure using data from two diverse human populations. Environmental Health Perspectives.

[CR30] Epstein M (1997). Diabetes and hypertension: The bad companions. Journal of Hypertension.

[CR31] European Food Safety Authority (2009). Scientific opinion on arsenic in food.

[CR32] European Food Safety Authority (2014). Scientific opinion on dietary exposure to inorganic arsenic in the European population.

[CR33] Ferguson TS, Younger-Coleman NOM, Tulloch-Reid MK, Bennett NR, Rousseau AE, Knight-Madden JM (2018). Factors associated with elevated blood pressure or hypertension in Afro-Caribbean youth: A cross-sectional study. PeerJ.

[CR34] Ferrante M, Napoli S, Grasso A, Zuccarello P, Cristaldi A, Copat C (2019). Systematic review of arsenic in fresh seafood from the Mediterranean Sea and European Atlantic coasts: A health risk assessment. Food and Chemical Toxicology.

[CR35] Food and Agriculture Organization of the United Nations (2008). Rice market monitor.

[CR36] Frost DV (1969). Arsenic and cancer [Letter]. Journal of Allergy.

[CR37] Gao YF, Zhao ZQ, Yang LQ, Liu XX, Xing XM, Zhang HM (2018). Arsenic exposure assists ccm3 genetic polymorphism in elevating blood pressure. Oncotarget.

[CR38] Geleijnse JM, Grobbee DE, Kok FJ (2005). Impact of dietary and lifestyle factors on the prevalence of hypertension in Western populations. Journal of Human Hypertension.

[CR39] Gong G, O’Bryant SE (2012). Low-level arsenic exposure, AS3MT gene polymorphism and cardiovascular diseases in rural Texas counties. Environmental Research.

[CR40] Gossai A, Zens MS, Punshon T, Jackson BP, Perry AE, Karagas MR (2017). Rice consumption and squamous cell carcinoma of the skin in a United States population. Environmental Health Perspectives.

[CR41] Hall EM, Acevedo J, López FG, Cortés S, Ferreccio C, Smith AH (2017). Hypertension among adults exposed to drinking water arsenic in Northern Chile. Environmental Research.

[CR42] Han YY, Weissfeld JL, Davis DL, Talbott EO (2009). Arsenic levels in ground water and cancer incidence in Idaho: An ecologic study. International Archives of Occupational and Environmental Health.

[CR43] He X, Li Z, Tang X, Zhang L, Wang L, He Y (2018). Age- and sex-related differences in body composition in healthy subjects aged 18 to 82 years. Medicine.

[CR44] Henderson L, Gregory J, Swan G (2003). The national diet and nutrition survey: Adults aged 19 to 64 years.

[CR45] Hertz-Picciotto I (2001). Interactions between arsenic and other factors in relation to carcinogenicity (Arsenic exposure and health effects).

[CR46] Hossain K, Suzuki T, Hasibuzzaman MM, Islam MS, Rahman A, Paul SK (2017). Chronic exposure to arsenic, LINE-1 hypomethylation, and blood pressure: A cross-sectional study in Bangladesh. Environmental Health.

[CR47] Hsieh FI, Huang JY, Chiou HY (2017). Association of genetic polymorphisms of AS3MT and N6AMT1 with the risk of arsenic-related cardiovascular disease. Atherosclerosis.

[CR48] Hsieh YC, Lien LM, Chung WT, Hsieh FI, Hsieh PF, Wu MM (2011). Significantly increased risk of carotid atherosclerosis with arsenic exposure and polymorphisms in arsenic metabolism genes. Environmental Research.

[CR49] Hu H, Balakrishnan K (2005). The environment & health: An emerging area of research in India. Indian Journal of Medical Research.

[CR50] Huda N, Hossain S, Rahman M, Karim MR, Islam K, Mamun AA (2014). Elevated levels of plasma uric acid and its relation to hypertension in arsenic-endemic human individuals in Bangladesh. Toxicology and Applied Pharmacology.

[CR51] Hueper W, Truhaut R (1967). Carcinogenic hazards from arsenic and metal containing drugs. Potential carcinogenic hazards from drugs.

[CR52] Islam S, Rahman MM, Rahman MA, Naidu R (2017). Inorganic arsenic in rice and rice-based diets: Health risk assessment. Food Control.

[CR53] Jarrah MI, Mhaidat NM, Alzoubi KH, Alrabadi N, Alsatari E, Khader Y (2018). The association between the serum level of vitamin D and ischemic heart disease: A study from Jordan. Vascular Health and Risk Management.

[CR54] Jones MR, Tellezplaza M, Sharrett AR, Guallar E, Navasacien A (2011). Urine arsenic and hypertension in us adults: The 2003–2008 NHANES. Epidemiology.

[CR55] Kannel WB (1996). Blood pressure as a cardiovascular risk factor: Prevention and treatment. JAMA.

[CR56] Kim SH, Lee JS (2019). The association of smoking and hypertension according to cotinine-verified smoking status in 25,150 Korean adults. Clinical and Experimental Hypertension.

[CR57] King LM, Gainer JV, David GL, Dai D, Goldstein JA, Brown NJ (2005). Single nucleotide polymorphisms in the CYP2J2 and CYP2C8 genes and the risk of hypertension. Pharmacogenetics and Genomics.

[CR58] Klatsky AL (2003). Alcohol and hypertension: Does it matter? Yes. Journal of Cardiovascular Risk.

[CR59] Kunrath J, Gurzau E, Gurzau A, Goessler W, Gelmann ER, Thach TT (2013). Blood pressure hyperreactivity: An early cardiovascular risk in normotensive men exposed to low-to-moderate inorganic arsenic in drinking water. Journal of Hypertension.

[CR60] Lee MY, Lee YH, Lim KM, Chung SM, Bae ON, Kim H (2005). Inorganic arsenite potentiates vasoconstriction through calcium sensitization in vascular smooth muscle. Environmental Health Perspectives.

[CR61] Lelong H, Blacher J, Baudry J, Adriouch S, Galan P, Fezeu L (2019). Combination of healthy lifestyle factors on the risk of hypertension in a large cohort of French adults. Nutrients.

[CR62] Li Y, Wang D, Li X, Zheng Q, Sun G (2015). A potential synergy between incomplete arsenic methylation capacity and demographic characteristics on the risk of hypertension: Findings from a cross-sectional study in an arsenic-endemic area of Inner Mongolia, China. International Journal of Environmental Research and Public Health.

[CR63] Mahram M, Shahsavari D, Oveisi S, Jalilolghadr S (2013). Comparison of hypertension and diabetes mellitus prevalence in areas with and without water arsenic contamination. Journal of Research in Medical Sciences.

[CR64] Mantha M, Yeary E, Trent J, Creed PA, Kubachka K, Hanley T (2017). Estimating inorganic arsenic exposure from US rice and total water intakes. Environmental Health Perspectives.

[CR65] McCarty KM, Hanh HT, Kim KW (2011). Arsenic geochemistry and human health in South East Asia. Reviews on Environmental Health.

[CR66] Medrano MJ, Boix R, Pastor-Barriuso R, Palau M, Damian J, Ramis R (2010). Arsenic in public water supplies and cardiovascular mortality in Spain. Environmental Research.

[CR67] Meharg AA, Adomaco E, Lawgali Y, Deacon C, Williams P (2007). Food Standards Agency contract C101045: Levels of arsenic in rice-literature review.

[CR68] Meharg AA, Lombi E, Williams PN, Scheckel KG, Feldmann J, Raab A (2008). Speciation and localization of arsenic in white and brown rice grains. Environmental Science and Technology.

[CR69] Meharg AA, Zhao FJ (2012). Arsenic & rice.

[CR70] Miller MA, Kerry SM, Dong YB, Strazzullo P, Cappuccio FR (2004). Association between the Thr715Pro P-selectin gene polymorphism and soluble P-selectin levels in a multiethnic population in South London. Thrombosis and Haemostasis.

[CR71] Mohtasham-Amiri Z, Rezvani M, Jafari-Shakib A, Ebrahimifar H, Esmaeilpour J, Mohtasham-Amiri Z (2018). Hypertension in the lowest decile income population of Guilan, North of Iran. Journal of Hypertension.

[CR72] Molin M, Ulven SM, Meltzer HM, Alexander J (2015). Arsenic in the human food chain, biotransformation and toxicology—Review focusing on seafood arsenic. Journal of Trace Elements in Medicine and Biology.

[CR73] Mondal D, Polya DA (2008). Rice is a major exposure route for arsenic in Chakdaha block, Nadia district, West Bengal, India: A probabilistic risk assessment. Applied Geochemistry.

[CR74] Moon KA, Guallar E, Umans JG, Devereux RB, Best LG, Francesconi KA (2013). Association between exposure to low to moderate arsenic levels and incident cardiovascular disease. Annals of Internal Medicine.

[CR75] Mordukhovich I, Wright RO, Hu H, Amarasiriwardena C, Baccarelli A, Litonjua A (2012). Associations of toenail arsenic, cadmium, mercury, manganese, and lead with blood pressure in the normative aging study. Environmental Health Perspectives.

[CR76] MRC Elsie Widdowson Laboratory, & NatCen Social Research (2019). National diet and nutrition survey years 1–9, 2008/09–2016/17.

[CR77] Mwale T, Rahman MM, Mondal D (2018). Risk and benefit of different cooking methods on essential elements and arsenic in rice. International Journal of Environmental Research & Public Health.

[CR78] Navas-Acien A, Sanchez TR, Mann K, Jones MR (2019). Arsenic exposure and cardiovascular disease: evidence needed to inform the dose–response at low levels. Current Epidemiology Reports.

[CR79] Navas-Acien A, Sharrett AR, Guallar E (2006). Arsenic exposure and cardiovascular disease: A systematic review of the epidemiologic evidence. American Journal of Epidemiology.

[CR80] Neaton JD, Wentworth D (1992). Serum cholesterol, blood pressure, cigarette smoking, and death from coronary heart disease overall findings and differences by age for 316099 white men. Archives of Internal Medicine.

[CR81] NHLBI Obesity Education Initiative Expert Panel on the Identification Evaluation and Treatment of Obesity in Adults (1998). Clinical guidelines on the identification, evaluation and treatment of overweight and obesity in adults: The evidence report.

[CR82] Owolabi M, Olowoyo P, Miranda JJ, Akinyemi R, Feng WW, Yaria J (2016). Gaps in hypertension guidelines in low- and middle-income versus high-income countries: A systematic review. Hypertension.

[CR83] Polya DA, Lawson M, States JC (2016). Geogenic and anthropogenic arsenic hazard in groundwaters and soils. Arsenic: Exposure sources, health risks, and mechanisms of toxicity.

[CR84] Polya DA, Middleton D, Bhattacharya P, Polya DA, Jovanovic D (2017). Arsenic in drinking water: Sources & human exposure. Best practice guide on the control of arsenic in drinking water.

[CR85] Rahman M (2002). Arsenic and hypertension in Bangladesh. Bulletin of the World Health Organization.

[CR86] Rahman M, Sohel N, Yunus M, Chowdhury ME, Hore SK, Zaman K (2014). A prospective cohort study of stroke mortality and arsenic in drinking water in Bangladeshi adults. BMC Public Health.

[CR87] Re RN (2009). Obesity-related hypertension. Ochsner Journal.

[CR88] Schenker S (2012). An overview of the role of rice in the UK diet. Nutrition Bulletin.

[CR89] Schoof RA, Eickhoff J, Yost LJ, Crecelius EA, Cragin DW, Meacher DM, Chappell WR, Abernathy CO, Calderon RL (1999). Dietary exposure to inorganic arsenic. Arsenic exposure and health effects III.

[CR90] Sekikawa A, Curb JD, Ueshima H, El-Saed A, Kadowaki T, Abbott RD (2008). Marine-derived n-3 fatty acids and atherosclerosis in Japanese, Japanese-American, and White men: A cross-sectional study. Journal of the American College of Cardiology.

[CR91] Sethi AA, Nordestgaard BG, Tybjærg-Hansen A (2003). Angiotensinogen gene polymorphism, plasma angiotensinogen, and risk of hypertension and ischemic heart disease: A meta-analysis. Arteriosclerosis, Thrombosis, and Vascular Biology.

[CR92] Signes-Pastor AJ, Vioque J, Navarrete-Muñoz EM, Carey M, Hera MG, de la Sunyer J (2017). Concentrations of urinary arsenic species in relation to rice and seafood consumption among children living in Spain. Environmental Research.

[CR93] Snow ET, Sykora P, Durham TR, Klein CB (2005). Arsenic, mode of action at biologically plausible low doses: What are the implications for low dose cancer risk?. Toxicology and Applied Pharmacology.

[CR94] Sowers JR, Epstein M, Frohlich ED (2001). Diabetes, hypertension, and cardiovascular disease. Hypertension.

[CR95] Stamler J, Stamler R, Neaton JD (1993). Blood pressure, systolic and diastolic, and cardiovascular risks: US population data. Archives of Internal Medicine.

[CR96] Steinmaus C, Castriota F, Ferreccio C, Smith AH, Yuan Y, Liaw J (2015). Obesity and excess weight in early adulthood and high risks of arsenic-related cancer in later life. Environmental Research.

[CR97] Tada N, Maruyama C, Koba S, Tanaka H, Birou S, Teramoto T (2011). Japanese dietary lifestyle and cardiovascular disease. Journal of Atherosclerosis & Thrombosis.

[CR98] Tanus-Santos JE, Desai M, Flockhart DA (2001). Effects of ethnicity on the distribution of clinically relevant endothelial nitric oxide variants. Pharmacogenetics.

[CR99] Taylor V, Goodale B, Raab A, Schwerdtle T, Reimer K, Conklin S (2017). Human exposure to organic arsenic species from seafood. Science of the Total Environment.

[CR100] Torres-Escribano S, Leal M, Vélez D, Montoro R (2008). Total and inorganic arsenic concentrations in rice sold in Spain, effect of cooking, and risk assessments. Environmental Science and Technology.

[CR101] Tsuji JS, Perez V, Garry MR, Alexander DD (2014). Association of low-level arsenic exposure in drinking water with cardiovascular disease: A systematic review and risk assessment. Toxicology.

[CR102] US Department of Health And Human Services, Public Health Service, & Agency for Toxic Substances and Disease Registry (ATSDR) (2007). Toxicological profile for arsenic.

[CR103] Wang SL, Li WF, Chen CJ, Huang YL, Chen JW, Chang KH (2011). Hypertension incidence after tap-water implementation: A 13-year follow-up study in the arseniasis-endemic area of southwestern Taiwan. Science of the Total Environment.

[CR104] Watanabe C, Inaoka T, Kadono T, Nagano M, Nakamura S, Ushijima K (2001). Males in rural Bangladeshi communities are more susceptible to chronic arsenic poisoning than females: Analyses based on urinary arsenic. Environmental Health Perspectives.

[CR105] World Health Organization (2011). Global status report on noncommunicable diseases 2010.

[CR106] Wu F, Jasmine F, Kibriya MG, Liu ML, Wojcik O, Parvez F (2012). Association between arsenic exposure from drinking water and plasma levels of cardiovascular markers. American Journal of Epidemiology.

[CR107] Xia Y, Liu J (2004). An overview on chronic arsenism via drinking water in PR China. Toxicology.

[CR108] Xue J, Zartarian V, Wang SW, Liu SV, Georgopoulos P (2010). Probabilistic modeling of dietary arsenic exposure and dose and evaluation with 2003–2004 NHANES data. Environmental Health Perspectives.

[CR109] Yáñez J, Mansilla HD, Santander IP, Fierro V, Cornejo L, Barnes RM (2015). Urinary arsenic speciation profile in ethnic group of the Atacama desert (Chile) exposed to variable arsenic levels in drinking water. Journal of Environmental Science & Health Part A.

[CR110] Yoder SR, Thornburg LL, Bisognano JD (2009). Hypertension in pregnancy and women of childbearing age. The American Journal of Medicine.

[CR111] Zamora-Kapoor A, Sinclair K, Walker-Harding L, Schwartz S, Umans J, Buchwald D (2018). Risk and protective factors for hypertension in American Indian and Alaska native adolescents and adults: A systematic review. Journal of Adolescent Health.

[CR112] Zhao D, Qi Y, Zheng Z, Wang Y, Zhang XY, Li HJ (2011). Dietary factors associated with hypertension. Nature Reviews Cardiology.

[CR113] Zierold KM, Knobeloch L, Anderson H (2004). Prevalence of chronic diseases in adults exposed to arsenic-contaminated drinking water. American Journal of Public Health.

