# Supplementary material for: Exploratory study of the association in the United Kingdom between hypertension and inorganic arsenic (iAs) intake from rice and rice products
Source: Environ Geochem Health. 2020 Apr 28;43(7):2505–38. doi: 10.1007/s10653-020-00573-8 (PMC8275557; doi:10.1007/s10653-020-00573-8)
Supplement: Supplementary file 1 — Supplementary material 1 (DOCX 2687 kb) [file 10653_2020_573_MOESM1_ESM.docx]

Supplementary information for

Exploratory study of the association in the United Kingdom between hypertension and inorganic arsenic (iAs) intake from rice and rice products

Lingqian Xu^1^ and David A. Polya^1^*

^1^Department of Earth and Environmental Sciences and Williamson Research Centre for Molecular Environmental Science, University of Manchester, Manchester, M13 9PL, UK

Address correspondence to: David A. Polya, Department of Earth and Environmental Sciences and Williamson Research Centre for Molecular Environmental Science, University of Manchester, Manchester, M13 9PL, UK; Telephone: +44 (161) 275 38181; E-mail: david.polya@manchester.ac.uk. The study was jointly conceived by LX and DP and executed by LX under the supervision of DP. Both authors contributed to the writing and revision of the manuscript.

**Table S1** Detailed description of variables included in the present study. Data from all the participants recruited in the NDNS RP 7-8 (N = 2723) (MRC Elsie Widdowson Laboratory and NatCen Social Research 2019)

| Variable name/code | Description | Original grouping | Regrouping/Calculation | Variable type |
| --- | --- | --- | --- | --- |
| age | Age of respondent 16+ year | 16-18; 19-34; 35-49; 50-64; 65+ years | Regrouping: People lower than 16 have been excluded; then regrouping: 16-34; 35-49; 50-64; 65+ years | Categorical |
| AP | Mean arterial pressure (mmHg) | Continuous | Calculation: 1/3×(SBP add 10+2×DBP add 10) | Continuous |
| bmival | Valid BMI (kg/m^2^) | Underweight: Under 18.5; Normal (healthy weight): 18.5 and below 25; Overweight: 25 and below 30; Obese Class I (Moderately)-II (Severely): 30 and below 40; Obese Class III (Very severely obese): over 40 | Regrouping: Under 18.5 (underweight); 18.5 and below 25 (normal); 25 and below 30 (overweight); Over 30 (obese) | Categorical |
| cigsta3 | Cigarette Smoking Status | Current cigarette smoker; Ex-regular cigarette smoker; Never regular cigarette smoker |  | Categorical |
| DBP add 10 | Omron valid mean diastolic blood pressure (DBP) incremented by 10 mmHg is added if anti-hypertension medication is taken (mmHg) | Continuous | Calculation: People taking anti-hypertension medications have controlled and likely artificially low blood pressure. We addressed this by adding a constant (10 mmHg) to the SBP (SBP add 10) and DBP (DBP add 10) of the participants with anti-hypertension medications. | Continuous |
| Diabetes.combined | Whether respondent is diabetic | Not diabetics; Diabetics |  | Categorical |
| dnoft | Frequency of alcohol consumption in past 12 months (including non-drinkers) | Almost every day; Five or six days a week; Three or four days a week; Once or twice a week; Once or twice a month; Once every couple of months; Once or twice a year; Not at all in the last 12 months/Non-drinker | Regrouping: Five or seven days a week; Three or four days a week; Once or twice a week; Once or twice a month; Once every couple of months; Once or twice a year; Not at all in the last 12 months/Non-drinker | Categorical |
| E-iAs_ing,rice_ | Daily inorganic arsenic (iAs) intake from rice & rice products (µg/person/day) | Continuous | $\text{E-iAs}_{\text{ing,rice}}\text{=}\sum_{\text{i}} {\text{RC}_{\text{i}}\text{×C}}_{\text{rice,i}}\text{×(1-LOSS}_{\text{cooking}}\text{)}$  $\text{RC}_{\text{i}}\text{=}\frac{\sum_{\text{1}}^{\text{n}} \text{DRC}_{\text{i}}}{\text{n}}$  $\text{E-iAs}_{\text{ing,water}}\text{=}\text{C}_{\text{water}}\text{×WC}$  $\text{WC=}\frac{\sum_{\text{1}}^{\text{n}} \text{DWC}}{\text{n}}$  $\text{E-iAs}_{\text{ing,grain}}\text{=}\sum_{\text{i}} \text{C}_{\text{grain,i}}\text{×}\text{GC}_{\text{i}}$  $\text{GC}_{\text{i}}\text{=}\frac{\sum_{\text{1}}^{\text{n}} \text{DGC}_{\text{i}}}{\text{n}}$  where:  RC_i_ is the average daily consumption (kg/day) of rice and rice product, i, during the food diary (ready-to-eat)  C_rice,i_ is the iAs concentration (µg/kg) of the rice and rice product, i (ready-to-eat or raw)  LOSS_cooking_ is the estimated proportion of iAs lost from rice and rice products upon cooking  n: length of the food diary (three days (n=3) or four days (n=4))  DRC_i_: consumption rate (kg/day) of rice and rice product, i, in each day during the food diary  C_water_: iAs concentration (µg/L) in drinking water  WC: average daily intake (L/day) of drinking water during the food diary  DWC: consumption (L/day) of drinking water in each day during the food diary  C_grain,i_: iAs concentration (µg/kg) of the grain and grain-based product, i  GC_i_: average daily consumption (kg/day) of grain and grain-based product, i, during the food diary  DGC_i_: consumption rate (kg/day) of grain and grain-based product, i, in each day during the food diary | Continuous |
| E-iAs_ing,water_ | Daily iAs intake from drinking water (µg/person/day) |  |  |  |
| E-iAs_ing,grain_ | Daily iAs intake from grain & grain-based products (µg/person/day) |  |  |  |
| EnergyDkJ | Intake of total energy per day (KJ) for diet only | Continuous | Grouping: Quartile cutoffs were based on the distributions of energy intake level in the study population. 4 groups: (top 681 participants (Quartile 1) +681 participants (Quartile 2) +680 participants (Quartile 3) +681 participants (Quartile 4))  Ranges of EnergyDkJ: Q1: 189.46-1294.86; Q2: 1295.07-1627.87; Q3: 1628.14-2037.01; Q4: 2037.83-4771.28 | Categorical |
| eqv3 | Equivalised household income (£) | Lowest Tertile (≤ 17,500); Middle Tertile (> 17,500 ≤ 32,216); Highest Tertile (> 32,500) |  | Categorical |
| ethgrp5 | Ethnic group, 5 groups | White; Mixed ethnic group; Black or Black British; Asian or Asian British; Any other group |  | Categorical |
| FatgD | Intake of fat per day (g) for diet only | Continuous | Grouping: Quartile cutoffs were based on the distributions of the accordingly nutrient intake level in the study population. 4 groups: (top 681 participants (Quartile 1) +681 participants (Quartile 2) +680 participants (Quartile 3) +681 participants (Quartile 4))  Ranges of FatgD: Q1: 0.12- 10.64; Q2: 10.65-14.21; Q3: 14.23-18.71; Q4: 18.74-59.89  Ranges of FolateugplussuppsD: Q1: 5.69-35.36; Q2: 36.38-47.85; Q3: 47.87-65.81; Q4: 65.86-1426.32 | Categorical |
| FolateugplussuppsD | Intake of folate (µg) per day for both diets and supplements |  |  |  |
| general hypertension | Whether participants was diagnosed as general hypertension | 0 = without general hypertension; 1 = with general hypertension | General hypertension is defined as a systolic blood pressure ≥ 140 mmHg, or a diastolic blood pressure ≥ 90 mmHg and/or under regular treatment with anti-hypertension medications. | Categorical |
| GlucosegD | Intake of glucose per day (g) for diet only | Continuous | Grouping: Quartile cutoffs were based on the distributions of glucose intake level in the study population. 4 Grouping: (top 681 participants (Quartile 1) +681 participants (Quartile 2) +680 participants (Quartile 3) +681 participants (Quartile 4))  Ranges of GlucosegD: Q1: 0.06- 2.19; Q2: 2.20-3.34; Q3: 3.35-4.88; Q4: 4.89-30.28 | Categorical |
| HessCon | Whether have any physical/mental health condition/illnesses for 12 months or more | Yes; No |  | Categorical |
| meanPulse | Mean pulse pressure (mmHg) | Continuous | Calculation: Mean value of the three valid Pulse Pressure readings | Continuous |
| MN | Daily intake levels of several micro-nutrients (Potassium (mg) including supplements, Calcium (mg) including supplements, Magnesium (mg) including supplements, Iron (mg) including supplements, Copper (mg) including supplements, Zinc (mg) including supplements, Retinol (mg) including supplements, Vitamin A (retinol equivalents) (µg) including supplements, Vitamin D (µg) including supplements, Vitamin E (mg) including supplements, Thiamin (mg) including supplements, Riboflavin (mg) including supplements, Niacin equivalent (mg) including supplements, Vitamin B6 (mg) including supplements, Vitamin B12 (µg) including supplements, Vitamin C (mg) including supplements, Iodine (µg) including supplements, Selenium (µg) including supplements) | Continuous | Calculation: For all the micro-nutrients intake variables, a score of 0 or 1 was assigned to participants with less than (<) or greater than or equal to (≥) the mean intake level of each micro-nutrient, respectively. A composite measure was then created by summing the individual score.  Grouping: 1: participants with 0-3 nutrients ≥ the mean intake level of the accordingly nutrients; 2: participants with 4-7 nutrients ≥ the mean intake level of the accordingly nutrients; 3: participants with 8-11nutrients ≥ the mean intake level of the accordingly nutrients; 4: participants with 12-15 nutrients ≥ the mean intake level of the accordingly nutrients; 5: participants with 16-18 nutrients ≥ the mean intake level of the accordingly nutrients | Categorical |
| NumChild | Number of Children aged between 0 and 15 | Have 0-6 children | Regrouping: Have no child; Have 1-2 children; Have 3-4 children; Have 5-6 children | Categorical |
| ProteingD | Intake of protein per day (g) for diet only | Continuous | Grouping: Quartile cutoffs were based on the distributions of protein intake level in the study population. 4 groups: (top 681 participants (Quartile 1) +681 participants (Quartile 2) +680 participants (Quartile 3) +681 participants (Quartile 4))  Ranges of ProteingD: Q1: 1.17-11.90; Q2: 11.91-15.42; Q3: 15.43-19.31; Q4: 19.33- 69.00 | Categorical |
| qual7 | Qualifications gained, grouped | Degree or equivalent; Higher education, below degree level; GCE, A level or equivalent; GCSE grades A-C or equivalent; GCSE grades D-G/Commercial qualifications/apprenticeship; Foreign or other qualifications; No qualifications; Still in FT education | Regrouping:  Degree or equivalent; Higher education, below degree level & GCE, A level or equivalent; GCSE grades A-G or equivalent /Commercial qualifications/apprenticeship; Foreign or other qualifications & No qualifications & Still in FT education | Categorical |
| Quarter | Fieldwork quarter | Season 1: Apr - Jun; Season 2: Jul - Sep; Season 3: Oct - Dec; Season 4: Jan - Mar |  | Categorical |
| region | Country people live | England: Central/Midlands; England: North England: South (incl. London); Northern Ireland; Scotland | Regrouping: England: Central/Midlands; England: North England: South (including London); Northern Ireland & Scotland | Categorical |
| SalHowC | How often salt added during cooking | Never; Sometimes; Usually; Always | Regrouping: Never; Sometimes & Usually; Always | Categorical |
| SBP add 10 | Omron valid mean systolic blood pressure (SBP) incremented by 10 mmHg is added if anti-hypertension medication is taken (mmHg) | Continuous | Calculation: People taking anti-hypertension medications have controlled and likely artificially low blood pressure. We addressed this by adding a constant (10 mmHg) to the SBP (SBP add 10) for participants with anti-hypertension medications. | Continuous |
| Sex | Gender | Male; Female |  | Categorical |
| SodiummgD | Intake of sodium per day (mg) for diet only | Continuous | Grouping: Quartile cutoffs were based on the distributions of sodium intake level in the study population. 4 groups: (top 681 participants (Quartile 1) +681 participants (Quartile 2) +680 participants (Quartile 3) +681 participants (Quartile 4))  Ranges of SodiummgD: Q1: 22.97- 327.89; Q2: 327.91-430.23; Q3: 430.26-561.96; Q4: 562.28-2306.84 | Categorical |
| surveyyr | NDNS RP 7-8 Survey year | Year 7 (2014-2015); Year 8 (2015-2016) |  | Categorical |
| whgval | Waist-hip ratio groups | Continuous | Grouping:   \|  \| Women \| Men \| \| --- \| --- \| --- \| \| normal weight \| < 0.80 \| < 0.90 \| \| overweight \| 0.80-0.84 \| 0.90-0.99 \| \| [obesity](https://en.wikipedia.org/wiki/Obesity) \| > 0.85 \| > 1.00 \| | Categorical |
| WrkStat | Economic status (working condition) | Going to school or college full-time (including on vacation); In full or part-time employment; Not working at present | Regrouping: In full or part-time employment; Going to school or college full-time (including on vacation) & Not working at present | Categorical |

**Table S2** Characteristics of participants over 16 between included and excluded from the present study. Data from NDNS RP 7-8 (MRC Elsie Widdowson Laboratory and NatCen Social Research 2019) with participants over 16 (N = 1649)

|  | | Include | Exclude | p-value |
| --- | --- | --- | --- | --- |
| E-iAs_ing.rice_ (µg/person/day) | | 2.81 | 2.42 | 0.091 |
| NumChild | Have no child | 68.73% | 69.36% | 0.233 |
|  | Have 1-2 children | 27.76% | 25.59% |  |
|  | Have 3-4 children | 3.51% | 4.57% |  |
|  | Have 5-6 children | 0.00% | 0.48% |  |
| Sex | Male | 41.97% | 43.96% | 0.439 |
|  | Female | 58.03% | 56.04% |  |
| age | 16-34 | 25.92% | 37.77% | < 0.001 |
|  | 35-49 | 27.09% | 21.50% |  |
|  | 50-64 | 27.09% | 20.17% |  |
|  | 65+ | 19.90% | 20.55% |  |
| WrkStat | In full or part-time employment | 55.85% | 44.81% | < 0.001 |
|  | Going to school or college full-time (including on vacation) & Not working at present | 44.15% | 55.19% |  |
| HessCon | Have any physical/mental health condition/illnesses for 12 months or more | 36.29% | 37.58% | 0.633 |
|  | Without any physical/mental health condition/illnesses for 12 months or more | 63.71% | 62.42% |  |
| cigsta3 | Current cigarette smoker | 14.21% | 23.30% | < 0.001 |
|  | Ex-regular cigarette smoker | 23.75% | 20.33% |  |
|  | Never regular cigarette smoker | 62.04% | 56.38% |  |
| dnoft | ≥ Five days a week | 9.87% | 9.50% | 0.005 |
|  | Three or four days a week | 12.04% | 7.97% |  |
|  | Once or twice a week | 28.26% | 25.14% |  |
|  | Once or twice a month | 16.39% | 16.31% |  |
|  | Once every couple of months | 10.20% | 12.28% |  |
|  | Once or twice a year | 10.54% | 9.79% |  |
|  | Not at all in the last 12 months/Non-drinker | 12.71% | 19.00% |  |
| bmival (kg/m^2^) | Underweight: Under 18.5 | 2.34% | 2.66% | 0.306 |
|  | Normal (healthy weight): 18.5 and below 25 | 34.95% | 2.66% |  |
|  | Overweight: 25 and below 30 | 37.96% | 2.66% |  |
|  | Obese: over 30 | 24.75% | 2.66% |  |
| whgval | For male: less than 0.9; For female: less than 0.80 | 39.30% | 35.63% | 0.446 |
|  | For male: more than and including 0.90, up to and including 1.00; For female: more than and including 0.80, up to and including 0.85 | 27.93% | 29.13% |  |
|  | For male: more than 1.00; For female: more than 0.85 | 32.78% | 35.24% |  |
| ethgrp5 | White | 91.97% | 90.12% | 0.697 |
|  | Mixed ethnic group | 1.34% | 1.15% |  |
|  | Black or Black British | 2.34% | 2.88% |  |
|  | Asian or Asian British | 3.34% | 4.60% |  |
|  | Any other group | 1.00% | 1.25% |  |
| region | England Central/Midlands | 13.71% | 12.56% | < 0.001 |
|  | England North | 23.24% | 16.84% |  |
|  | England South (including London) | 36.79% | 29.40% |  |
|  | Northern Ireland & Scotland | 7.36% | 26.07% |  |
|  | Wales | 18.90% | 15.13% |  |
| Diabetes.combined | Without diabetes | 92.14% | 88.34% | 0.078 |
|  | Diabetic | 7.86% | 11.66% |  |
| eqv3 (£) | Lowest Tertile (≤ 17,500) | 24.58% | 36.01% | < 0.001 |
|  | Middle Tertile (> 17,500 & ≤ 32,216) | 31.44% | 29.33% |  |
|  | Highest Tertile (> 32,500) | 43.98% | 34.65% |  |

Note: mean value of the continuous variable and the proportions (%) of categorical variables in the included and excluded groups for people over 16 have been illustrated in this table

**E-iAs_ing,rice_**: Daily inorganic arsenic (iAs) intake from rice & rice products; **Sex**: Gender; **region**: Country people live; **NumChild**: Number of Children aged between 0 and 15; **age**: Age of respondent 16+; **WrkStat**: Economic status (working condition); **ethgrp5**: Ethnic group; **eqv3**: Equivalised household income; **HessCon**: Whether have any physical/mental health condition/illnesses for 12 months or more; **Diabetes.combined**: Whether respondent is diabetic; **cigsta3**: Cigarette smoking status; **dnoft**: Frequency of alcohol consumption in past 12 months (including non-drinkers); **bmival**: BMI (kg/m^2^); **whgval**: Waist-hip ratio groups

**Table S3** Modelling analysis of the categorical and continuous associations of hypertension risks (DBP add 10, SBP add 10, AP, meanPulse and the odds ratio of general hypertension) with E-iAs_ing,rice_ (µg/person/day). Data from NDNS RP 7-8 (MRC Elsie Widdowson Laboratory and NatCen Social Research 2019) for subgroup population satisfying inclusion criteria and without anti-hypertension medications (N = 477)

| Blood pressure endpoints | Quartile of E-iAs_ing,rice_ (µg/person/day) | | | | | | | Hypertension risks per 1 µg/person/day increase | p-value for trend | AIC | Contributions (%) |
| --- | --- | --- | --- | --- | --- | --- | --- | --- | --- | --- | --- |
|  | Quartile 1 (0.00-0.00) | Quartile 2  (0.00-0.565) | p-value* | Quartile 3  (0.638-3.79) | p-value* | Quartile 4 (3.79-41.8) | p-value* |  |  |  |  |
| DBP add 10 ^a^ | 1  (Referent) | 0.98  (0.94, 1.01) | 0.203 | 0.98  (0.94, 1.01) | 0.201 | 0.98  (0.94, 1.01) | 0.212 | 1.00  (0.99_7_, 1.00) | 0.736 | 3589.1 | 21.8 |
| SBP add 10 ^b^ | 1  (Referent) | 0.96  (0.93, 0.99) | 0.007 | 0.97  (0.95, 1.00) | 0.088 | 0.97  (0.94, 1.00) | 0.042 | 0.99_9_  (0.99_7_, 1.00) | 0.300 | 3926.3 | 31.3 |
| AP ^c^ | 1  (Referent) | 0.97  (0.93, 1.00) | 0.028 | 0.98  (0.95, 1.01) | 0.121 | 0.97  (0.94, 1.00) | 0.066 | 0.99_9_  (0.99_7_, 1.00) | 0.413 | 3648.8 | 24.1 |
| meanPulse ^d^ | 1  (Referent) | 1.00  (0.97, 1.04) | 0.849 | 1.00  (0.96, 1.04) | 0.990 | 0.99  (0.95, 1.03) | 0.679 | 1.00  (0.99_8_, 1.00) | 0.590 | 3573.0 | 11.9 |
| general hypertension ^e^ | 1  (Referent) | 0.48  (0.22, 1.02) | 0.058 | 0.59  (0.28, 1.23) | 0.161 | 0.47  (0.21, 1.01) | 0.057 | 0.99_6_  (0.92_6_, 1.06) | 0.913 | 366.6 | 17.2 |

Note: *compared with Quartile 1 (referent group) (0.00-0.00 µg/person/day)

The differences of the blood pressure endpoints across four quartiles were obtained from Wald tests for E-iAs_ing,rice_ coefficients, and the p-value for linear trend was obtained from analysis of variance (Anova) test with type II error where E-iAs_ing,rice_ is a continuous measure of intake and hypertension risks were expressed as a function of per unit of E-iAs_ing,rice_

**DBP add 10**: Omron valid mean diastolic blood pressure (DBP) incremented by 10 mmHg is added if anti-hypertension medication is taken (mmHg); **SBP add 10**: Omron valid mean systolic blood pressure (SBP) incremented by 10 mmHg is added if anti-hypertension medication is taken (mmHg); **AP**: Mean arterial pressure (mmHg); **meanPulse**: mean pulse pressure (mmHg); **general hypertension**: Whether participants was diagnosed as general hypertension; **E-iAs_ing,rice_**: Daily inorganic arsenic (iAs) intake from rice & rice products; **E-iAs_ing,grain_**: Daily iAs intake from grain & grain-based products; **Sex**: Gender; **FatgD**: Intake of fat per day (g) for diet only; **FolateugplussuppsD**: Intake of folate (µg) per day for both diets and supplements; **MN**: Daily intake of several micro-nutrients (Potassium (mg) including supplements, Calcium (mg) including supplements, Magnesium (mg) including supplements, Iron (mg) including supplements, Copper (mg) including supplements, Zinc (mg) including supplements, Retinol (mg) including supplements, Vitamin A (retinol equivalents) (µg) including supplements, Vitamin D (µg) including supplements, Vitamin E (mg) including supplements, Thiamin (mg) including supplements, Riboflavin (mg) including supplements, Niacin equivalent (mg) including supplements, Vitamin B6 (mg) including supplements, Vitamin B12 (µg) including supplements, Vitamin C (mg) including supplements, Iodine (µg) including supplements, Selenium (µg) including supplements); **region**: Country people live; **NumChild**: Number of Children aged between 0 and 15; **age**: Age of respondent 16+; **Quarter**: Fieldwork quarter; **qual7**: Qualifications gained; **HessCon**: Whether have any physical/mental health condition/illnesses for 12 months or more; **Diabetes.combined**: Whether respondent is diabetic; **cigsta3**: Cigarette smoking status; **bmival**: BMI (kg/m^2^); **whgval**: Waist-hip ratio groups

a: Model with DBP add 10 as dependent variable was constructed by ‘stepwise’ function in R language based on AIC values which was adjusted by age, bmival, whgval, qual7, E-iAsing,grain, HessCon and region

b: Model with SBP add 10 as dependent variable was constructed by ‘stepwise’ function in R language based on AIC values which was adjusted by age, bmival, Sex, Quarter, HessCon, NumChild, MN, whgval

c: Model with AP as dependent variable was constructed by ‘stepwise’ function in R language based on AIC values which was adjusted by age, bmival, whgval, Sex, HessCon, E-iAsing,grain, MN

d: Model with meanPulse as dependent variable was constructed by ‘stepwise’ function in R language based on AIC values which was adjusted by whgval, Sex, FolateugplussuppsD, bmival, MN, cigsta3, FatgD

e: Model with the odds ratio of general hypertension as dependent variable was constructed by ‘stepwise’ function in R language based on AIC values which was adjusted by age, bmival, Diabetes.combined, HessCon

**Table S4** Modelling analysis of the categorical and continuous association of hypertension risks (DBP add 10, SBP add 10, AP, meanPulse and the odds ratio of general hypertension) with E-iAs_ing,rice_ (µg/person/day). Data from NDNS RP 7-8 (MRC Elsie Widdowson Laboratory and NatCen Social Research 2019) with exclusion as detailed in the text (N = 598)

| model | Groups of E-iAs_ing,rice_ (µg/person/day) | | | | | | | | | | | | | | | | | | | | | | | | | | | | | | | | | | | | | | | | | | | | | | | | | | | | | | | | | | | | |
| --- | --- | --- | --- | --- | --- | --- | --- | --- | --- | --- | --- | --- | --- | --- | --- | --- | --- | --- | --- | --- | --- | --- | --- | --- | --- | --- | --- | --- | --- | --- | --- | --- | --- | --- | --- | --- | --- | --- | --- | --- | --- | --- | --- | --- | --- | --- | --- | --- | --- | --- | --- | --- | --- | --- | --- | --- | --- | --- | --- | --- | --- |
|  | Group 1 | Group 2 | p-value* | Group 3 | p-value* | Group 4 | p-value* | Group 5 | p-value* | | | | Group 6 | | | | p-value* | Group 7 | p-value* | | Group 8 | | p-value* | | Group 9 | | p-value* | | Group 10 | | p-value* | | | Group 11 | | | | | p-value* | | | Group 12 | | | p-value* | | Group 13 | | p-value* | | | | Group 14 | | | | p-value* | | Group 15 | | p-value* |
| DBP add 10 | | | | | | | | | | | | | | | | | | | | | | | | | | | | | | | | | | | | | | | | | | | | | | | | | | | | | | | | | | | | | |
| Model 1 | 1  (Referent) | 1.06  (0.99, 1.14) | 0.082 | 1.00  (0.93, 1.07) | 0.941 | 1.03  (0.96, 1.10) | 0.431 | 0.96  (0.90, 1.04) | | | | 0.314 | | | | 0.99  (0.92, 1.06) | 0.725 | 1.04  (0.97, 1.11) | 0.329 | | 0.97  (0.90, 1.04) | | 0.390 | | 1.00  (0.93, 1.07) | | 0.905 | | 1.01  (0.94, 1.09) | | | 0.744 | | | 0.96  (0.89, 1.03) | | | 0.263 | | | | | 0.94  (0.87,1.01) | | | 0.070 | | 1.01  (0.94, 1.08) | | 0.789 | | | | 1.01  (0.94, 1.08) | | | 0.887 | | 0.95  (0.88, 1.02) | | 0.157 |
| Model 2 | 1  (Referent) | 1.05  (0.99 1.12) | 0.131 | 0.99  (0.93, 1.06) | 0.785 | 1.01  (0.94, 1.08) | 0.876 | 0.94  (0.87, 1.01) | | | | 0.106 | | | | 0.95  (0.88, 1.03) | 0.193 | 1.03  (0.96, 1.11) | 0.454 | | 0.98  (0.91, 1.05) | | 0.563 | | 0.99  (0.93, 1.06) | | 0.866 | | 1.00  (0.93, 1.07) | | | 0.993 | | | 0.94  (0.88, 1.01) | | | 0.103 | | | | | 0.94  (0.87, 1.01) | | | 0.071 | | 0.98  (0.91, 1.05) | | 0.521 | | | | 0.96  (0.90, 1.03) | | | 0.231 | | 0.96  (0.98, 1.01) | | 0.296 |
| Model 3 | 1  (Referent) | 1.05  (0.99, 1.12) | 0.115 | 0.99  (0.93, 1.06) | 0.834 | 1.02  (0.96, 1.09) | 0.472 | 0.97  (0.91, 1.04) | | | | 0.354 | | | | 0.98  (0.92, 1.05) | 0.602 | 1.05  (0.99, 1.12) | 0.103 | | 0.99  (0.93, 1.06) | | 0.816 | | 1.01  (0.95, 1.08) | | 0.717 | | 1.01  (0.95, 1.08) | | | 0.721 | | | 0.95  (0.89, 1.01) | | | 0.127 | | | | | 0.96  (0.90, 1.03) | | | 0.227 | | 1.00  (0.94, 1.07) | | 0.937 | | | | 0.98  (0.91, 1.04) | | | 0.44 | | 0.98  (0.92, 1.05) | | 0.615 |
| Model 4 | 1  (Referent) | 1.06  (0.99, 1.13) | 0.084 | 1.01  (0.95, 1.08) | 0.768 | 1.03  (0.97, 1.10) | 0.336 | 0.98  (0.91, 1.04) | | | | 0.460 | | | | 0.99  (0.93, 1.06) | 0.867 | 1.06  (1.00, 1.13) | 0.055 | | 1.00  (0.94, 1.07) | | 0.912 | | 1.02  (0.95, 1.08) | | 0.615 | | 1.02  (0.96, 1.09) | | | 0.553 | | | 0.96  (0.90, 1.02) | | | 0.222 | | | | | 0.96  (0.90,1.03) | | | 0.259 | | 1.01  (0.95, 1.08) | | 0.725 | | | | 0.99  (0.93, 1.05) | | | 0.644 | | 0.99  (0.93, 1.06) | | 0.857 |
| SBP add 10 | | | | | | | | | | | | | | | | | | | | | | | | | | | | | | | | | | | | | | | | | | | | | | | | | | | | | | | | | | | | | |
| Model 1 | 1  (Referent) | 1.04  (0.97, 1.10) | 0.260 | 1.03  (0.97, 1.10) | 0.328 | 1.03  (0.97, 1.09) | 0.389 | 0.95  (0.89, 1.02) | | | 0.139 | | | | 0.97  (0.91, 1.04) | | 0.428 | 1.02  (0.96, 1.09) | 0.534 | | 0.93  (0.87, 1.00) | | 0.042 | | 0.99  (0.93, 1.05) | | 0.679 | | | 0.99  (0.93, 1.06) | | | 0.815 | | | 0.99  (0.92, 1.05) | | | | 0.652 | | | 0.93  (0.87, 1.00) | | | 0.038 | | 0.98  (0.92, 1.04) | | | 0.469 | | | 1.00  (0.93, 1.06) | | | 0.869 | | 0.95  (0.89, 1.01) | | 0.100 |
| Model 2 | 1  (Referent) | 1.01  (0.97, 1.07) | 0.639 | 1.01  (0.95, 1.07) | 0.760 | 1.00  (0.94, 1.06) | 0.955 | 0.93  (0.87, 0.99) | | | 0.017 | | | | 0.96  (0.90, 1.02) | | 0.164 | 1.02  (0.96, 1.09) | 0.497 | | 0.97  (0.91, 1.03) | | 0.289 | | 1.00  (0.94, 1.06) | | 0.945 | | | 1.00  (0.94, 1.06) | | | 0.871 | | | 0.97  (0.91, 1.03) | | | | 0.261 | | | 0.95  (0.89, 1.01) | | | 0.087 | | 0.97  (0.92, 1.03) | | | 0.320 | | | 0.96  (0.90, 1.01) | | | 0.122 | | 0.97  (0.91, 1.03) | | 0.282 |
| Model 3 | 1  (Referent) | 1.02  (0.96, 1.07) | 0.575 | 1.01  (0.95, 1.07) | 0.752 | 1.00  (0.95, 1.06) | 0.932 | 0.92  (0.87, 0.98) | | | 0.013 | | | | 0.95  (0.90, 1.01) | | 0.136 | 1.02  (0.96, 1.09) | 0.437 | | 0.97  (0.91, 1.02) | | 0.257 | | 1.00  (0.95, 1.06) | | 0.990 | | | 1.00  (0.94, 1.06) | | | 0.943 | | | 0.97  (0.91, 1.02) | | | | 0.213 | | | 0.95  (0.90, 1.01) | | | 0.092 | | 0.97  (0.92, 1.02) | | | 0.263 | | | 0.96  (0.90, 1.01) | | | 0.128 | | 0.97  (0.91, 1.03) | | 0.287 |
| Model 4 | 1  (Referent) | 1.02  (0.96, 1.07) | 0.545 | 1.02  (0.96, 1.07) | 0.547 | 1.02  (0.97, 1.08) | 0.420 | 0.94  (0.89, 1.00) | | | 0.035 | | | | 0.97  (0.92, 1.03) | | 0.297 | 1.04  (0.98, 1.09) | 0.178 | | 0.98  (0.92, 1.03) | | 0.384 | | 1.01  (0.95, 1.06) | | 0.812 | | | 1.01  (0.96, 1.07) | | | 0.728 | | | 0.98  (0.92, 1.03) | | | | 0.361 | | | 0.97  (0.92, 1.02) | | | 0.269 | | 0.98  (0.93, 1.04) | | | 0.525 | | | 0.98  (0.92, 1.03) | | | 0.345 | | 0.98  (0.92, 1.03) | | 0.389 |
| AP | | | | | | | | | | | | | | | | | | | | | | | | | | | | | | | | | | | | | | | | | | | | | | | | | | | | | | | | | | | | | |
| Model 1 | 1  (Referent) | 1.05  (0.99, 1.12) | 0.113 | 1.01  (0.95, 1.08) | 0.680 | 1.03  (0.97, 1.09) | 0.378 | 0.96  (0.90, 1.02) | | | 0.192 | | | | 0.98  (0.92, 1.05) | | 0.559 | 1.03  (0.97, 1.09) | 0.376 | | 0.95  (0.89, 1.02) | | 0.140 | | 0.99  (0.93, 1.06) | | 0.790 | | | 1.00  (0.94, 1.07) | | | 0.929 | | | | 0.97  (0.91, 1.04) | | | | 0.372 | | | 0.93  (0.88, 1.00) | | 0.038 | | 0.99  (0.93, 1.06) | | | | 0.861 | | | 1.00  (0.94, 1.06) | | 0.992 | | 0.95  (0.90, 1.01) | | 0.103 |
| Model 2 | 1  (Referent) | 1.03  (0.98, 1.09) | 0.248 | 1.00  (0.94, 1.06) | 0.984 | 1.00  (0.95, 1.07) | 0.883 | 0.93  (0.88, 1.00) | | | 0.039 | | | | 0.95  (0.90, 1.02) | | 0.157 | 1.03  (0.96, 1.09) | 0.429 | | 0.97  (0.92, 1.04) | | 0.406 | | 1.00  (0.94, 1.06) | | 0.892 | | | 1.00  (0.94, 1.06) | | | 0.946 | | | | 0.96  (0.90, 1.01) | | | | 0.134 | | | 0.94  (0.89, 1.00) | | 0.061 | | 0.98  (0.92, 1.04) | | | | 0.413 | | | 0.96  (0.90, 1.02) | | 0.149 | | 0.96  (0.90, 1.03) | | 0.263 |
| Model 3 | 1  (Referent) | 1.04  (0.98, 1.10) | 0.198 | 1.00  (0.94, 1.06) | 0.998 | 1.02  (0.97, 1.08) | 0.489 | 0.96  (0.90, 1.01) | | | 0.110 | | | | 0.97  (0.92, 1.03) | | 0.370 | 1.05  (0.99, 1.11) | 0.095 | | 0.99  (0.93, 1.04) | | 0.624 | | 1.01  (0.95, 1.07) | | 0.794 | | | 1.01  (0.95, 1.07) | | | 0.756 | | | | 0.96  (0.91, 1.02) | | | | 0.155 | | | 0.96  (0.90, 1.01) | | 0.155 | | 0.99  (0.93, 1.05) | | | | 0.685 | | | 0.97  (0.92, 1.02) | | 0.263 | | 0.98  (0.92, 1.04) | | 0.470 |
| Model 4 | 1  (Referent) | 1.04  (0.99, 1.10) | 0.111 | 1.01  (0.96, 1.07) | 0.598 | 1.04  (0.99, 1.10) | 0.166 | 0.96  (0.91, 1.02) | | | 0.202 | | | | 0.98  (0.93, 1.04) | | 0.564 | 1.05  (1.00, 1.11) | 0.064 | | 0.99  (0.94, 1.05) | | 0.801 | | 1.01  (0.96, 1.07) | | 0.596 | | | 1.02  (0.97, 1.08) | | | 0.500 | | | | 0.97  (0.92, 1.02) | | | | 0.252 | | | 0.97  (0.92, 1.03) | | 0.282 | | 1.00  (0.94, 1.05) | | | | 0.940 | | | 0.98  (0.93, 1.04) | | 0.552 | | 0.98  (0.93, 1.04) | | 0.565 |
| meanPulse | | | | | | | | | | | | | | | | | | | | | | | | | | | | | | | | | | | | | | | | | | | | | | | | | | | | | | | | | | | | | |
| Model 1 | 1  (Referent) | 0.96  (0.89, 1.02) | 0.175 | 0.99  (0.93, 1.06) | 0.850 | 0.94  (0.88, 1.01) | 0.090 | 0.94  (0.88, 1.01) | | 0.087 | | | | 0.98  (0.91, 1.04) | | | 0.451 | 0.99  (0.92, 1.05) | 0.703 | 0.98  (0.91, 1.04) | | 0.478 | | 0.98  (0.91, 1.04) | | 0.458 | | 0.97  (0.91, 1.04) | | | | 0.360 | | | | | 0.96  (0.90, 1.03) | | | | 0.269 | | | 0.94  (1.00, 0.88) | | 0.058 | | 0.95  (0.89, 1.01) | | | 0.122 | | | 0.98  (0.92, 1.04) | | 0.509 | | 0.96  (0.90, 1.03) | | 0.266 | |
| Model 2 | 1  (Referent) | 0.95  (0.89, 1.02) | 0.158 | 1.01  (0.94, 1.08) | 0.873 | 0.96  (0.89, 1.03) | 0.236 | 0.94  (0.88, 1.02) | | 0.129 | | | | 0.95  (0.88, 1.02) | | | 0.145 | 0.98  (0.91, 1.06) | 0.632 | 0.98  (0.91, 1.05) | | 0.556 | | 0.97  (0.90, 1.03) | | 0.317 | | 0.97  (0.90, 1.04) | | | | 0.368 | | | | | 0.96  (0.90, 1.03) | | | | 0.298 | | | 0.93  (0.87, 1.00) | | 0.038 | | 0.95  (0.88, 1.02) | | | 0.132 | | | 0.97  (0.90, 1.03) | | 0.322 | | 0.98  (0.91, 1.06) | | 0.639 | |
| Model 3 | 1  (Referent) | 0.94  (0.88, 1.01) | 0.084 | 1.00  (0.93, 1.07) | 0.979 | 0.96  (0.89, 1.02) | 0.180 | 0.96  (0.89, 1.02) | | 0.190 | | | | 0.97  (0.91, 1.04) | | | 0.406 | 0.99  (0.93, 1.06) | 0.810 | 0.99  (0.93, 1.06) | | 0.825 | | 0.98  (0.92, 1.04) | | 0.512 | | 0.98  (0.92, 1.05) | | | | 0.570 | | | | | 0.97  (0.91, 1.04) | | | | 0.370 | | | 0.95  (0.88, 1.01) | | 0.105 | | 0.96  (0.89, 1.02) | | | 0.200 | | | 0.98  (0.92, 1.04) | | 0.514 | | 0.99  (0.93, 1.07) | | 0.835 | |
| Model 4 | 1  (Referent) | 0.95  (0.89, 1.02) | 0.141 | 1.01  (0.94, 1.07) | 0.876 | 0.96  (0.90, 1.02) | 0.166 | 0.95  (0.89, 1.02) | | 0.133 | | | | 0.97  (0.91, 1.04) | | | 0.414 | 0.99  (0.93, 1.06) | 0.849 | 0.98  (0.92, 1.05) | | 0.600 | | 0.98  (0.91, 1.04) | | 0.441 | | 0.98  (0.92, 1.05) | | | | 0.614 | | | | | 0.97  (0.90, 1.03) | | | | 0.278 | | | 0.94  (0.89, 1.01) | | 0.107 | | 0.94  (0.88, 1.01) | | | 0.085 | | | 0.97  (0.91, 1.04) | | 0.388 | | 0.99  (0.92, 1.05) | | 0.659 | |
| Odds ratio of general hypertension | | | | | | | | | | | | | | | | | | | | | | | | | | | | | | | | | | | | | | | | | | | | | | | | | | | | | | | | | | | | | |
| Model 1 | 1  (Referent) | 1.11  (0.45, 2.75) | 0.819 | 1.51  (0.62, 3.72) | 0.367 | 1.00  (0.40, 2.48) | 0.998 | 0.42  (0.15, 1.12) | 0.088 | | | | 0.50  (0.18, 1.32) | | | | 0.167 | 1.23  (0.50, 3.04) | 0.648 | 0.42  (0.15, 1.12) | | 0.088 | | 0.80  (0.32, 2.02) | | 0.639 | | 0.63  (0.24, 1.62) | | | | 0.341 | | | | | 0.58  (0.21, 1.49) | | | | 0.259 | | | 0.24  (0.07, 0.70) | | 0.013 | | 0.63  (0.24, 1.62) | | | 0.341 | | | 0.63  (0.24, 1.62) | | 0.341 | | 0.56  (0.21, 1.44) | | 0.230 | |
| Model 2 | 1  (Referent) | 0.65  (0.19, 2.22) | 0.493 | 1.09  (0.30, 3.97) | 0.899 | 0.34  (0.09, 1.33) | 0.121 | 0.08  (0.02, 0.34) | 0.001 | | | | 0.18  (0.04, 0.76) | | | | 0.021 | 0.92  (0.23, 3.72) | 0.908 | 0.51  (0.13, 1.99) | | 0.336 | | 0.36  (0.09, 1.37) | | 0.134 | | 0.42  (0.11, 1.58) | | | | 0.202 | | | | | 0.23  (0.06, 0.84) | | | | 0.028 | | | 0.08  (0.02, 0.37) | | 0.002 | | 0.30  (0.08, 1.17) | | | 0.086 | | | 0.12  (0.03,0.46) | | 0.003 | | 0.36  (0.08, 1.60) | | 0.184 | |
| Model 3 | 1  (Referent) | 0.71  (0.22, 2.20) | 0.548 | 1.52  (0.47, 4.91) | 0.484 | 0.90  (0.28, 2.86) | 0.858 | 0.21  (0.06, 0.69) | 0.012 | | | | 0.49  (0.14, 1.63) | | | | 0.248 | 2.06  (0.66, 6.51) | 0.213 | 0.81  (0.23, 2.75) | | 0.738 | | 0.79  (0.24, 2.55) | | 0.693 | | 0.75  (0.22, 2.45) | | | | 0.628 | | | | | 0.45  (0.14, 1.40) | | | | 0.171 | | | 0.19  (0.04, 0.73) | | 0.020 | | 0.67  (0.20, 2.17) | | | 0.505 | | | 0.36  (0.15, 1.13) | | 0.084 | | 1.15  (0.34, 3.86) | | 0.819 | |
| Model 4 | 1  (Referent) | 0.72  (0.24, 2.10) | 0.541 | 1.62  (0.54, 4.94) | 0.388 | 0.93  (0.31, 2.77) | 0.896 | 0.28  (0.09, 0.88) | 0.032 | | | | 0.52  (0.16, 1.64) | | | | 0.270 | 1.62  (0.55, 4.86) | 0.387 | 0.72  (0.21, 2.35) | | 0.593 | | 0.90  (0.29, 2.74) | | 0.847 | | 0.79  (0.25, 2.40) | | | | 0.672 | | | | | 0.54  (0.17, 1.64) | | | | 0.281 | | | 0.26  (0.06, 0.95) | | 0.051 | | 0.66  (0.21, 1.98) | | | 0.457 | | | 0.47  (0.15, 1.41) | | 0.182 | | 1.09  (0.34, 3.47) | | 0.881 | |

Groups of E-iAs_ing,rice_ (µg/person/day): Group 1 (0.00-0.00); Group 2 (0.00-0.00); Group 3 (0.00-0.00); Group 4 (0.00-0.00); Group 5 (0.00-0.00); Group 6 (0.00-0.00); Group 7 (0.00-0.052); Group 8 (0.053-1.13); Group 9 (1.22-2.11); Group 10 (2.11-2.99); Group 11 (3.04-3.79); Group 12 (3.79-4.86); Group 13 (4.86-6.40); Group 14 (6.43-9.92); Group 15 (9.94-41.8)

*compared with Group 1 (referent group) (0.00-0.00 µg/person/day)

The differences of the blood pressure endpoints across 15 groups were obtained from Wald tests for E-iAs_ing,rice_ coefficients

**DBP add 10**: Omron valid mean diastolic blood pressure (DBP) incremented by 10 mmHg is added if anti-hypertension medication is taken (mmHg); **SBP add 10**: Omron valid mean systolic blood pressure (SBP) incremented by 10 mmHg is added if anti-hypertension medication is taken (mmHg); **AP**: Mean arterial pressure (mmHg); **meanPulse**: mean pulse pressure (mmHg); **general hypertension**: Whether participants was diagnosed as general hypertension; **E-iAs_ing,rice_**: Daily inorganic arsenic (iAs) intake from rice & rice products; **E-iAs_ing,water_**: Daily iAs intake from drinking water; **E-iAs_ing,grain_**: Daily iAs intake from grain & grain-based products; **surveyyr**: NDNS RP 7-8 Survey year; **Sex**: Gender; **EnergyDkJ**: Intake of total energy per day (KJ) for diet only; **ProteingD**: Intake of protein per day (g) for diet only; **FatgD**: Intake of fat per day (g) for diet only; **GlucosegD**: Intake of glucose per day (g) for diet only; **SodiummgD**: Intake of sodium per day (mg) for diet only; **FolateugplussuppsD**: Intake of folate (µg) per day for both diets and supplements; **MN**: Daily intake of several micro-nutrients (Potassium (mg) including supplements, Calcium (mg) including supplements, Magnesium (mg) including supplements, Iron (mg) including supplements, Copper (mg) including supplements, Zinc (mg) including supplements, Retinol (mg) including supplements, Vitamin A (retinol equivalents) (µg) including supplements, Vitamin D (µg) including supplements, Vitamin E (mg) including supplements, Thiamin (mg) including supplements, Riboflavin (mg) including supplements, Niacin equivalent (mg) including supplements, Vitamin B6 (mg) including supplements, Vitamin B12 (µg) including supplements, Vitamin C (mg) including supplements, Iodine (µg) including supplements, Selenium (µg) including supplements); **region**: Country people live; **NumChild**: Number of Children aged between 0 and 15; **age**: Age of respondent 16+; **SalHowC**: How often salt added during cooking; **Quarter**: Fieldwork quarter; **qual7**: Qualifications gained; **WrkStat**: Economic status (working condition); **ethgrp5**: Ethnic group; **eqv3**: Equivalized household income; **HessCon**: Whether have any physical/mental health condition/illnesses for 12 months or more; **Diabetes.combined**: Whether respondent is diabetic; **cigsta3**: Cigarette smoking status; **dnoft**: Frequency of alcohol consumption in past 12 months (including non-drinkers); **bmival**: BMI (kg/m^2^); **whgval**: Waist-hip ratio groups

For DBP ad 10: Model 1: crude with DBP add 10 only (univariate model); Model 2: full model, adjusted by E-iAs_ing,water_, E-iAs_ing,grain_, age, bmival, cigsta3, region, Diabetes.combined, dnoft, eqv3, ethgrp5, SalHowC, HessCon, MN, NumChild, qual7, Quarter, Sex, surveyyr, whgval, WrkStat, EnergyDkJ, ProteingD, FatgD, GlucosegD, SodiummgD, FolateugplussuppsD; Model 3: adjusted by variables with p-value lower than 0.2 in the univariate analysis: E-iAs_ing,water_, E-iAs_ing,grain_, age, bmival, cigsta3, region, Diabetes.combined, dnoft, HessCon, MN, NumChild, qual7, Sex, whgval, WrkStat; and Model 4: constructed by ‘stepwise’ function in R language based on AIC values which was adjusted by age, bmival, whgval, qual7, iAs.grain, HessCon and region

For SBP add 10: Model 1: crude with SBP add 10 only (univariate model); Model 2: full model, adjusted by E-iAs_ing,water_, E-iAs_ing,grain_, age, bmival, cigsta3, region, Diabetes.combined, dnoft, eqv3, ethgrp5, SalHowC, HessCon, MN, NumChild, qual7, Quarter, Sex, surveyyr, whgval, WrkStat, EnergyDkJ, ProteingD, FatgD, GlucosegD, SodiummgD, FolateugplussuppsD; Model 3: adjusted by variables with p-value lower than 0.2 in the univariate analysis: E-iAs_ing,water_, age, bmival, cigsta3, region, Diabetes.combined, dnoft, ethgrp5, HessCon, MN, NumChild, Quarter, Sex, surveyyr, whgval, WrkStat, FolateugplussuppsD; and Model 4: constructed by ‘stepwise’ function in R language based on AIC values which was adjusted by age, bmival, Sex, Quarter, HessCon, NumChild, MN, whgval

For AP: Model 1: crude with AP only (univariate model); Model 2: full model, adjusted by E-iAs_ing,water_, E-iAs_ing,grain_, age, bmival, cigsta3, region, Diabetes.combined, dnoft, eqv3, ethgrp5, SalHowC, HessCon, MN, NumChild, qual7, Quarter, Sex, surveyyr, whgval, WrkStat, EnergyDkJ, ProteingD, FatgD, GlucosegD, SodiummgD, FolateugplussuppsD; Model 3: adjusted by variables with p-value lower than 0.2 in the univariate analysis: E-iAs_ing,water_, E-iAs_ing,grain_, age, bmival, cigsta3, region, Diabetes.combined, dnoft, HessCon, MN, NumChild, qual7, Sex, whgval, FolateugplussuppsD; Model 4: constructed by ‘stepwise’ function in R language based on AIC values which was adjusted by age, bmival, whgval, Sex, HessCon, iAs.grain, MN

For meanPulse: Model 1: crude with meanPulse only (univariate model); Model 2: full model, adjusted by E-iAs_ing,water_, E-iAs_ing,grain_, age, bmival, cigsta3, region, Diabetes.combined, dnoft, eqv3, ethgrp5, SalHowC, HessCon, MN, NumChild, qual7, Quarter, Sex, surveyyr, whgval, WrkStat, EnergyDkJ, ProteingD, FatgD, GlucosegD, SodiummgD, FolateugplussuppsD; Model 3: adjusted by variables with p-value lower than 0.2 in the univariate analysis E-iAs_ing,water_, E-iAs_ing,grain_, bmival, cigsta3, region, Diabetes.combined, dnoft, ethgrp5, MN, qual7, Sex, whgval, WrkStat, ProteingD, FatgD, GlucosegD, FolateugplussuppsD; Model 4: constructed by ‘stepwise’ function in R language based on AIC values which was adjusted by whgval, Sex, FolateugplussuppsD, bmival, MN, cigsta3, FatgD

For the odds ratio of general hypertension: Model 1: crude with the odds ratio of general hypertension only (univariate model); Model 2: full model, adjusted by E-iAs_ing,water_, E-iAs_ing,grain_, age, bmival, cigsta3, region, Diabetes.combined, dnoft, eqv3, ethgrp5, SalHowC, HessCon, MN, NumChild, qual7, Quarter, Sex, surveyyr, whgval, WrkStat, EnergyDkJ, ProteingD, FatgD, GlucosegD, SodiummgD, FolateugplussuppsD; Model 3: adjusted by variables with p-value lower than 0.2 in the univariate analysis E-iAs_ing,water_, E-iAs_ing,grain_, age, bmival, cigsta3, Diabetes.combined, dnoft, eqv3, HessCon, NumChild, qual7, whgval, WrkStat; Model 4: constructed by ‘stepwise’ function in R language based on AIC values which was adjusted by age, bmival, Diabetes.combined, HessCon


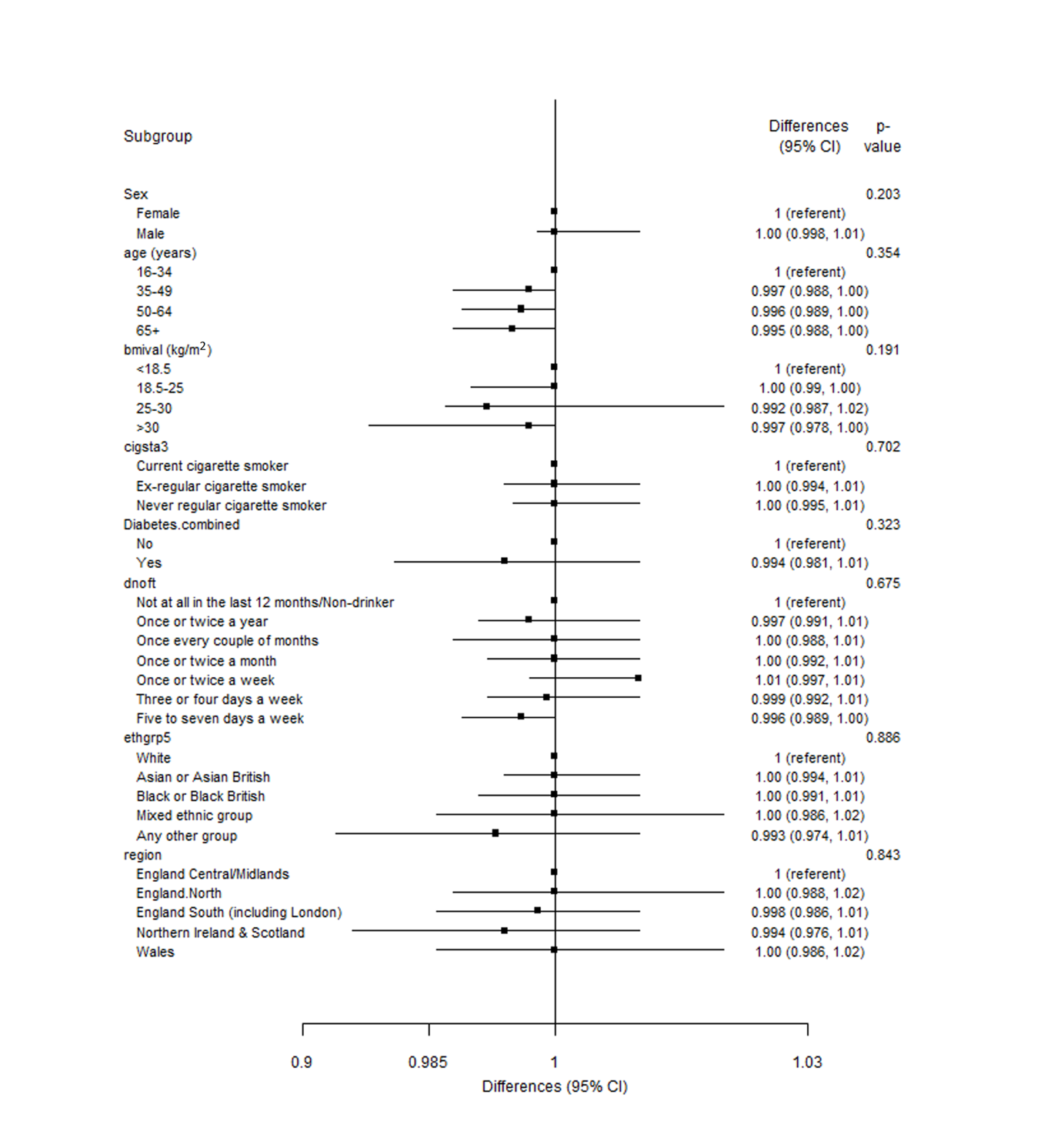


**Fig. S1** Percent changes and 95% confidence intervals (CIs) for DBP add 10 for an increase of 1 µg/person/day E-iAs_ing,rice_ by participants’ key factors (Data from NDNS RP 7-8 (MRC Elsie Widdowson Laboratory and NatCen Social Research 2019) with population satisfying inclusion criteria as detailed in the text (N = 598)). Filled squares represent the DBP add 10 for an increase of 1 µg/person/day E-iAs_ing,rice_. Horizontal lines represent their 95% CIs

p-values for the interaction of E-iAs_ing,rice_ with participants’ characteristics were obtained by adding an interaction term between E-iAs_ing,rice_ and the corresponding participants’ characteristic in the multivariable model, computed by an analysis of variance (Anova) test with type II error to account for the complex design

Model with DBP add 10 as dependent variable was adjusted by age, bmival, whgval, qual7, E-iAs_ing,grain_, HessCon and region

**DBP add 10**: Omron valid mean diastolic blood pressure (DBP) incremented by 10 mmHg is added if anti-hypertension medication is taken (mmHg); **E-iAs_ing,rice_**: Daily inorganic arsenic (iAs) intake from rice & rice products; **E-iAs_ing,grain_**: Daily iAs intake from grain & grain-based products; **Sex**: Gender; **region**: Country people live; **age**: Age of respondent 16+; **qual7**: Qualifications gained; **ethgrp5**: Ethnic group; **HessCon**: Whether have any physical/mental health condition/illnesses for 12 months or more; **Diabetes.combined**: Whether respondent is diabetic; **cigsta3**: Cigarette smoking status; **dnoft**: Frequency of alcohol consumption in past 12 months (including non-drinkers); **bmival**: BMI (kg/m^2^); **whgval**: Waist-hip ratio groups


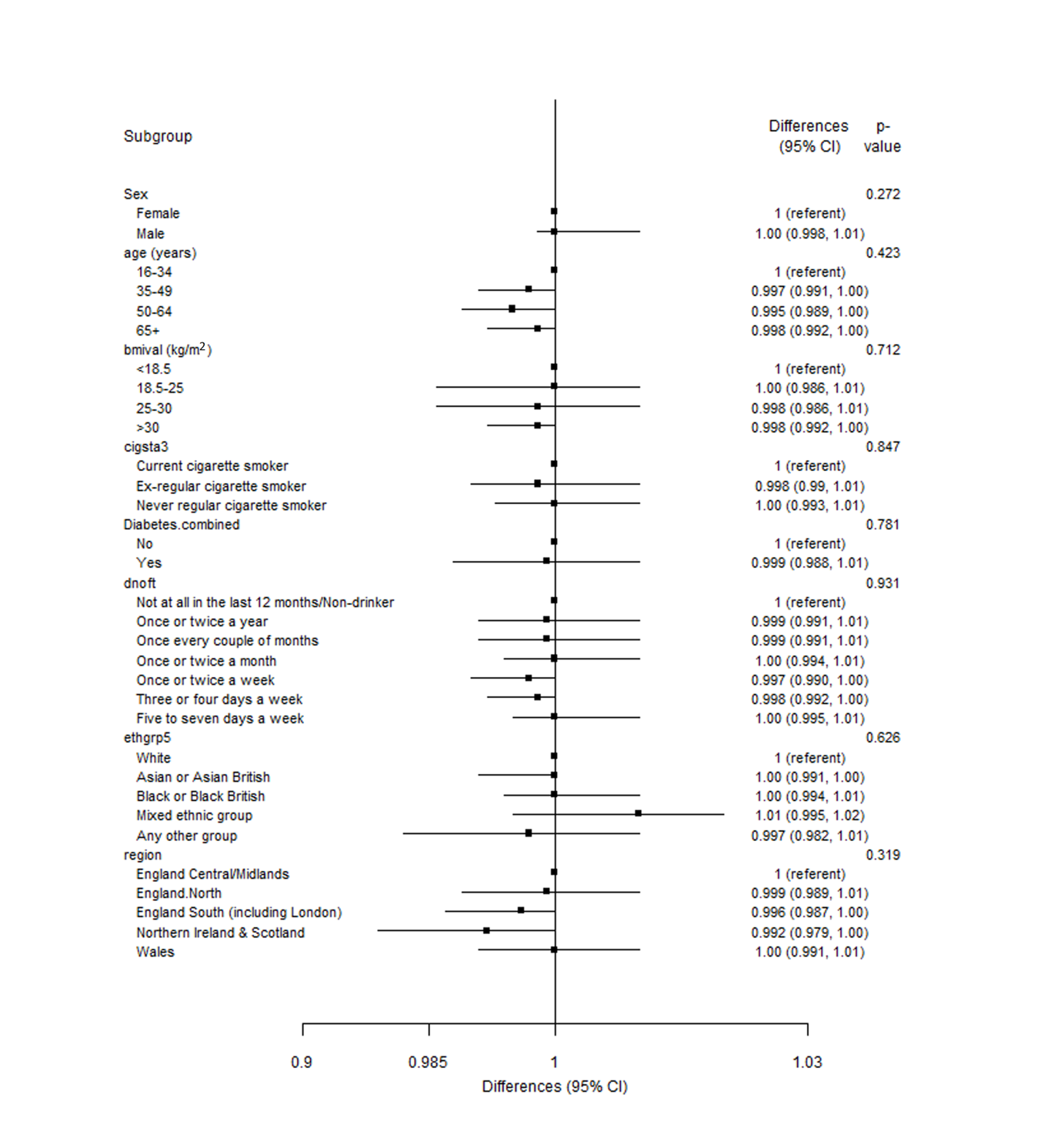


**Fig. S2** Percent changes and 95% confidence intervals (CIs) for SBP add 10 for an increase of 1 µg/person/day E-iAs_ing,rice_ by participants’ key factors (Data from NDNS RP 7-8 (MRC Elsie Widdowson Laboratory and NatCen Social Research 2019) with population satisfying inclusion criteria as detailed in the text (N = 598)). Filled squares represent the SBP add 10 for an increase of 1 µg/person/day E-iAs_ing,rice_. Horizontal lines represent their 95% CIs

p-values for the interaction of E-iAs_ing,rice_ with participants’ characteristics were obtained by adding an interaction term between E-iAs_ing,rice_ and the corresponding participants’ characteristic in the multivariable model, computed by the analysis of variance (Anova) test with type II error to account for the complex design

Model with SBP add 10 as dependent variable was adjusted by age, bmival, Sex, Quarter, HessCon, NumChild, MN, whgval

**SBP add 10**: Omron valid mean systolic blood pressure (SBP) incremented by 10 mmHg is added if anti-hypertension medication is taken (mmHg); **E-iAs_ing,rice_**: Daily inorganic arsenic (iAs) intake from rice & rice products; **Sex**: Gender; **MN**: Daily intake of several micro-nutrients (Potassium (mg) including supplements, Calcium (mg) including supplements, Magnesium (mg) including supplements, Iron (mg) including supplements, Copper (mg) including supplements, Zinc (mg) including supplements, Retinol (mg) including supplements, Vitamin A (retinol equivalents) (µg) including supplements, Vitamin D (µg) including supplements, Vitamin E (mg) including supplements, Thiamin (mg) including supplements, Riboflavin (mg) including supplements, Niacin equivalent (mg) including supplements, Vitamin B6 (mg) including supplements, Vitamin B12 (µg) including supplements, Vitamin C (mg) including supplements, Iodine (µg) including supplements, Selenium (µg) including supplements); **region**: Country people live; **NumChild**: Number of Children aged between 0 and 15; **age**: Age of respondent 16+; **Quarter**: Fieldwork quarter; **qual7**: Qualifications gained; **ethgrp5**: Ethnic group; **HessCon**: Whether have any physical/mental health condition/illnesses for 12 months or more; **Diabetes.combined**: Whether respondent is diabetic; **cigsta3**: Cigarette smoking status; **dnoft**: Frequency of alcohol consumption in past 12 months (including non-drinkers); **bmival**: BMI (kg/m^2^)


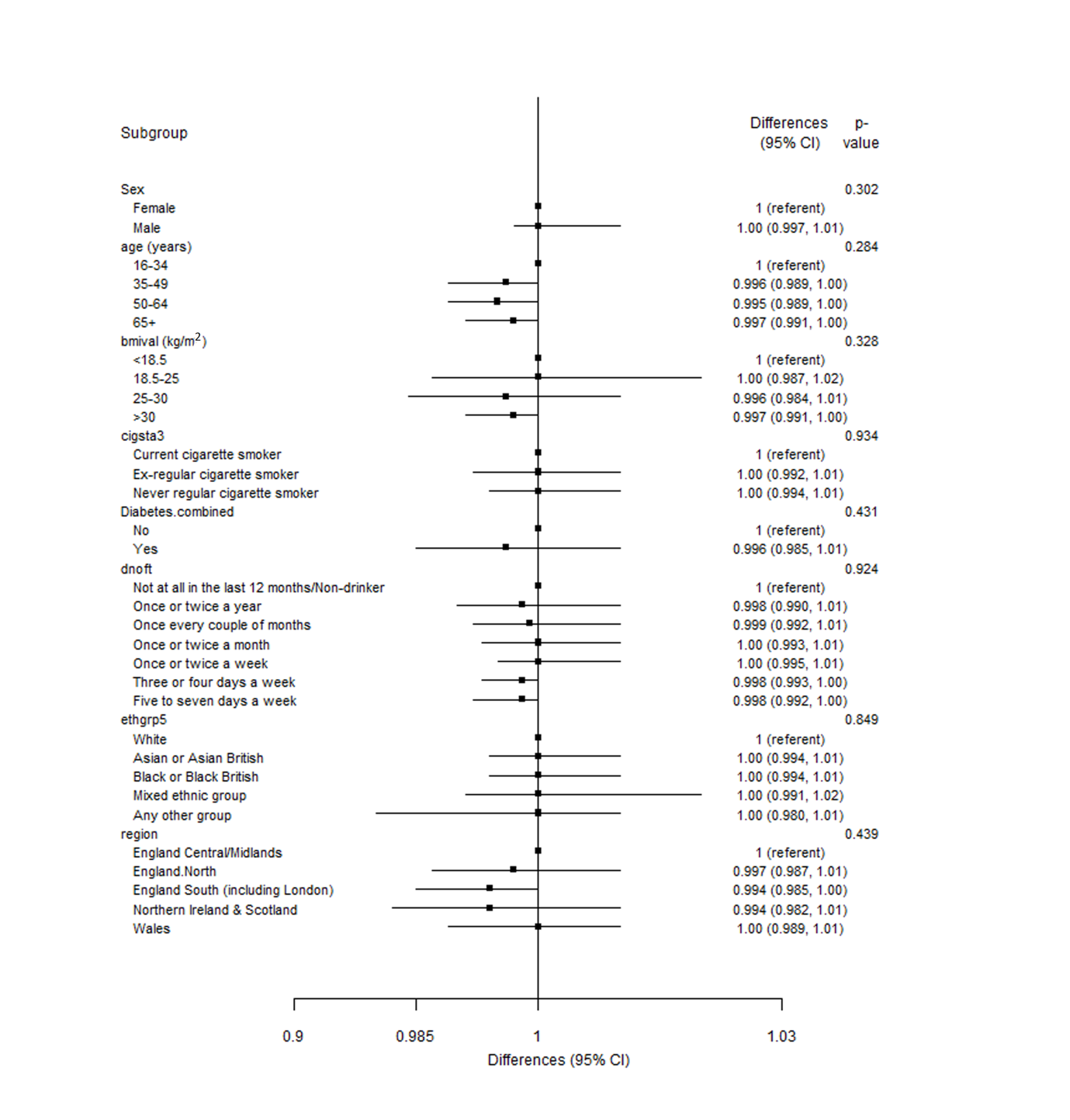


**Fig. S3** Percent changes and 95% confidence intervals (CIs) for AP for an increase of 1 µg/person/day E-iAs_ing,rice_ by participants’ key factors (Data from NDNS RP 7-8 (MRC Elsie Widdowson Laboratory and NatCen Social Research 2019) with population satisfying inclusion criteria as detailed in the text (N = 598)). Filled squares represent the AP for an increase of 1 µg/person/day E-iAs_ing,rice_. Horizontal lines represent their 95% CIs

p-values for the interaction of E-iAs_ing,rice_ with participants’ characteristics were obtained by adding an interaction term between E-iAs_ing,rice_ and the corresponding participants’ characteristic in the multivariable model, computed by the analysis of variance (Anova) test with type II error to account for the complex design

Model with AP as dependent variable was adjusted by age, bmival, whgval, Sex, HessCon, E-iAs_ing,grain_, MN

**AP**: Mean arterial pressure (mmHg); **E-iAs_ing,rice_**: Daily inorganic arsenic (iAs) intake from rice & rice products; **E-iAs_ing,grain_**: Daily iAs intake from grain & grain-based products; **Sex**: Gender; **MN**: Daily intake of several micro-nutrients (Potassium (mg) including supplements, Calcium (mg) including supplements, Magnesium (mg) including supplements, Iron (mg) including supplements, Copper (mg) including supplements, Zinc (mg) including supplements, Retinol (mg) including supplements, Vitamin A (retinol equivalents) (µg) including supplements, Vitamin D (µg) including supplements, Vitamin E (mg) including supplements, Thiamin (mg) including supplements, Riboflavin (mg) including supplements, Niacin equivalent (mg) including supplements, Vitamin B6 (mg) including supplements, Vitamin B12 (µg) including supplements, Vitamin C (mg) including supplements, Iodine (µg) including supplements, Selenium (µg) including supplements); **region**: Country people live; **age**: Age of respondent 16+; **ethgrp5**: Ethnic group; **HessCon**: Whether have any physical/mental health condition/illnesses for 12 months or more; **Diabetes.combined**: Whether respondent is diabetic; **cigsta3**: Cigarette smoking status; **dnoft**: Frequency of alcohol consumption in past 12 months (including non-drinkers); **bmival**: BMI (kg/m^2^); **whgval**: Waist-hip ratio groups


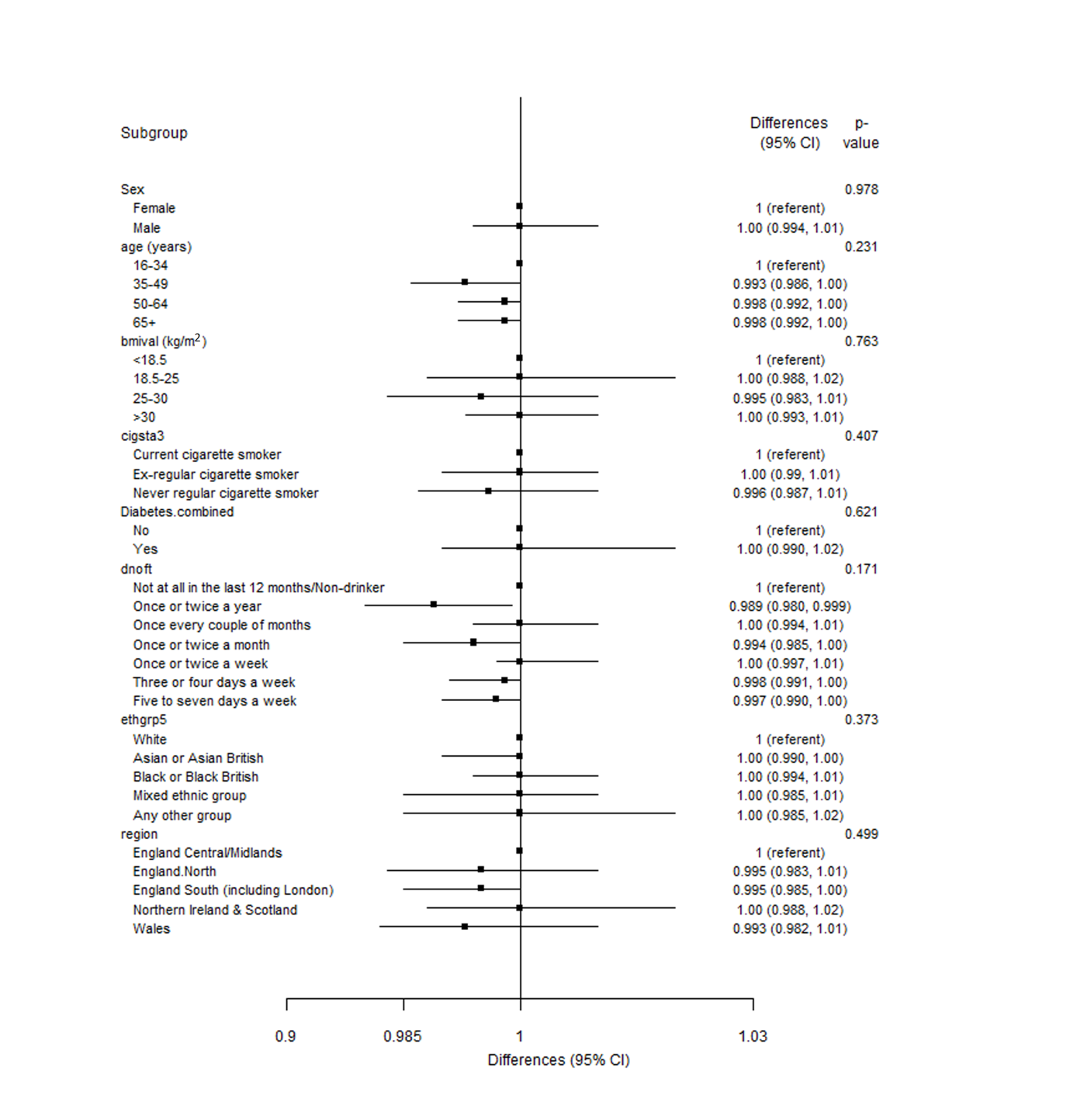


**Fig. S4** Percent changes and 95% confidence intervals (CIs) for meanPulse for an increase of 1 µg/person/day E-iAs_ing,rice_ by participants’ key factors (Data from NDNS RP 7-8 (MRC Elsie Widdowson Laboratory and NatCen Social Research 2019) with population satisfying inclusion criteria as detailed in the text (N = 598)). Filled squares represent the meanPulse for an increase of 1 µg/person/day E-iAs_ing,rice_. Horizontal lines represent their 95% CIs

p-values for the interaction of E-iAs_ing,rice_ with participants’ characteristics were obtained by adding an interaction term between E-iAs_ing,rice_ and the corresponding participants’ characteristic in the multivariable model, computed by the analysis of variance (Anova) test with type II error to account for the complex design

Model with meanPulse as dependent variable was adjusted by whgval, Sex, FolateugplussuppsD, bmival, MN, cigsta3, FatgD

**meanPulse**: Mean pulse pressure (mmHg); **E-iAs_ing,rice_**: Daily inorganic arsenic (iAs) intake from rice & rice products; **Sex**: Gender; **FatgD**: Intake of fat per day (g) for diet only; **FolateugplussuppsD**: Intake of folate (µg) per day for both diets and supplements; **MN**: Daily intake of several micro-nutrients (Potassium (mg) including supplements, Calcium (mg) including supplements, Magnesium (mg) including supplements, Iron (mg) including supplements, Copper (mg) including supplements, Zinc (mg) including supplements, Retinol (mg) including supplements, Vitamin A (retinol equivalents) (µg) including supplements, Vitamin D (µg) including supplements, Vitamin E (mg) including supplements, Thiamin (mg) including supplements, Riboflavin (mg) including supplements, Niacin equivalent (mg) including supplements, Vitamin B6 (mg) including supplements, Vitamin B12 (µg) including supplements, Vitamin C (mg) including supplements, Iodine (µg) including supplements, Selenium (µg) including supplements); **region**: Country people live; **age**: Age of respondent 16+; **ethgrp5**: Ethnic group; **Diabetes.combined**: Whether respondent is diabetic; **cigsta3**: Cigarette smoking status; **dnoft**: Frequency of alcohol consumption in past 12 months (including non-drinkers); **bmival**: BMI (kg/m^2^); **whgval**: Waist-hip ratio groups


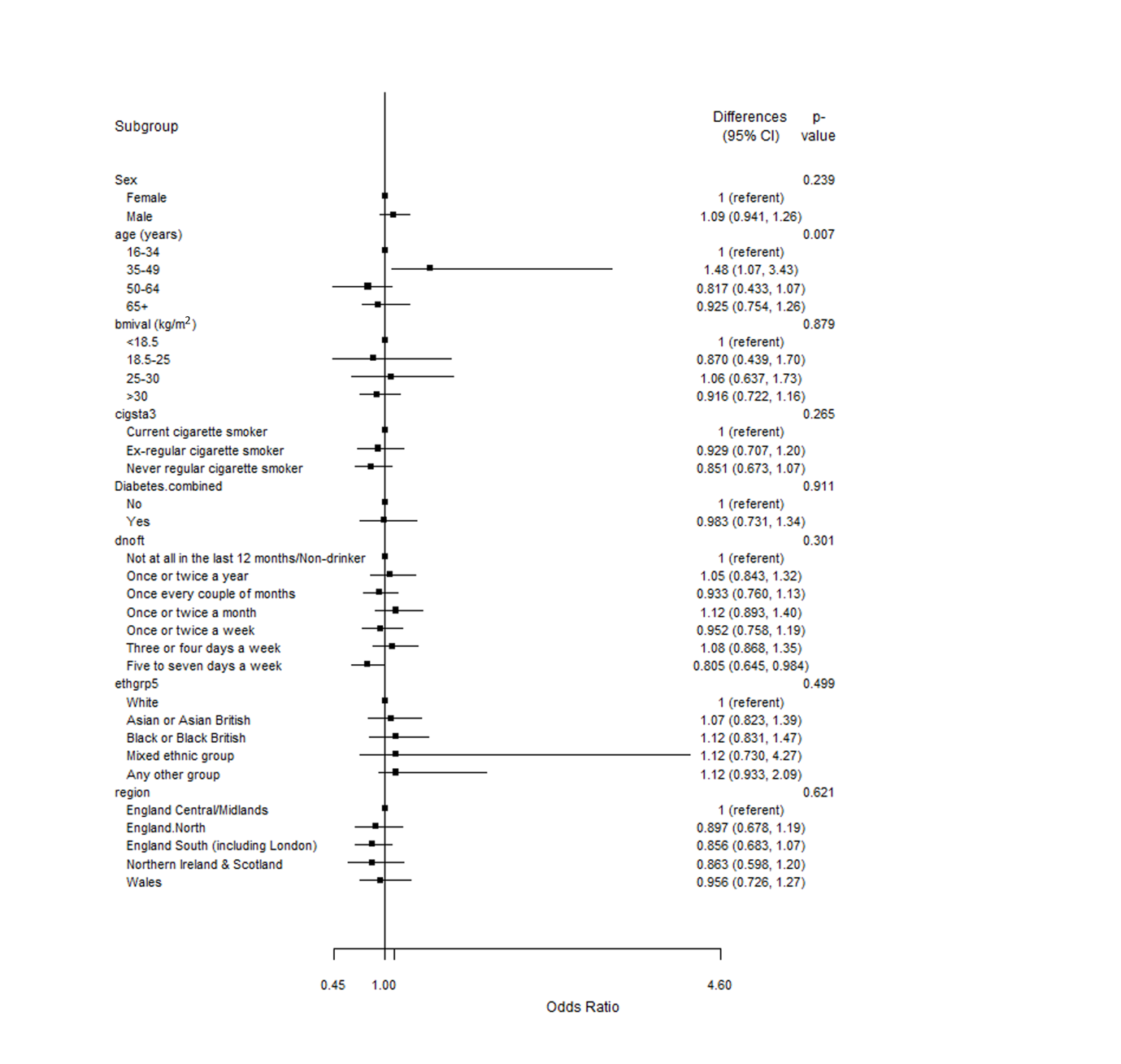
 **Fig. S5** Odds Ratios and 95% confidence intervals (CIs) for general hypertension for an increase of 1 µg/person/day E-iAs_ing,rice_ by participants’ key factors (Data from NDNS RP 7-8 (MRC Elsie Widdowson Laboratory and NatCen Social Research 2019) with population satisfying inclusion criteria as detailed in the text (N = 598)). Filled squares represent the odds ratio of general hypertension for an increase of 1 µg/person/day E-iAs_ing,rice_. Horizontal lines represent their 95% CIs

p-values for the interaction of E-iAs_ing,rice_ with participants’ characteristics were obtained by adding an interaction term between E-iAs_ing,rice_ and the corresponding participants’ characteristic in the multivariable model, computed by the analysis of variance (Anova) test with type II error to account for the complex design

Model with the odds ratio of general hypertension as dependent variable was adjusted by age, bmival, Diabetes.combined, HessCon

**general hypertension**: Whether participants was diagnosed as general hypertension; **E-iAs_ing,rice_**: Daily inorganic arsenic (iAs) intake from rice & rice products; **Sex**: Gender; **region**: Country people live; **age**: Age of respondent 16+; **ethgrp5**: Ethnic group; **HessCon**: Whether have any physical/mental health condition/illnesses for 12 months or more; **Diabetes.combined**: Whether respondent is diabetic; **cigsta3**: Cigarette smoking status; **dnoft**: Frequency of alcohol consumption in past 12 months (including non-drinkers); **bmival**: BMI (kg/m^2^)
